# IP_3_R2-mediated inter-organelle calcium signaling suppresses melanosome degradation

**DOI:** 10.1371/journal.pbio.3003971

**Published:** 2026-08-25

**Authors:** Suman Saurav, Anuradha Jadon, Rajender K. Motiani

**Affiliations:** Laboratory of Calciomics and Systemic Pathophysiology (LCSP), Regional Centre for Biotechnology (RCB), Faridabad, Haryana, India; Institute for Stem Cell Science and Regenerative Medicine, INDIA

## Abstract

Organelle turnover is fundamental to cellular homeostasis and regulates both physiological processes and pathological outcomes. Skin pigmentation is determined by the balance between melanosome biogenesis and degradation. However, the mechanisms governing melanosome degradation, i.e., melanophagy remain largely unappreciated. Here, we reveal Inositol 1,4,5-trisphosphate receptor 2 (IP_3_R2) as a selective suppressor of melanophagy. To enable real-time monitoring of melanophagy, we developed and characterized two novel ratiometric live-cell imaging probes. Using a multi-pronged strategy combining live-cell imaging with the probes, biochemical studies, ultrastructural analyses, molecular approaches, and calcium imaging, we demonstrate that IP_3_R2 suppresses melanophagy. Importantly, in vivo studies in zebrafish model and meta-analysis of human skin microarrays substantiate the physiological relevance of IP_3_R2 in pigmentation. Mechanistically, IP_3_R2 depletion impairs mitochondrial Ca^2+^ uptake, elevates the ADP/ATP ratio and initiates melanophagy. Concurrently, IP_3_R2 loss enhances ER–lysosome contacts, increases lysosomal Ca^2+^ levels via TMEM165, and activates TRPML1 and nuclear translocation of TFEB. This in turn transcriptionally induces melanophagy receptor and E3 ligase. Collectively, IP_3_R2 acts as a critical determinant of melanophagy and a potential therapeutic target for pigmentary disorders and skin malignancies.

## Abstract 2

Skin pigmentation depends on the balance of melanosome biogenesis and degradation, but the mechanisms of melanophagy are incompletely understood. This study shows that IP3R2 regulates melanophagy in zebrafish and mammalian cells, implicating a mechanism involving calcium signaling from the endoplasmic reticulum to the mitochondria and lysosomes.

## Introduction

Human skin pigmentation serves as a defense mechanism against harmful ultraviolet (UV) rays. Dysregulated pigmentation can lead to skin cancers and results in pigmentary disorders [1]. The damaging effects of ultraviolet radiation are shielded by a natural photoprotective pigment, i.e., melanin. It is produced through the process of melanogenesis within pigment-producing cells called melanocytes [2]. Melanogenesis occurs in the specialized lysosome-related organelle, i.e., melanosomes [3,4]. Skin pigmentation is an outcome of homeostatic balance between melanosome biogenesis, melanogenesis within melanosomes and melanosome degradation, i.e., melanophagy. Although molecular mechanisms driving melanosome biogenesis are somewhat understood, the signaling cascades that drive melanophagy remain largely unappreciated. Recent studies have identified selective autophagy to regulate turnover and homeostasis of other lysosome-related organelles (LROs). This cargo-specific autophagy governs the quality control of other LROs, including platelet dense granules and secretory granules, thereby positioning melanophagy within a broader LRO quality-control framework governed by specific regulatory signals [5,6].

Calcium (Ca^2+^) signaling is emerging as a critical regulator of pigmentation [1,7,8]. Ca^2+^ within melanocytes contributes to their function thereby regulating dendricity and pigmentation [9]. Ca^2+^ helps in defining skin color by regulating tyrosinase activity, an enzyme critical for melanogenesis [10]. Further, UV rays activate G protein-coupled receptor (GPCR) in melanocytes and initiate Phospholipase C (PLC) signaling cascade. This in turn activates transient receptor potential ankyrin subtype 1 (TRPA1) and transient receptor potential channel vanilloid subtype 1 (TRPV1) channels leading to Ca^2+^ influx and induction of melanin synthesis [11,12]. The UV-induced Ca^2+^ influx in melanocytes stimulates melanosome transfer to keratinocytes which in turn provides photoprotection [13,14]. While the role of extracellular Ca^2+^ influx in regulating skin pigmentation is being appreciated, the role of organelle Ca^2+^ signaling is poorly understood.

We and others have recently demonstrated a crucial role of endoplasmic reticulum Ca^2+^ signaling in regulating skin pigmentation and controlling melanoma tumor progression [15–17]. Further, mitochondrial matrix Ca^2+^ uptake via Mitochondrial Ca^2+^ Uniporter (MCU) complex drives skin pigmentation [18] by regulating melanosome biogenesis. On the other hand, outer mitochondrial membrane Ca^2+^ uptake channel, VDAC negatively regulates pigmentation by controlling transcription of genes involved in melanogenesis [19]. Interestingly, mitochondria tether with melanosomes and that in turn regulates melanogenesis [20,21]. Therefore, emerging literature suggests that organelle Ca^2+^ signaling can contribute to pigmentation. However, the functional significance of Ca^2+^-driven inter-organelle crosstalk in regulating pigmentation is still unappreciated and that in melanophagy is completely unknown.

Here, we reveal that IP_3_R2 regulates Ca^2+^-driven ER-Mitochondria and ER-lysosomal crosstalk that, in turn, suppresses melanophagy and enhances pigmentation. Analysis of two independent microarray datasets showed that IP_3_R2 expression is directly associated with pigmentation levels. Targeted siRNA screening for IP_3_R isoforms identified IP_3_R2 as a negative regulator of pigmentation. Further, our overexpression and rescue experiments with wild-type and pore-dead IP_3_R2 mutant demonstrated that IP_3_R2-mediated ER Ca^2+^ release is essential for modulating pigmentation. Importantly, our in vivo experiments in zebrafish model recapitulated the in vitro observations at the organism level. Our robust mechanistic studies show that IP_3_R2 silencing enhances ER and lysosome proximity. This in turn augments lysosomal Ca^2+^ levels and decreases lysosomal pH. It subsequently activates lysosomal TRPML1 channel and stimulates nuclear translocation of TFEB transcription factor. TFEB drives transcription of the two known melanophagy drivers and thereby stimulates melanophagy. In summary, we have identified a novel signaling module that regulates melanophagy process and thereby modulate pigmentation. Since pigmentation protects from harmful UV radiations, this signaling cascade may offer likely therapeutic targets to manage pigmentary disorders and skin cancers.

## Results

### IP_3_R2, but not IP_3_R1 and IP_3_R3, positively regulates pigmentation

**Fig 1:**
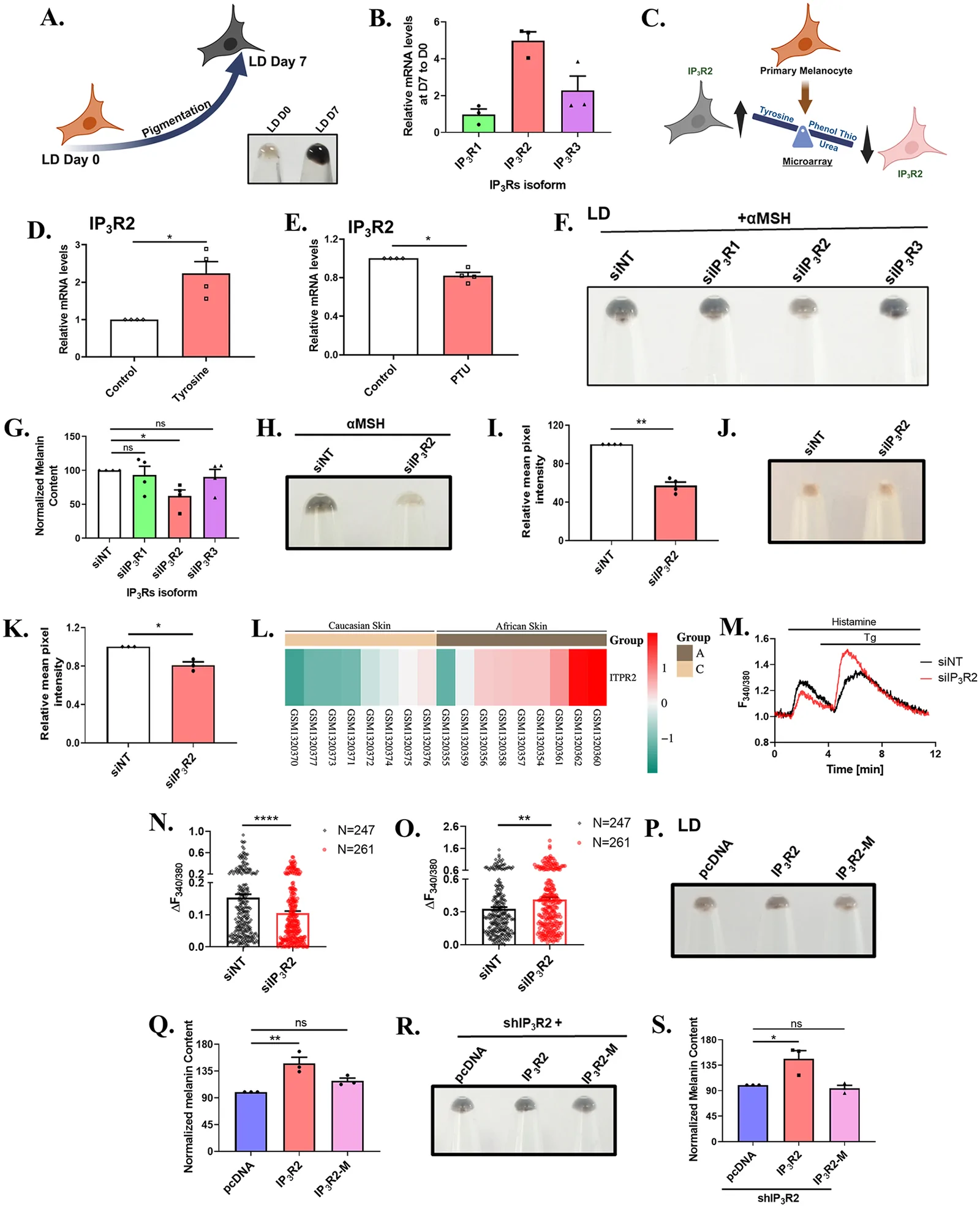
IP_3_R2 is a positive regulator of pigmentation. **(A)** Pictorial representation and cell pellet image demonstrating increased pigmentation of B16 cells over seven days, from day0 to day7. Created in BioRender. Motiani, R. (2026) https://BioRender.com/o9pmka1. **(B)** qRT-PCR analysis showing quantification of IP_3_R1, IP_3_R2, and IP_3_R3 mRNA in B16 cells (*N* = 3). **(C)** Microarray analysis of IP_3_R2 in primary human melanocyte showing pictorial representation of microarray stimulated with L-tyrosine and Phenylthiourea. Created in BioRender. Motiani, R. (2026) https://BioRender.com/07kdt7i. **(D)** qRT-PCR analysis showing increase in IP_3_R2 mRNA expression after 72 h of treatment with 1mM L-Tyrosine (*N* = 4). **(E)** qRT-PCR analysis showing decrease in IP_3_R2 mRNA expression after 72 h of treatment with 200 µM Phenylthiourea (*N* = 4). **(F)** Representative image of pellet pictures demonstrates B16 cells of LD day6, transfected with either non-targeted siRNA or siRNA specifically targeting IP_3_ receptor type1, type2, and type3 on LD day3. **(G)** Bar graph showing melanin content estimation of B16 cells harvested 72 h post siRNA transfection at LD day6 (*N* = 4). **(H)** Representative image of pellet pictures shows B16 cells 72 h after transfection with either non-targeted siRNA or siRNA specifically targeting IP_3_R2, along with a 48 h exposure to 1 μM αMSH. **(I)** Bar graph showing mean pixel intensity of B16 cells harvested 72 h post-siRNA transfection in addition to 48 h exposure to 1 μM αMSH (*N* = 4). **(J)** Representative image of pellet picture showing primary human melanocytes 72 h post-transfection with either non-targeted siRNA or siRNA specifically targeting IP_3_R2. **(K)** Bar graph showing mean pixel intensity of lightly pigmented primary human melanocytes harvested 72 h post-siRNA transfection (*N* = 3). **(L)** Heatmap highlighting the expression pattern of the IP_3_R2 gene in African vs. White skin (GSE54638). **(M)** Representative traces of Fura2AM-based Ca^2+^ imaging in B16 cells stimulated with the Histamine followed by Tg after 72 h of siRNA transfection. **(N)** Bar graph showing the quantification of Ca^2+^ imaging traces stimulated with 50 µM Histamine, where ‘*N*’ denotes the total number of cells imaged. **(O)** Bar graph showing the quantification of Ca^2+^ imaging traces stimulated with 2 µM Tg, where ‘*N*’ denotes the total number of cells imaged. **(P)** Representative image of pellet picture showing B16 cells transfected with IP_3_R2 and IP_3_R2-M in the LD model system. **(Q)** Bar graph showing the melanin content in B16 cells transfected with IP_3_R2 and IP_3_R2-M (*N* = 4). **(R)** Representative image of pellet picture showing B16 cells overexpressed with IP_3_R2 and IP_3_R2-M in stable IP_3_R2 knockdown background. **(S)** Bar graph showing the melanin content in B16 cells overexpressed with IP_3_R2 and IP_3_R2-M in stable IP_3_R2 knockdown background (*N* = 3). Data presented are mean ± SEM. For statistical analysis, Dunnett’s multiple comparisons test was performed for panels G, Q, S, while one-sample *t* test was performed for panels D, E, I, K and an Unpaired *t* test was performed for panels N, O using GraphPad Prism software. Here, ‘ns’ means non-significant; * *p* < 0.05, ** *p* < 0.01 and *** *p* < 0.001.

We have recently reported that physiological melanogenic stimuli α-melanocyte-stimulating hormone (αMSH) generates IP_3_ thereby inducing release of Ca^2+^ from the IP_3_ receptors (IP_3_Rs) localized on the endoplasmic reticulum [17, 18]. However, the functional relevance of IP_3_Rs in the pigmentation remains completely unknown. We had earlier performed two independent microarrays to identify novel regulators of pigmentation [17,22]. The first microarray was performed on B16 mouse melanoma cells while they autonomously acquired pigmentation in 6–7 days upon low-density (LD) culturing (Fig 1A). We examined levels of IP_3_Rs in the microarrays and found that mRNA expression of IP_3_Rs is augmented with the increase in pigmentation. This observation was further corroborated through quantitative real-time PCR (qRT-PCR) analysis, which demonstrated that the IP_3_R2 isoform exhibited a more substantial increase in expression than the other isoforms (Fig 1B). The second microarray was performed on primary human melanocytes, which were chemically stimulated either with tyrosine, a pigment-inducing agent or phenylthiourea (PTU), a de-pigmentary agent (Fig 1C). In this microarray, only the expression of IP_3_R2 was directly proportional to pigmentation levels. We corroborated this observation by performing qRT-PCRs and found that upon tyrosine-mediated hyperpigmentation IP_3_R2 expression is enhanced (Fig 1D) while upon PTU-driven hypopigmentation IP_3_R2 expression is decreased (Fig 1E). Next, we examined the levels of IP_3_R isoforms in melanocytes using “The Human Protein Atlas,” which shows higher IP_3_R2 expression in comparison to other IP_3_R isoforms (S1A Fig). Collectively, this data along with the microarrays and qRT-PCR validation demonstrate that IP_3_R2 is the most abundant IP_3_R isoform in melanocytes and IP_3_R2 expression is positively associated with pigmentation levels in two independent cellular models.

To investigate the role of the IP_3_R isoforms in melanogenesis, we carried out a targeted small interfering RNA (siRNA) screen against the three IP_3_R isoforms in B16 LD pigmentation model. We first validated the efficacy of siRNAs by performing qRT-PCRs. This analysis revealed a drastic reduction in the mRNA levels of the targeted IP_3_R isoform, while the expression of other isoforms remained unchanged or showed a minor change (S1B–S1D Fig). Next, we examined the effect of IP_3_R isoforms silencing on pigmentation in B16 LD pigmentation model. We observed a phenotypic decrease in the pigmentation upon IP_3_R2 silencing while knockdown of IP_3_R1 and IP_3_R3 showed no phenotypic changes as compared to control siNon-targeting (siNT) condition (Fig 1F). We further validated these phenotypic observations by performing quantitative melanin content assays (Fig 1G).

Since we observed changes in pigmentation upon only IP_3_R2 silencing, we directed our efforts in delineating the role of IP_3_R2 in pigmentation. First of all, we confirmed IP_3_R2 silencing at protein level with both siRNA-mediated transient and shRNA-driven stable knockdown of IP_3_R2 in B16 LD pigmentation model. We observed a significant reduction in IP_3_R2 expression in both these conditions (S1E–S1H Fig). We subsequently analyzed phenotypic and quantitative change in pigmentation upon stable IP_3_R2 silencing in LD pigmentation model. As expected, we found a significant decrease in pigmentation (S1I and S1J Fig). Next, we examined role of IP_3_R2 in αMSH-stimulated physiological pigmentation. We observed a robust decrease in αMSH-induced pigmentation upon both transient (S1K–S1L and S1H–S1I Fig) and stable silencing of IP_3_R2 in B16 cells (S1M–S1P Fig). Finally, we validated critical role of IP_3_R2 in human skin pigmentation by performing experiments in primary human melanocytes. IP_3_R2 silencing with a human IP_3_R2 siRNA led to a significant decrease in both IP_3_R2 protein expression (S1Q and S1R Fig) and the pigmentation phenotype (Fig 1J and 1K) as compared to the control non-targeting siRNA. To substantiate the physiological relevance of IP_3_R2 in pigmentation, we analyzed publicly available microarray datasets to compare IP_3_R2 expression in skin of two distinct ethnic groups: White and African population [23]. We observed that IP_3_R2 expression was higher in the African population compared to the White population (Fig 1L). This unbiased data from an earlier study further validate a positive association between IP_3_R2 expression and pigmentation.

Since IP_3_R2 is a key ER Ca^2+^ release channel, we next examined the effect of IP_3_R2 knockdown on histamine (inducer of Ca^2+^ release via IP_3_Rs) stimulated ER Ca^2+^ release and Thapsigargin (Tg) mediated bulk ER Ca^2+^ mobilization. Our Fura2AM-based live-cell Ca^2+^ imaging experiments show that upon stimulation with histamine the IP_3_R2 knockdown condition exhibited reduced Ca^2+^ release as compared to control siRNA (Fig 1M and 1N). Consequently, the Tg-mediated bulk ER Ca^2+^ mobilization was higher in siIP_3_R2 condition in comparison to control siRNA (Fig 1M and 1O). This indicates that IP_3_R2 plays a functional role in mediating ER Ca^2+^ release in melanocytes. To investigate the significance of IP_3_R2-mediated ER Ca^2+^ release in regulating pigmentation, we utilized a non-conductive pore mutant of IP_3_R2 (IP_3_R2-M), which cannot facilitate Ca^2+^ release and a wild-type functional IP_3_R2 construct [24]. Using quantitative qRT-PCR, we evaluated the expression levels of IP_3_R2 and IP_3_R2-M. Our analysis revealed a significant and specific upregulation of the IP_3_R2 isoform with wild-type IP_3_R2 (S1S Fig) and IP_3_R2-M construct (S1T Fig). Next, we examined the effect of functional IP_3_R2 and IP_3_R2-M overexpression on LD pigmentation. We observed that overexpression of the functional IP_3_R2 isoform resulted in increased pigmentation, while the overexpression of IP_3_R2-M did not enhanced phenotypic pigmentation (Fig 1P). We further quantitated the differences via melanin content assays, which corroborated the qualitative images (Fig 1Q). We next validated these observations by rescuing the functional IP_3_R2 and IP_3_R2-M expression in the stable IP_3_R2 knockdown background. Both the phenotypic and quantitative analysis of pigmentation clearly demonstrated that the overexpression with the functional IP_3_R2 isoform recovered the pigmentation phenotype (Fig 1R and 1S). However, the expression of the IP_3_R2-M failed to enhance the pigmentation phenotype (Fig 1R and 1S). This data highlights that the Ca^2+^ release via IP_3_R2 is required for driving melanin production. Taken together, our data from four independent in vitro model systems (LD pigmentation in B16 cells, αMSH-stimulated physiological melanogenesis, pigmentation in primary human melanocytes and overexpression/rescue experiments) elegantly demonstrate that IP_3_R2 is a positive regulator of pigmentation.

### IP_3_R2 regulates pigmentation in vivo

**Fig 2:**
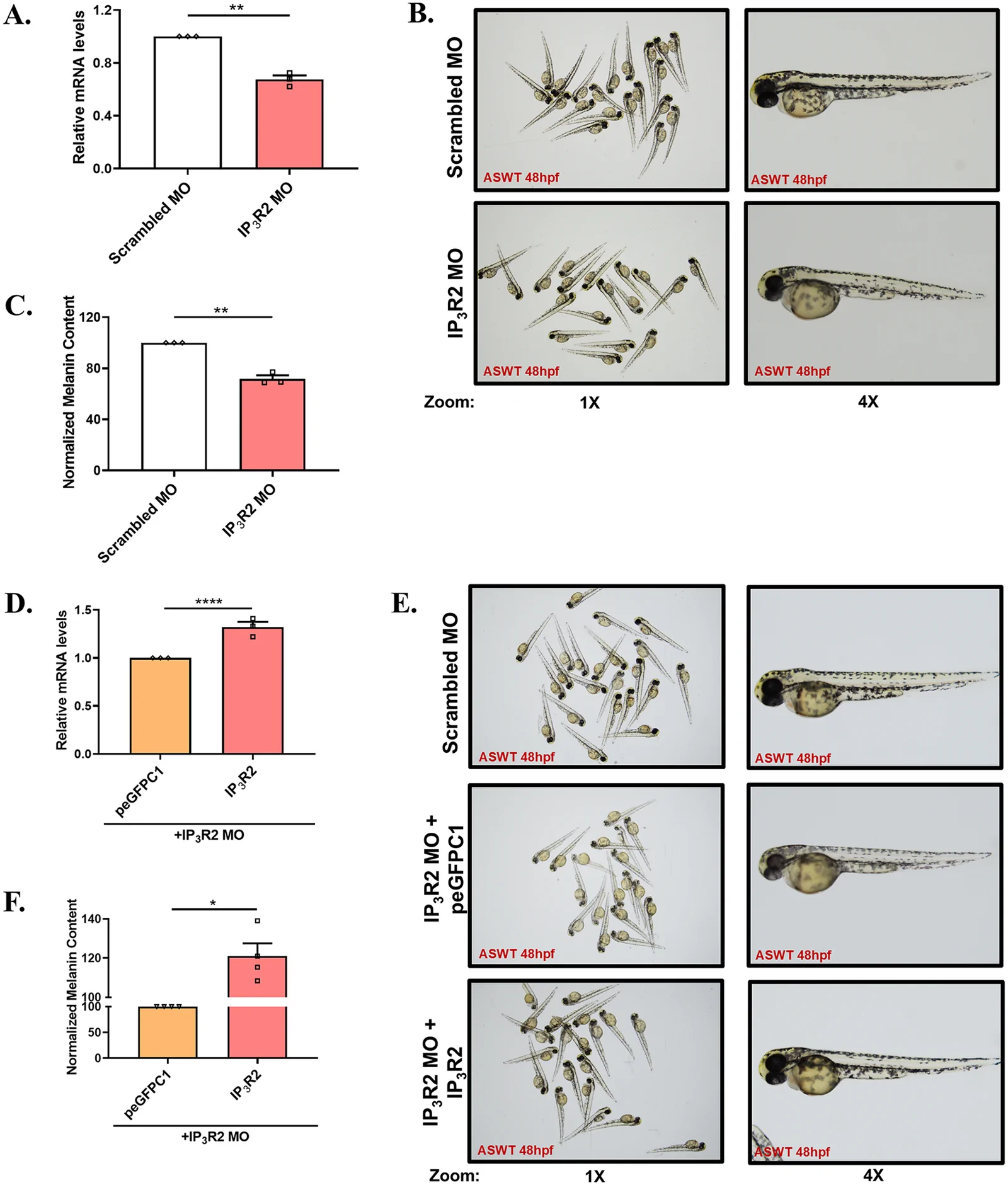
IP_3_R2 drives pigmentation in vivo. **(A)** qRT-PCR analysis showing decrease in IP_3_R2 mRNA expression after 48 h post fertilization (hpf) on zebrafish larvae injected with 400 μM IP_3_R2 morpholino (*N* = 3). **(B)** Representative images of Assam wild type (ASWT) zebrafish after 48hpf injected with 400 μM IP_3_R2 morpholino. **(C)** Bar graph showing melanin content estimation of zebrafish after 48hpf injected with 400 μM IP_3_R2 morpholino (*N* = 3 with 20 embryos in each “N”). **(D)** qRT-PCR analysis showing increase in IP_3_R2 mRNA expression after 48hpf on zebrafish larvae injected with 400 μM IP_3_R2 morpholino and rescued with 120 ng of IP_3_R2 mRNA (*N* = 4). **(E)** Representative images of ASWT zebrafish embryos after 48hpf injected with 400 μM IP_3_R2 morpholino and rescued with 120 ng of IP_3_R2 mRNA. **(F)** Bar graph showing melanin content estimation of zebrafish embryos after 48hpf injected with 400 μM IP_3_R2 morpholino and rescued with 120 ng of IP_3_R2 mRNA (*N* = 3 with 20 embryos in each “N”). Data presented are mean ± SEM. For statistical analysis, one-sample *t* test was performed for panels A, C, D, F using GraphPad Prism software. Here, * *p* < 0.05, ** *p* < 0.01 and **** *p* < 0.0001.

To examine the role of IP_3_R2 in pigmentation in vivo, we performed IP_3_R2 loss-of-function and gain-of-function studies in zebrafish model system. Zebrafish is a well-established model organism for pigmentation studies [17,18,25]. We carried out IP_3_R2 loss-of-function studies by performing IP_3_R2 knockdown by injecting morpholinos targeting IP_3_R2 at single-cell-stage zebrafish embryos. The qRT-PCR analysis demonstrated a significant decrease in IP_3_R2 expression upon IP_3_R2 morpholino injections compared to the scrambled morpholino (Fig 2A). Microscopic examination revealed that IP_3_R2 silencing results in reduction of pigmentation in zebrafish embryos compared to the control group at 48 h post-fertilization (Fig 2B). We further validated the phenotypic observation by performing melanin content assays and observed around 35% reduction in pigmentation upon IP_3_R2 knockdown (Fig 2C).

We next conducted rescue experiments to corroborate the effects observed upon IP_3_R2 silencing. We co-injected either IP_3_R2 or control (GFP) mRNA into zebrafish embryos along with IP_3_R2 morpholinos. qRT-PCR demonstrated that the embryos receiving IP_3_R2 mRNA exhibited significantly higher IP_3_R2 expression levels in comparison to the control group (Fig 2D). Importantly, the embryos rescued with IP_3_R2 mRNA displayed an increase in pigmentation phenotype, which we further substantiated via melanin content assay (Fig 2E and 2F). This data clearly demonstrates that IP_3_R2 regulates pigmentation in vivo. Collectively, our comprehensive in vitro and in vivo studies establish IP_3_R2 as a novel positive regulator of pigmentation.

### IP_3_R2 knockdown enhances stability of melanogenic proteins

To further substantiate the phenotypic observations, we analyzed the mRNA expression of critical melanogenic genes following IP_3_R2 knockdown. We first confirmed IP_3_R2 silencing temporally by performing qRT-PCR (S2A Fig). Subsequently, we assessed the mRNA levels of the melanosomal structural protein (Pre-melanosome Protein 17, i.e., PMEL17 or GP100) and the key melanogenic enzymes (Tyrosinase and DCT). Interestingly, we did not observe significant changes in the mRNA expression of these melanogenic genes (S2B–S2D Fig). We next performed western blotting to investigate the impact of IP_3_R2 knockdown on the protein expression of these melanogenesis regulators. Interestingly, despite the decrease in pigmentation upon IP_3_R2 silencing, we observed a significant increase in the melanogenic proteins (S2E–S2J Fig). Collectively, this suggests that the decrease in pigmentation observed upon IP_3_R2 silencing is not due to transcriptional regulation of melanogenic genes but may involve post-translational mechanisms or alterations in protein stability.

### IP_3_R2 silencing stabilize melanogenic proteins and triggers autophagy

**Fig 3:**
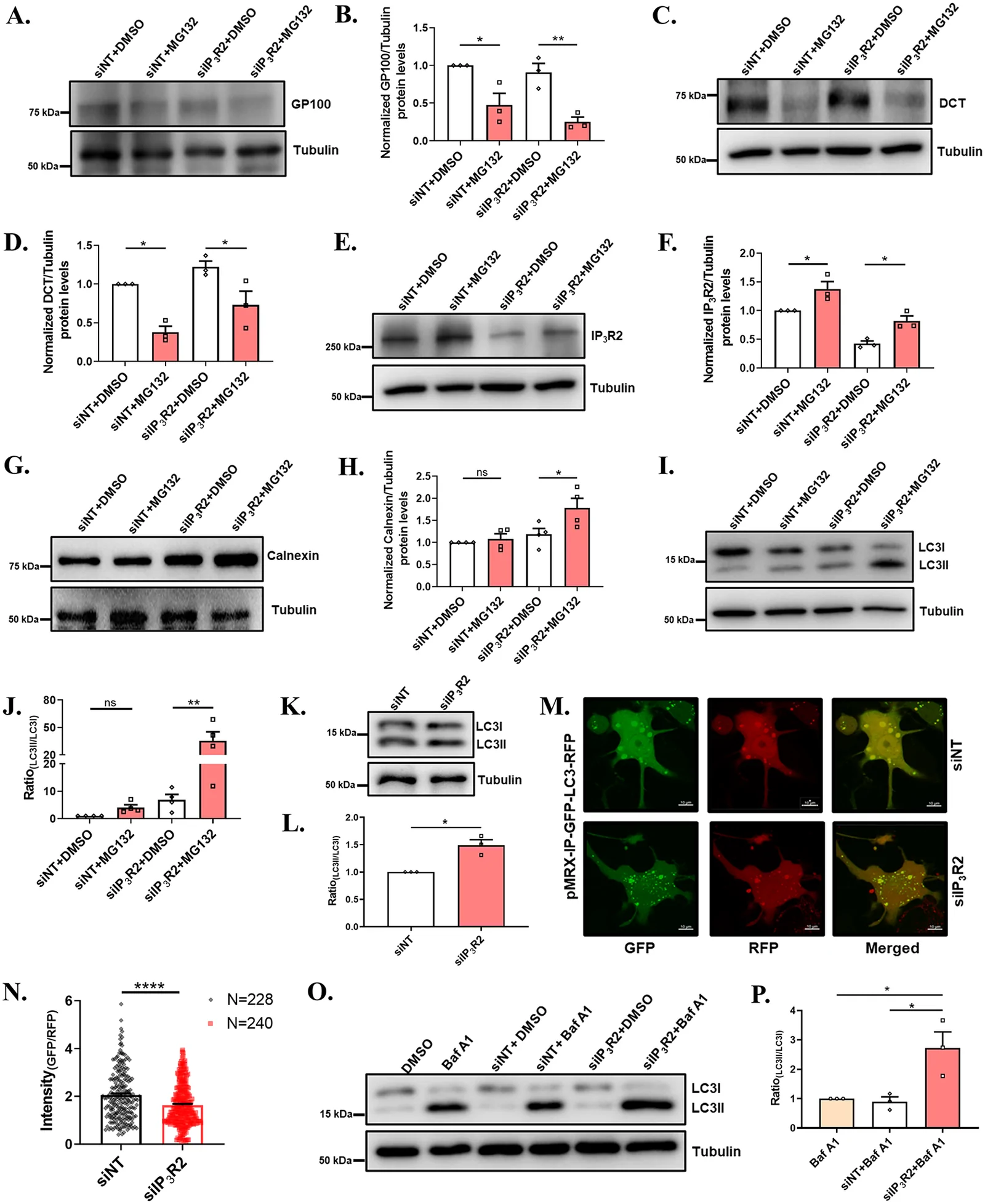
IP_3_R2 silencing stabilize melanogenic proteins and triggers autophagy. **(A)** Representative image of immunoblot showing expression of GP100 in B16 B16 cells transfected with siNT or siIP_3_R2 along with 10 µM MG132 treatment for 6 h. **(B)** Bar graph showing the densitometry of the GP100 band normalized to β-Tubulin (*N* = 3). **(C)** Representative image of immunoblot showing expression of DCT in B16 cells transfected with siNT or siIP_3_R2 along with 10 µM MG132 treatment for 6 **h. (D)** Bar graph showing the densitometry of the DCT band normalized to β-Tubulin (*N* = 3). **(E)** Representative image of immunoblot showing expression of IP_3_R2 in B16 cells transfected with siNT or siIP_3_R2 along with 10 µM MG132 treatment for 6 h. **(F)** Bar graph showing the densitometry of the IP_3_R2 band normalized to β-Tubulin (*N* = 3). **(G)** Representative image of immunoblot showing expression of Calnexin in B16 cells transfected with siNT or siIP_3_R2 along with 10 µM MG132 treatment for 6 h. **(H)** Bar graph showing the densitometry of the Calnexin band normalized to β-Tubulin (*N* = 4). **(I)** Representative image of immunoblot showing expression of LC3II and LC3I in B16 cells transfected with siNT or siIP_3_R2 along with 10 µM MG132 treatment for 6 h. **(J)** Bar graph showing the densitometric analysis of the LC3II/LC3I ratio (*N* = 4). **(K)** Representative image of immunoblot showing expression of LC3II and LC3I in B16 cells after siRNA silencing of IP_3_R2. **(L)** Bar graph showing the densitometric analysis of the LC3II/LC3I ratio (*N* = 3). **(M)** Representative images of confocal imaging in B16 cells after transfected with siNT or siIP_3_R2 along with pMRX-IP-GFP-LC3-RFP probe, scale bar, 10 µm. **(N)** Bar graph shows the quantification of the autolysosome intensity in the GFP:RFP ratio, where ‘*N*’ denotes the number of cells. **(O)** Representative image of immunoblot showing expression of LC3II in B16 cells after siRNA transfection including 6 h of treatment with 100 nM of the lysosomal inhibitor Bafilomycin A1. **(P)** Bar graph showing the densitometric analysis of the LC3II/LC3I ratio (*N* = 3). Data presented are mean ± SEM. For statistical analysis, one-sample *t* test was performed for panel L, an unpaired *t* test was performed for panel N, and Tukey’s multiple comparisons test was performed for panel B, D, F, H, J, P using GraphPad Prism software. Here, ‘ns’ means non-significant; * *p* < 0.05, ** *p* < 0.01, and **** *p* < 0.0001.

We next directed our efforts to determine the underlying mechanism through which IP_3_R2 downregulation increases melanogenic proteins expression. We investigated protein stability of the key melanogenic regulators in presence of protein synthesis inhibitor cycloheximide. Our data show that IP_3_R2 levels are decreased in IP_3_R2-silenced condition (S2K and S2L Fig), while the melanogenic proteins DCT, Tyrosinase and GP100, exhibited higher stability in the IP_3_R2 knockdown condition (S2K and S2M–S2O Fig). Literature suggests that elevated protein stability following synthesis inhibition can reflect reduced protein degradation through multiple pathways, including the ubiquitin-proteasome system (UPS) and lysosomal/autophagic routes [26,27]. To understand which degradation pathway contributes to the altered turnover of melanogenic proteins observed upon IP_3_R2 knockdown, we examined the effects of the proteasome inhibitor, MG132. Our data revealed that the expression of melanogenic proteins GP100 and DCT decreased upon treatment with MG132 compared to the DMSO control (Fig 3A–3D). Importantly, ER-resident proteins IP_3_R2 and calnexin showed increased expression following MG132 treatment (Fig 3E–3H). The contrasting behavior of melanosomal proteins (GP100, DCT) and ER proteins (IP_3_R2, calnexin) under MG132 suggests that GP100 and DCT are not primary proteasomal substrates, and that their steady-state abundance is most likely governed by lysosomal and autophagic mechanisms. Interestingly, proteasome inhibition is well established to activate autophagy as a compensatory degradation mechanism [28–32]. Since, GP100 and DCT are integral membrane proteins of melanosomes, their bulk lysosomal degradation via melanophagy would lead to a decrease in their protein levels. Furthermore, there was a significant rise in LC3II/LC3I levels in IP_3_R2 knockdown plus MG132 treatment condition in comparison to IP_3_R2 knockdown plus DMSO control (fig and 3J), demonstrating robust autophagy induction under these condition. This data is consistent with the established paradigm in which UPS impairment activates autophagy as a compensatory protein quality control mechanism [28–32]. Taken together, these datasets demonstrate that melanogenic proteins exhibit greater stability under IP_3_R2 knockdown conditions. However, we acknowledge that the current data does not provide direct evidence of impaired proteasomal degradation of these proteins. In future, direct proteasome activity measurements and ubiquitination analyses of specific melanosomal substrates would be required to establish this.

Further, to test whether IP_3_R2 knockdown induces autophagy independent of proteasomal inhibition, we examined LC3 lipidation under basal siIP_3_R2 conditions. IP_3_R2 silencing significantly increased the LC3II/LC3I ratio in both the LD pigmentation model (S3A and S3B Fig) and the αMSH-induced pigmentation model (Fig 3K and 3L), indicating elevated LC3II accumulation independent of pharmacological proteasome blockade. These data support a model in which IP_3_R2 knockdown stabilizes melanogenic proteins via reduced proteasomal turnover, with compensatory engagement of autophagy. To substantiate these observations, we utilized the pMRX-IP-GFP-LC3-RFP reporter system, a powerful experimental approach that enables the quantification of autophagic flux by calculating the ratio of GFP to RFP signals [33]. IP_3_R2 silencing significantly reduced GFP/RFP ratio of the GFP-LC3-RFP reporter system in comparison to control conditions thereby indicating enhanced autophagic flux upon IP_3_R2 knockdown (Fig 3M and 3N). We further corroborated this by assessing autophagic flux using Bafilomycin A1, an inhibitor of autophagosome-lysosome fusion. In the presence of Bafilomycin A1, the IP_3_R2 knockdown cells showed a significantly increased LC3-II protein levels suggesting higher accumulation of autophagosomes, i.e., higher autophagy flux, as compared to Bafilomycin A1-treated control cells (Fig 3O and 3P). Taken together, these biochemical and live cell microscopy data clearly demonstrate that IP_3_R2 silencing enhances autophagic flux in melanocytes.

### IP_3_R2 silencing induces melanosome degradation

**Fig 4:**
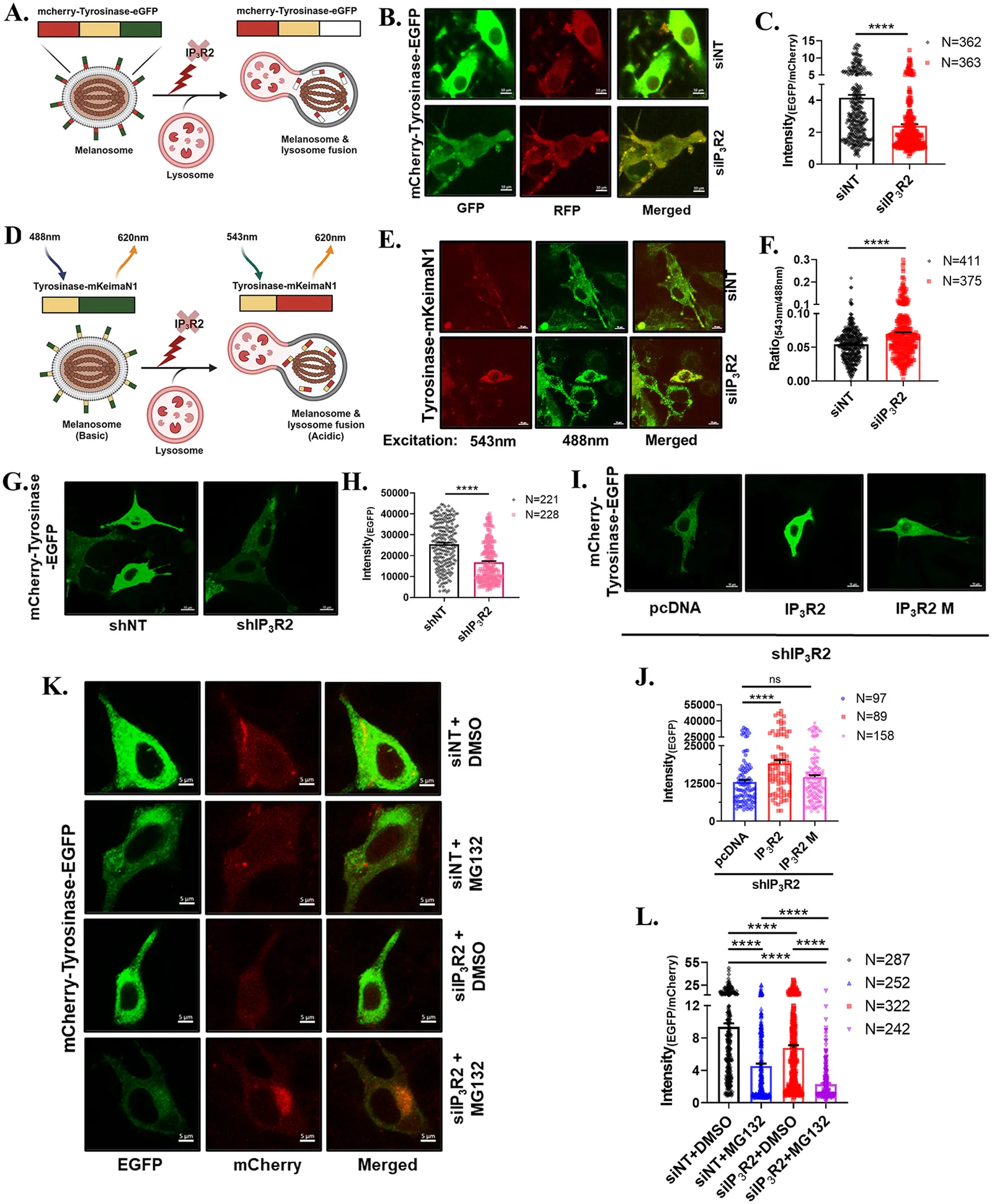
IP_3_R2 silencing enhances melanophagy flux. **(A)** A schematic illustration demonstrating the melanophagy flux upon IP_3_R2 silencing by utilizing a mCherry-Tyrosinase-EGFP construct. Created in BioRender. Motiani, R. (2026) https://BioRender.com/m0tpcom. **(B)** Representative images of confocal imaging in B16 cells after transfected with siNT or siIP_3_R2 along with mCherry-Tyrosinase-EGFP probe, scale bar, 10 µm. **(C)** Bar graph shows the quantification of the autolysosome intensity in the EGFP:mCherry ratio, where ‘*N*’ denotes the number of cells. **(D)** A schematic illustration demonstrating the melanophagy flux upon IP_3_R2 silencing by utilizing Tyrosinase-mKeimaN1 construct. Created in BioRender. Motiani, R. (2026) https://BioRender.com/m0tpcom. **(E)** Representative images of confocal imaging in B16 cells after transfected with siNT or siIP_3_R2 along with Tyrosinase-mKeimaN1 probe, scale bar, 10 µm. **(F)** Bar graph shows the quantification of the autolysosome intensity in the 543 nm:488 nm ratio, which is alternatively excited at 543 nm and 488 nm, where ‘*N*’ denotes the number of cells. **(G)** Representative images of confocal imaging in B16 cells having stable IP_3_R2 knockdown transfected with mCherry-Tyrosinase-EGFP construct, scale bar, 10 µm. **(H)** Bar graph showing the quantification of the EGFP intensity (mCherry inherently present in the shRNA), where ‘*N*’ denotes the number of cells imaged. **(I)** Representative images of confocal imaging in B16 cells having stable IP_3_R2 knockdown transfected with mCherry-Tyrosinase-EGFP construct, scale bar, 10 µm. **(J)** Bar graph showing the quantification of the EGFP intensity (mCherry inherently present in the shRNA), where ‘*N*’ denotes the number of cells imaged. **(K)** Representative images of confocal imaging in B16 cells transfected with mCherry-Tyrosinase-EGFP probe. These cells were also transfected with either siNT or siIP_3_R2 and treated with 10 µM MG132 for 6 h, scale bar, 5 µm. **(L)** Bar graph shows the quantification of the autolysosome intensity in the EGFP:mCherry ratio, where ‘*N*’ denotes the number of cells. Data presented are mean ± SEM. For statistical analysis, an unpaired *t* test was performed for panels C, F, H, Tukey’s multiple comparisons test was performed for panels J and L using GraphPad Prism software. Here, ‘ns’ means non-significant, and **** *p* < 0.0001.

We observed an impaired proteasomal degradation of critical melanogenic proteins localized on melanosomes in the IP_3_R2 knockdown condition. This may increase melanophagy, the selective autophagic degradation of melanosomes, as a cytoprotective mechanism to maintain protein homeostasis [34,35]. Therefore, we developed two de novo ratiometric fluorescent probes to study melanophagy flux in live cells. In the first probe, we tagged Tyrosinase, a protein exclusively localized on melanosomes, with mCherry and EGFP (Fig 4A). The ratiometric, mCherry-Tyrosinase-EGFP probe, functions based on the differential stability of the green and red fluorescent proteins in low-pH milieu. The acidic environment within the lysosome (pH < 5.2) selectively quenches the fluorescent signal of EGFP and has minimal impact on the mCherry signal [36]. We validated the functionality of mCherry-Tyrosinase-EGFP probe by treating B16 cells expressing this probe with rapamycin, a known autophagy inducer, which resulted in a significant decrease in the EGFP signal and a lower EGFP/mCherry ratio than the control condition (S3C and S3D Fig). Conversely, treatment with Bafilomycin A1, an inhibitor of autophagosome-lysosome fusion, led to an increase in the EGFP signal and a higher EGFP/mCherry ratio relative to the control (S3C and S3D Fig). To further characterize the probe, we investigated its localization on melanosomes. To check this, we performed colocalization experiments between the specific melanosome marker PMEL 17/GP100 recognized by anti-HMB45 ab (Human Melanoma Black 45) and the mCherry-Tyrosinase-EGFP probe. We observed a very high Pearson Correlation Coefficient (PCC) and Overlap Coefficient (OC) between HMB45 and mCherry-Tyrosinase-EGFP probe (S3E and S3F Fig). This indicates that the mCherry-Tyrosinase-EGFP localizes to melanosomes. We further validated our probe by employing Retagliptin phosphate (RTG), an inducer of melanophagy [37]. We observed a significant decrease in the EGFP/mCherry ratio upon RTG treatment thereby highlighting the probes’ ability to detect melanosome degradation (S3G and S3H Fig). Collectively, these assays exhibit that mCherry-Tyrosinase-EGFP probe can effectively measure the quantitative changes in melanophagy. We next used this probe to study melanophagy flux upon IP_3_R2 silencing, we observed a significant decrease EGFP signal and a lower EGFP/mCherry ratio in the IP_3_R2 knockdown condition compared to the control non-targeting siRNA condition (Fig 4B and 4C), indicating enhanced melanophagy upon IP_3_R2 silencing.

To further substantiate this observation, we generated an additional fluorescent ratiometric probe using mKeima. mKeima is a pH-sensitive fluorescent protein derived from coral with emission spectra peaking at 620 nm and a biphasic excitation spectra peaking at 543 nm (Red) and 488 nm (Green) in acidic and neutral environments, respectively [38,39]. We developed a ratiometric fluorescent probe by inserting the tyrosinase protein upstream of the mKeimaN1. This fluorescent reporter system enables the monitoring of melanosomal degradation within the acidic lysosomal environment. The signal of the tyrosinase-mKeimaN1 fusion protein increases at 543 nm excitation in response to the low pH conditions resulting in an increased 543/488 nm signal ratio (Fig 4D). The functionality of the tyrosinase-mKeimaN1 probe was assessed by treating tyrosinase-mKeimaN1 transfected B16 cells with rapamycin and Bafilomycin A1. The rapamycin treatment increased red (543 nm excitation) fluorescence signal resulting in a higher 543/488 nm signal ratio while Bafilomycin A1 treatment resulted in a diminished red (543 nm excitation) fluorescence signal and a lower 543/488 nm signal ratio (S3I and S3J Fig). To further characterize the probe, we investigated its localization on melanosomes. To check this, we performed colocalization experiments between the HMB45 and the tyrosinase-mKeimaN1 probe. We observed a very high PCC and OC between HMB45 and the probe (S3K and S3L Fig). This suggests that the tyrosinase-mKeimaN1 probe localize to melanosomes. Collectively, our data elegantly demonstrates that the ratiometric tyrosinase-mKeimaN1 probe can effectively monitor changes in melanophagy. We then used this probe to study melanophagy flux upon IP_3_R2 silencing. We observed a significant increase in the red (543 nm excitation) fluorescence signal and a higher 543/488 nm signal ratio in IP_3_R2 knockdown condition in comparison to the control siRNA group (Fig 4E and 4F) thereby demonstrating that IP_3_R2 silencing leads to an increase in the melanophagy.

To further validate IP_3_R2’s role in melanophagy, we conducted colocalization experiments using the autophagy marker LC3II and the highly specific melanosome marker HMB45. Upon IP_3_R2 silencing, we observed a higher co-localization between HMB45 and LC3II as compared to the siNT control (S4A and S4B Fig). This validates that IP_3_R2 silencing leads to melanosome degradation. To corroborate the microscopic observations, we performed biochemical assays to study melanophagy flux upon IP_3_R2 silencing. We employed Bafilomycin A1 to study the accumulation of melanosomal proteins upon IP_3_R2 knockdown. In the presence of Bafilomycin A1, IP_3_R2-silenced cells exhibited a more pronounced accumulation of melanosomes, as indicated by elevated tyrosinase levels as compared to Bafilomycin A1-treated control siNT condition (S4C and S4D Fig) highlighting that IP_3_R2 knockdown increases melanophagy flux. Next, we carried out ultrastructural studies to substantiate the increase in melanophagy upon IP_3_R2 silencing. Our Transmission Electron Microscopy (TEM) data demonstrates a significant decrease in darkly pigmented stage III or IV melanosome numbers in IP_3_R2-silenced cells in comparison to the control group (S5A and S5B Fig).

Finally, we validated IP_3_R2’s role in melanophagy by performing rescue experiments in cells with stable IP_3_R2 knockdown. We examined melanophagy using mCherry-Tyrosinase-EGFP probe in control cells and in the cells with stable IP_3_R2 knockdown. Since shRNA targeting IP_3_R2 was tagged with RFP, we focused on EGFP fluorescence intensity as readout of decrease in melanophagy (Fig 4G and 4H). Further, we performed rescue experiments with either wild-type IP_3_R2 or IP_3_R2-M and corresponding empty vector control in stable IP_3_R2 knockdown cells (Fig 4I and 4J). Our data show that stable IP_3_R2 knockdown enhances melanophagy (Fig 4G and 4H) which could be rescued with wild-type IP_3_R2 but not IP_3_R2-M (Fig 4I and 4J). To corroborate these observations, we performed ultrastructural studies with the wild-type IP_3_R2 and IP_3_R2-M rescue experiments in stable IP_3_R2 knockdown condition. The TEM data shows a significant increase in melanosome number in cells rescued with IP_3_R2 while rescue with IP_3_R2-M shows similar melanosome number as compared to stable IP_3_R2 knockdown (S5C and S5D Fig). These findings demonstrate that the Ca^2+^ release function of IP_3_R2 is a critical determinant of melanophagy. Subsequently, we investigated whether impairment of proteasomal degradation upon IP_3_R2 silencing alone or together with MG132 selectively triggers melanophagy. To address this, we assessed melanophagy using melanophagy reporter, mCherry-Tyrosinase-EGFP following IP_3_R2 silencing with MG132 treatment. Our observations revealed an increase in melanophagic flux with IP_3_R2 silencing and MG132 treatment compared to siNT with DMSO control (Fig 4K and 4L). This suggests that IP_3_R2 silencing induced inhibition of proteasomal degradation activates melanophagy. Taken together, the confocal imaging with two ratiometric probes, biochemical assays, ultrastructural studies, and the rescue experiments reveal that IP_3_R2 negatively regulates melanophagy.

We next asked that whether the decreased pigmentation phenotype observed in the IP_3_R2 knockdown condition is associated with induction of global organelle macroautophagy or it is specific to melanosome degradation. Therefore, we investigated IP_3_R2 silencing induced macroautophagy of other organelles, i.e., Mitochondria and ER. To explore mitochondrial degradation, we utilized mKeima-Red-Mito-7 fluorescent probe that enables monitoring of mitochondrial degradation (mitophagy), and we used FCCP (carbonyl cyanide-4 (trifluoromethoxy) phenylhydrazone) as a positive control for mitophagy [40,41]. We observed a significant decrease in the red (543 nm excitation) fluorescence signal and a lower 543/488 nm signal ratio of mKeima-Red-Mito-7 in the IP_3_R2 knockdown condition compared to the control siRNA group suggesting a reduction in mitophagy upon IP_3_R2 silencing (S5E and S5F Fig). Conversely, we observed a higher 543/488 nm signal ratio in the FCCP condition compared to the control, indicating an increase in mitophagy (S5E and S5F Fig). Next, to study Reticulophagy/ER-phagy, we utilized GST-Keima-cb5 fluorescent probe that enables monitoring of ER degradation, and we used 4-PBA (4-phenylbutyric acid) as a positive control of reticulophagy [42]. We observed a significant decrease in the red (543 nm excitation) fluorescence signal and a lower 543/488 nm signal ratio in the IP_3_R2 knockdown condition compared to the control siRNA group indicating a decline in ER-phagy (S5G and S5H Fig). As expected, in the 4-PBA condition, we observed a higher 543/488 nm signal ratio as compared to the control, indicating an increase in ER-phagy (S5G and S5H Fig). Therefore, both mitophagy and ER-phagy are decreased upon IP_3_R2 silencing. Collectively, these findings highlight that IP_3_R2 knockdown explicitly induce melanophagy and not general organelle macroautophagy.

### IP_3_R2 knockdown impairs mitochondrial Ca^2+^ uptake and triggers melanophagy

**Fig 5:**
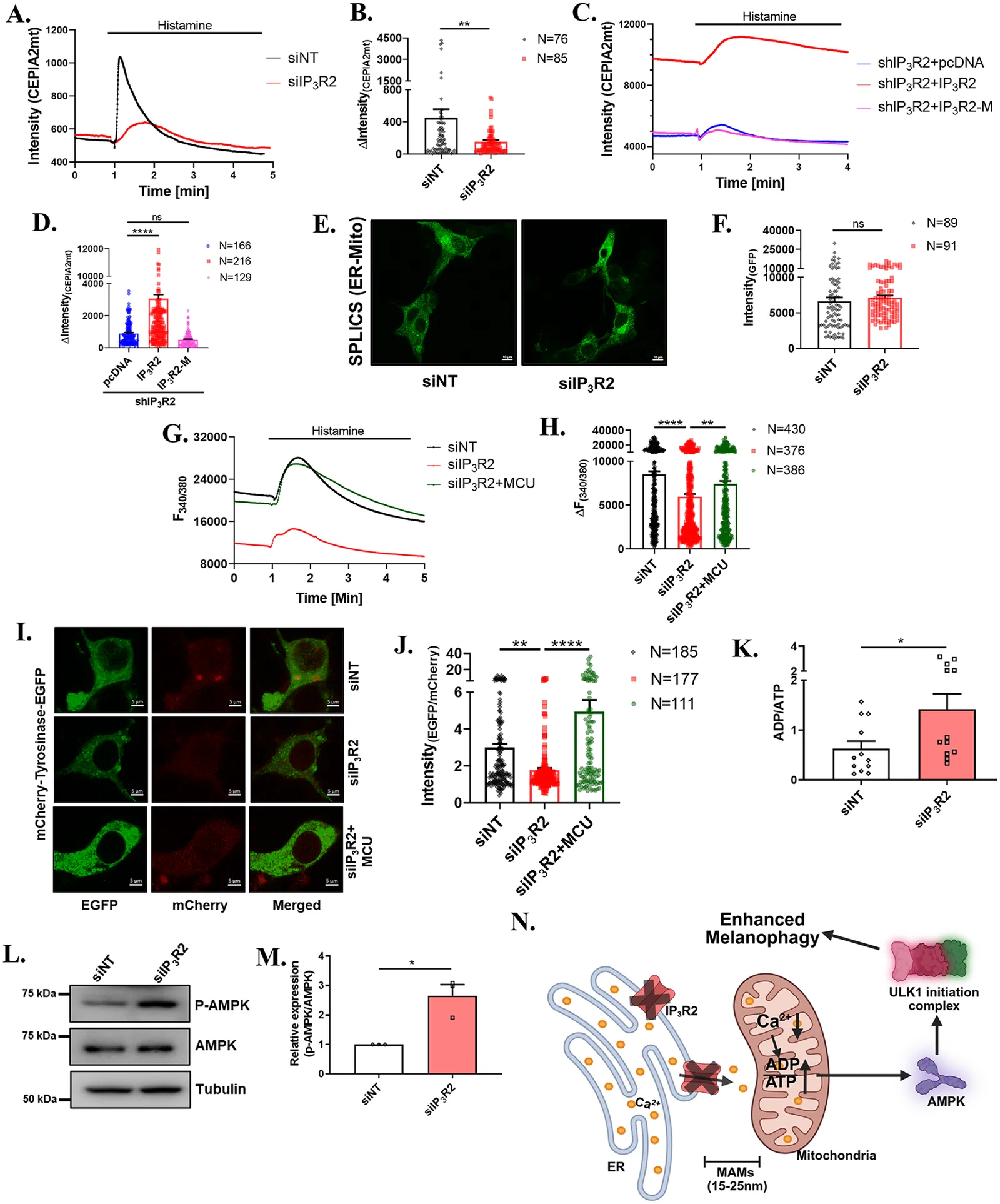
IP_3_R2 silencing reduces mitochondrial Ca^2+^ uptake and triggers melanophagy. **(A)** Representative mitochondrial Ca^2+^ imaging trace using the Cepia2mt probe in B16 cells stimulated with the Histamine after 72 h of siRNA transfection. **(B)** Bar graph showing the quantification of Ca^2+^ imaging traces stimulated with 50 µM histamine, where ‘*N*’ denotes the total number of regions of interest (ROI) in that trace. **(C)** Representative mitochondrial Ca^2+^ imaging trace using the Cepia2mt probe in B16 cells having stable IP_3_R2 knockdown demonstrating rescue with IP_3_R2 and IP_3_R2-M, stimulated with Histamine. **(D)** Bar graph showing the quantification of Ca^2+^ imaging traces stimulated with 50 µM histamine, where ‘*N*’ denotes the total number of ROI in that trace. **(E)** Representative images of confocal imaging in B16 cells after transfected with siNT or siIP_3_R2 along with SPLICS Mt-ER Short P2A construct, scale bar, 10 µm. **(F)** Bar graph showing the quantification of the GFP intensity, where ‘*N*’ denotes the number of cells imaged. **(G)** Representative traces of CEPIA2mt-based Ca^2+^ imaging in B16 cells transfected with either siNT or siIP_3_R2 alone or siIP_3_R2 with MCU overexpression, stimulated with the Histamine. **(H)** Bar graph showing the quantification of Ca^2+^ imaging traces stimulated with 50 µM Histamine, where ‘*N*’ denotes the total number of cells imaged. **(I)** Representative images of confocal imaging in B16 cells transfected with mCherry-Tyrosinase-EGFP probe. These cells were transfected with either siNT or siIP_3_R2 alone or siIP_3_R2 with MCU overexpression, scale bar, 5 µm. **(J)** Bar graph shows the quantification of the autolysosome intensity in the EGFP:mCherry ratio, where ‘*N*’ denotes the number of cells. **(K)** Bar graph shows quantification of the luminescent intensity measures changes in the ADP/ATP ratio (*N* = 12), where ‘*N*’ denotes the different wells imaged. **(L)** Representative image of immunoblot showing expression of p-AMPK and AMPK in B16 cells after siRNA silencing of IP_3_R2. **(M)** Bar graph showing the densitometric analysis of the relative changes in the levels of p-AMPK to total AMPK (*N* = 3). **(N)** A schematic illustration demonstrating that IP_3_R2 silencing augments melanophagy, by reducing mitochondrial Ca^2+^ uptake which subsequently triggers ULK1 complex. Created in BioRender. Motiani, R. (2026) https://BioRender.com/h5bus03. Data presented are mean ± SEM. For statistical analysis, an unpaired *t* test was performed for panels B, F, K, one-sample *t* test was performed for panel M, and Tukey’s multiple comparisons test was performed for panels D, H and J using GraphPad Prism software. Here, ‘ns’ means non-significant; * *p* < 0.05, ** *p* < 0.01, and **** *p* < 0.0001.

To elucidate the underlying mechanism by which IP_3_R2 knockdown triggers melanophagy, we investigated the potential impact of IP_3_R2 silencing on mitochondrial Ca^2+^ dynamics. Mitochondrial Ca^2+^ uptake is a critical determinant of cellular activities [43–45]. Decrease in IP_3_R expression and/or function diminishes the IP_3_R-mediated Ca^2+^ signals thereby hampering mitochondrial function and reducing ATP generation [46]. We measured mitochondrial Ca^2+^ uptake, using the genetically encoded fluorescent Ca^2+^ indicator probe Cepia2mt, in response to histamine (an IP_3_-generating stimuli) in control siNT and siIP_3_R2-transfected cells. We observed that IP_3_R2 knockdown resulted in a substantial decrease in the mitochondrial Ca^2+^ uptake (Fig 5A and 5B). We substantiated these findings by utilizing a B16 cell line with stable IP_3_R2 knockdown and a corresponding control cell line. We assessed histamine-induced mitochondrial Ca^2+^ uptake in these cells and validated that IP_3_R2 depletion leads to reduction in mitochondrial Ca^2+^ influx (S6A and S6B Fig). To further corroborate the role of IP_3_R2 function in regulating mitochondrial Ca^2+^ dynamics, we performed rescue experiments in the stable IP_3_R2 knockdown cells. Either the full-length IP_3_R2 and IP_3_R2-M, were ectopically expressed in the stable IP_3_R2 knockdown cells. Our results show that overexpression of the wild-type IP_3_R2 restored the mitochondrial Ca^2+^ uptake while the IP_3_R2-M failed to rescue the mitochondrial Ca^2+^ uptake in stable IP_3_R2 knockdown cells (Fig 5C and 5D). These findings suggest that the Ca^2+^ release activity of IP_3_R2 plays a critical role in facilitating mitochondrial Ca^2+^ uptake.

To examine the isoform-specific role of IP_3_Rs in regulating mitochondrial Ca^2+^ uptake, we performed independent isoform rescue experiments in stable IP_3_R2 knockdown cells. IP_3_R1, IP_3_R2, or IP_3_R3 were ectopically expressed individually in stable IP_3_R2 knockdown cells. We assessed histamine-induced mitochondrial Ca^2+^ uptake using CEPIA2mt. Our results demonstrate that only re-expression of IP_3_R2, but not IP_3_R1 or IP_3_R3, was able to restore the mitochondrial Ca^2+^ uptake in stable IP_3_R2 knockdown cells (S6C and S6D Fig). These findings establish that IP_3_R2 plays an isoform-specific and non-redundant role in facilitating mitochondrial Ca^2+^ uptake.

We next examined whether the reduced mitochondrial Ca^2+^ uptake observed in IP_3_R2 knockdown cells is solely due to the loss of IP_3_R2 function or if it is accompanied with the changes in the ER and mitochondria proximity. We utilized a split-GFP-based fluorescent reporter system engineered to study ER and mitochondria proximity [24,47]. We observed comparable fluorescence signals in the IP_3_R2 knockdown and control siNT conditions, suggesting no significant alteration in the ER-mitochondria contact sites upon IP_3_R2 silencing (Fig 5E and 5F). Taken together, our data highlights that the decrease in IP_3_R2 activity is the major contributor to the reduced mitochondrial Ca^2+^ uptake.

To determine whether the reduced mitochondrial Ca^2+^ uptake following IP_3_R2 silencing influences melanophagic flux. We enhanced acute mitochondrial Ca^2+^ uptake by overexpressing the mitochondrial Ca^2+^ importer (MCU) in IP_3_R2 knockdown cells. Our findings indicates that MCU overexpression significantly increases mitochondrial Ca^2+^ uptake compared to IP_3_R2 silencing alone (Fig 5G and 5H). We then assessed melanophagy under the same conditions and observed a significant decrease in melanophagic flux upon MCU overexpression in IP_3_R2-silenced cells (Fig 5I and 5J). These findings indicate that mitochondrial Ca^2+^ uptake plays a critical role in regulating melanophagy.

Literature suggests that decrease in mitochondrial Ca^2+^ uptake can impair mitochondrial function and diminish ATP production. This leads to an increase in the ADP/ATP or AMP/ATP ratio, which subsequently triggers activation of the autophagic pathway [46,48–50]. Therefore, we assessed ADP/ATP ratio upon IP_3_R2 knockdown and observed a significant rise in the ADP/ATP ratio (Fig 5K). The increase in the ADP/ATP ratio is a hallmark of energetic stress and it amplifies phosphorylation of energy-sensing enzyme AMP-activated protein kinase (AMPK) [50,51]. We examined the levels of phosphorylated AMPK and witnessed an elevated ratio of phosphorylated AMPK to total AMPK, suggesting that IP_3_R2 silencing enhances AMPK activity (Fig 5L and 5M). AMPK activation triggers macroautophagy through multiple signaling cascades to preserve cellular homeostasis and maintain energy balance. The key signaling modules working downstream of AMPK activation to drive macroautophagy are Unc-51-like kinase1 (ULK1) and mTOR pathway [52–54]. Therefore, we examined the status of these pathways upon IP_3_R2 silencing. Upon western blotting, we observed an increase in the ULK1 phosphorylation in IP_3_R2-silenced cells (S7A and S7B Fig). However, similar analysis revealed that IP_3_R2 silencing did not affect mTOR inactivation, as the phosphorylated mTOR/Total mTOR ratio was unaffected (S7C and S7D Fig). Likewise, mTOR downstream targets, ribosomal protein S6 kinase beta-1 (p70S6K) and eukaryotic translation initiation factor 4E-binding protein 1 (4EBP1) remained unaffected (S7E and S7H Fig). ULK1 phosphorylation induces autophagy by enabling the formation of autophagy initiation complex or ULK complex [55,56]. Taken together, our data suggests that IP_3_R2 silencing augments melanophagy, at least partially, by reducing mitochondrial Ca^2+^ uptake and thereby facilitating autophagy initiation complex formation (Fig 5N).

### IP_3_R2 silencing decreases lysosomal pH

**Fig 6:**
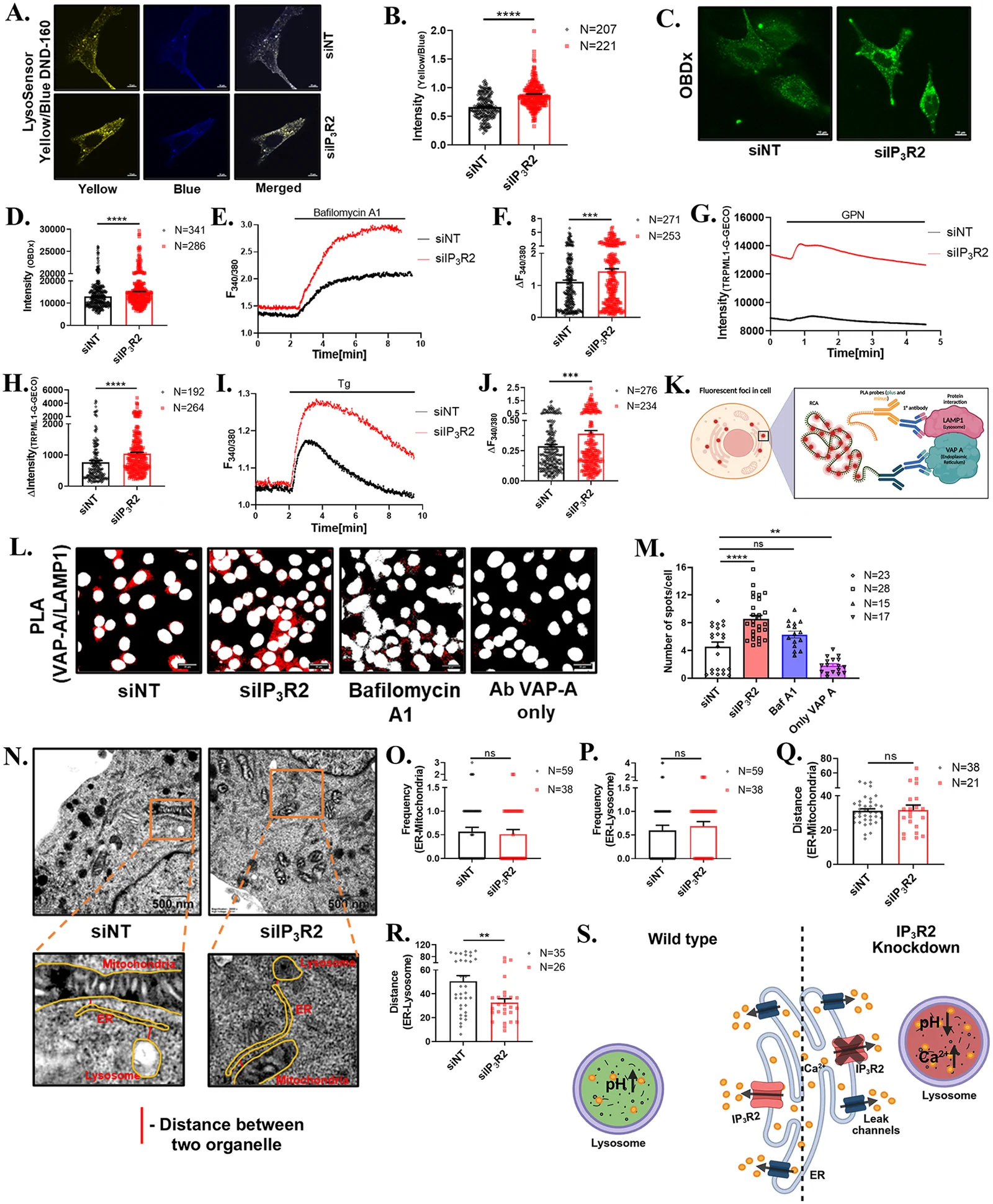
IP_3_R2 silencing decreases lysosomal pH and increases Ca^2+^ content in the lysosome. **(A)** Representative images of confocal imaging in B16 cells loaded with LysoSensor Yellow/Blue DND-160 dye for 5 min after transfected with siNT or siIP_3_R2, scale bar, 10 µm. **(B)** Bar graph showing the quantification of the intraluminal lysosomal pH in Yellow/Blue ratio, where ‘*N*’ denotes the number of cells imaged. **(C)** Representative images of confocal imaging in B16 cells loaded with OG-BAPTA-dextran (OBDx) after transfected with siNT or siIP_3_R2, scale bar, 10 µm. **(D)** Bar graph showing the quantification of the green intensity, where ‘*N*’ denotes the number of cells imaged. **(E)** Representative traces of Fura2AM-based Ca^2+^ imaging in B16 cells stimulated with the BafilomycinA1 after 72 h of siRNA transfection. **(F)** Bar graph showing the quantification of Ca^2+^ imaging traces stimulated with 1 µM BafilomycinA1, where ‘*N*’ denotes the total number of cells imaged. **(G)** Representative Ca^2+^ imaging trace using the TRPML1-G-GECO probe in B16 cells stimulated with the GPN after 72 h of siRNA transfection. **(H)** Bar graph showing the quantification of Ca^2+^ imaging traces stimulated with 300 µM GPN, where ‘*N*’ denotes the total number of ROI in that trace. **(I)** Representative traces of Fura2AM-based Ca^2+^ imaging in B16 cells stimulated with the Tg after 72 h of siRNA transfection. **(J)** Bar graph showing the quantification of Ca^2+^ imaging traces stimulated with 2 µM Tg, where ‘*N*’ denotes the total number of cells imaged. **(K)** A schematic illustration demonstrating the mechanism of PLA by utilizing Primary antibody of LAMP1 and VAP-A. Created in BioRender. Motiani, R. (2026) https://BioRender.com/km2dti6. **(L)** Representative images of confocal imaging in B16 cells performing PLA between LAMP1 and VAP-A proteins after transfected with siNT or siIP_3_R2 or treated with 100 nm Bafilomycin A1 or with Ab VAP-A only, scale bar, 20 µm. **(M)** Bar graph showing the quantification of the number of PLA spots per cell, where ‘*N*’ represents the number of images analyzed in the respective condition. **(N)** Representative TEM image showing B16 cells transfected with siNT or siIP_3_R2, scale bar, 500 nm. **(O)** Bar graph shows the frequency of Mitochondria around ER within 50 nm range, where ‘*N*’ denotes the number of ER. **(P)** Bar graph shows the frequency of Lysosome around ER within 100 nm range, where ‘*N*’ denotes the number of ER. **(Q)** Bar graph shows the distance between ER and Mitochondria within 50 nm range, where ‘*N*’ denotes the number of distances measured between organelles. **(R)** Bar graph shows the distance between ER and Lysosomes within 100 nm range, where ‘*N*’ denotes the number of distances measured between organelles. **(S)** A schematic illustration demonstrating that IP_3_R2 silencing decreases pH and increases lysosomal Ca^2+^ content. Created in BioRender. Motiani, R. (2026) https://BioRender.com/h5bus03. Data presented are mean ± SEM. For statistical analysis, an unpaired *t* test was performed for panels B, D, F, H, J, O, P, Q, R and Dunnett’s multiple comparisons test was performed for panel M using GraphPad Prism software. Here, ‘ns’ means non-significant; ** *p* < 0.01, *** *p* < 0.001 and **** *p* < 0.0001.

Since lysosomes play a crucial role in macroautophagy, we investigated potential impact of IP_3_R2 knockdown on lysosome biology. Lysosomal pH is a critical regulator of the autophagic cargo degradation as it controls activity of degradative enzymatic within lysosomes [57]. We estimated lysosomal pH using a ratiometric pH-sensitive fluorescent probe Lysosensor Yellow/Blue. We observed that IP_3_R2 silencing results in a significant decrease in lysosomal pH, as evident from elevated Yellow/Blue ratio (Fig 6A and 6B). To further validate this data, we utilized LAMP1-RpHLuorin2 ratiometric sensor to measure lysosomal pH. Our results show that IP_3_R2 silencing leads to a significant decrease in lysosomal pH, as indicated by a lower 405/488 nm fluorescence ratio in the IP_3_R2 knockdown condition as compared to siNT control condition (S8A and S8B Fig). The data from two independent ratiometric probes demonstrates that IP_3_R2 silencing decreases lysosomal pH and that leads to enhanced melanophagy thereby reducing pigmentation. We further validated positive association between lysosomal pH and pigmentation levels by utilizing primary human melanocytes from two distinct origins, i.e., White (lightly pigmented) and Afro-American (darkly pigmented). We observed that lightly pigmented primary human melanocytes exhibit a substantially lower lysosomal pH, as indicated by an increased Yellow/Blue ratio of Lysosensor Yellow/Blue, in comparison to darkly pigmented primary human melanocytes (S8C and S8D Fig). We were intrigued by the increase in lysosomal acidity following the IP_3_R2 silencing. The V-ATPase (Vacuolar-type ATPase)/H^+^-ATPase is essential for lysosomal acidification by actively transporting H^+^ ions (protons) into the lysosome, which lowers its pH [58,59]. Hence, we examined the expression of genes responsible for the formation of V-ATPase subunits. Our results reveal that the IP_3_R2 silencing leads to an increase in expression of ATP6V0D1 and ATP6VIH compared to siNT control, thereby most likely promoting a greater influx of H^+^ ions into the lysosome (S8E and S8F Fig). Taken together, our data show that IP_3_R2 silencing reduces lysosomal pH, which is associated with lower pigmentation levels.

### IP_3_R2 silencing enhances lysosomal Ca^2+^ content

Given the crucial role of Ca^2+^ in modulating lysosomal function [60,61], we next examined the effect of IP_3_R2 knockdown on lysosomal Ca^2+^ levels. Earlier studies have shown that lysosomes accumulate Ca^2+^ released by all IP_3_ receptor subtypes, and blocking these receptors leads to lysosomal dysfunction and lysosomal storage disorders (LSD)-like phenotype [62,63]. Therefore, we expected that silencing the IP_3_R2 channel would reduce lysosomal Ca^2+^ levels. However, to our surprise, we observed that IP_3_R2 silencing increased Ca^2+^ content within the lysosomes in comparison to control condition, as measured with Oregon Green 488 BAPTA-1 dextran (OBDx) (Fig 6C and 6D). To further substantiate these findings, we used an indirect approach to evaluate lysosomal Ca^2+^ levels using Fura2-AM. We used an established protocol of releasing lysosomal Ca^2+^ into the cytosol by treating cells with Bafilomycin A1. We observed that lysosomal Ca^2+^ release is higher in IP_3_R2-silenced cells compared to the siNT-transfected control cells (Fig 6E and 6F). Moreover, we employed Glycyl-L-phenylalanine 2-naphthylamide (GPN) to disrupt the lysosomal membrane integrity and then measured the resultant Ca^2+^ elevation at the lysosomal periphery using the TRPML1-G-GECO Ca^2+^ sensor. This analysis also revealed a significantly greater Ca^2+^ release in the IP_3_R2 knockdown condition compared to the control (Fig 6G and 6H). Taken together, our live-cell Ca^2+^ imaging data from three independent experimental setups clearly demonstrate that IP_3_R2 silencing results in higher lysosomal Ca^2+^ accumulation. This further suggests that the higher lysosomal Ca^2+^ levels are associated with enhanced melanophagy and lower pigmentation levels. Consistent with these findings, we found that lightly pigmented primary human melanocytes have significantly elevated lysosomal Ca^2+^ content compared to darkly pigmented primary human melanocytes (S8G and S8H Fig). To eliminate the possible contribution of ER Ca^2+^ release during measurement of Ca^2+^ efflux from acidic compartment, we first treated cells with Tg to deplete ER Ca^2+^ stores and followed by either Bafilomycin A1 or GPN to trigger Ca^2+^ release solely from the acidic compartment. Notably, even after Tg treatment IP_3_R2 silencing resulted in greater Ca^2+^ efflux compared to siNT control, upon induction with Bafilomycin A1 or GPN (S8I, S8K, S8L and S8N Fig). Additionally, in line with data presented in Fig 1O, IP_3_R2 knockdown led to an increase in Ca^2+^ release from ER leak channels upon Tg treatment (S8I, S8J, S8L, and S8M Fig). Taken together, our data demonstrates that IP_3_R2 silencing results in higher Ca^2+^ release from acidic compartments.

The above findings prompted an investigation into the mechanism by which lysosomes accumulate higher Ca^2+^ upon IP_3_R2 silencing. Recent studies have demonstrated that TMBIM6, a leak channel on the ER membrane, facilitates Ca^2+^ uptake into lysosomes [64] and lysosomal transmembrane protein 165 (TMEM165) acts as a Ca^2+^ entry channel thereby bringing Ca^2+^ into lysosomes [65]. Therefore, we measured Ca^2+^ release from ER leak channels using cytosolic Ca^2+^ indicator Fura2 AM. We treated B16 cells with thapsigargin, which inhibits the SERCA pump and we examined ER Ca^2+^ leak into cytosol. We observed an increase in ER Ca^2+^ leak in the IP_3_R2 knockdown cells compared to the non-targeting control (Fig 6I and 6J). Further, our qRT-PCR analysis revealed that TMEM165 expression is significantly elevated in IP_3_R2 silencing condition compared to the control (S9A Fig). To investigate the involvement of TMEM165 in lysosomal Ca^2+^ influx triggered by IP_3_R2 silencing, we silenced TMEM165 using siRNA. qRT-PCR analysis demonstrates decrease in TMEM165 expression upon TMEM165 silencing (S9B Fig). Thereafter, we measured lysosomal Ca^2+^ using OBDx and observed reduced Ca^2+^ in the lysosomal lumen upon TMEM165 silencing (S9C and S9D Fig). Importantly, along with reduced lysosomal Ca^2+^, TMEM165 knockdown resulted in an increase in pigmentation (S9E and S9F Fig). To understand the increase in pigmentation upon TMEM165 silencing, we assessed melanophagy utilizing mCherry-Tyrosinase-EGFP probe. Our analysis revealed that TMEM165 silencing results in reduced melanophagy (S9G and S9H Fig). This suggests that increased pigmentation upon TMEM165 knockdown is at least partially due to the decrease in melanophagy flux. Next, we examined the role of TMEM165 in IP_3_R2 silencing induced increase in lysosomal Ca^2+^ levels by co-transfecting IP_3_R2 and TMEM165 siRNAs. We compared lysosomal Ca^2+^ content among control siNT, siIP_3_R2 only and siIP_3_R2+siTMEM165 conditions. This analysis demonstrated that IP_3_R2 knockdown-driven rise in lysosomal Ca^2+^ is significantly reduced upon co-silencing of TMEM165 (S9I and S9J Fig). These results suggest that the loss of IP_3_R2 leads to an increase in lysosomal Ca^2+^ uptake via lysosomal Ca^2+^ importer channel TMEM165. We next examined the impact of reduced lysosomal Ca^2+^ uptake following the co-silencing of IP_3_R2 and TMEM165 on melanophagy. We employed mCherry-Tyrosinase-EGFP melanophagy probe and observed decreased melanophagy upon co-knockdown of IP_3_R2 and TMEM165 compared to the control conditions (S9K and S9L Fig). To further validate the role of TMEM165 in lysosomal Ca^2+^ regulation and melanophagy, we performed complementary gain-of-function experiments by overexpressing TMEM165 in B16 cells. We first assessed lysosomal Ca^2+^ content using OBDx and observed a significant increase in lysosomal Ca^2+^ levels upon TMEM165 overexpression compared to the pcDNA control (S9M and S9N Fig). We next examined whether the TMEM165 overexpression-driven increase in lysosomal Ca^2+^ has any impact on melanophagy flux utilizing the mCherry-Tyrosinase-EGFP melanophagy probe. Our analysis revealed that TMEM165 overexpression results in enhanced melanophagy flux, as evidenced by a significant reduction in the EGFP/mCherry ratio compared to pcDNA control cells (S9O and S9P Fig). These gain-of-function results are consistent with and complementary to the TMEM165 silencing data, together establishing that TMEM165-mediated lysosomal Ca^2+^ uptake is a critical determinant of melanophagy flux. Collectively, this demonstrates that lysosomal Ca^2+^ influx via TMEM165 channel is crucial for driving melanophagy upon IP_3_R2 silencing.

To investigate if proximity between ER and lysosomes influences Ca^2+^ transfer from ER to lysosome upon IP_3_R2 silencing, we employed in situ proximity ligation assays (PLA). This technique allows to determine if an ER protein, VAP-A and a lysosomal protein, LAMP1, are located within ~40 nm of each other, indicating close spatial interactions [63] (Fig 6K). The PLA data shows an increase in PLA spots in IP_3_R2-silenced condition compared to the control condition (Fig 6L and 6M). However, this increase is not observed when cells are treated with Bafilomycin A1, specific inhibitor of H^+^-ATPase that inhibits acidification, or when one primary antibody was omitted, confirming the specificity of the PLA (Fig 6L and 6M), suggesting that IP_3_R2 downregulation increases ER and lysosomal interactions. We next performed ultrastructural studies to further examine the distance between organelles upon IP_3_R2 silencing. Our Transmission Electron Microscopy (TEM) data demonstrates no change in the frequency between ER-Mitochondria and ER-Lysosome contacts (Fig 6N–6P). Additionally, we found no change in distance between ER-Mitochondria but there was a significant decrease in ER-Lysosome distance in siIP_3_R2 condition compared to siNT control (Fig 6N, 6Q, and 6R). This confirms that IP_3_R2 silencing does not affect the contact frequency between organelles or the proximity between ER-Mitochondria, but it significantly increase the ER-Lysosome proximity.

Taken together, our data demonstrate that IP_3_R2 silencing leads to elevated Ca^2+^ levels within ER. This subsequently enhances Ca^2+^ release through leak ER channels and increases Ca^2+^ influx into lysosomes via TMEM165. Moreover, our data show that IP_3_R2 knockdown augments ER and lysosomal proximity thereby contributing to increased lysosomal Ca^2+^ levels. Collectively, these results reveal that the loss of IP_3_R2 results in a substantial increase in lysosomal Ca^2+^ level, which may serve as a critical upstream signal that triggers melanophagy (Fig 6S).

### Interplay between IP_3_R2 and TRPML1 drives lysosomal Ca^2+^ release and melanophagy

**Fig 7:**
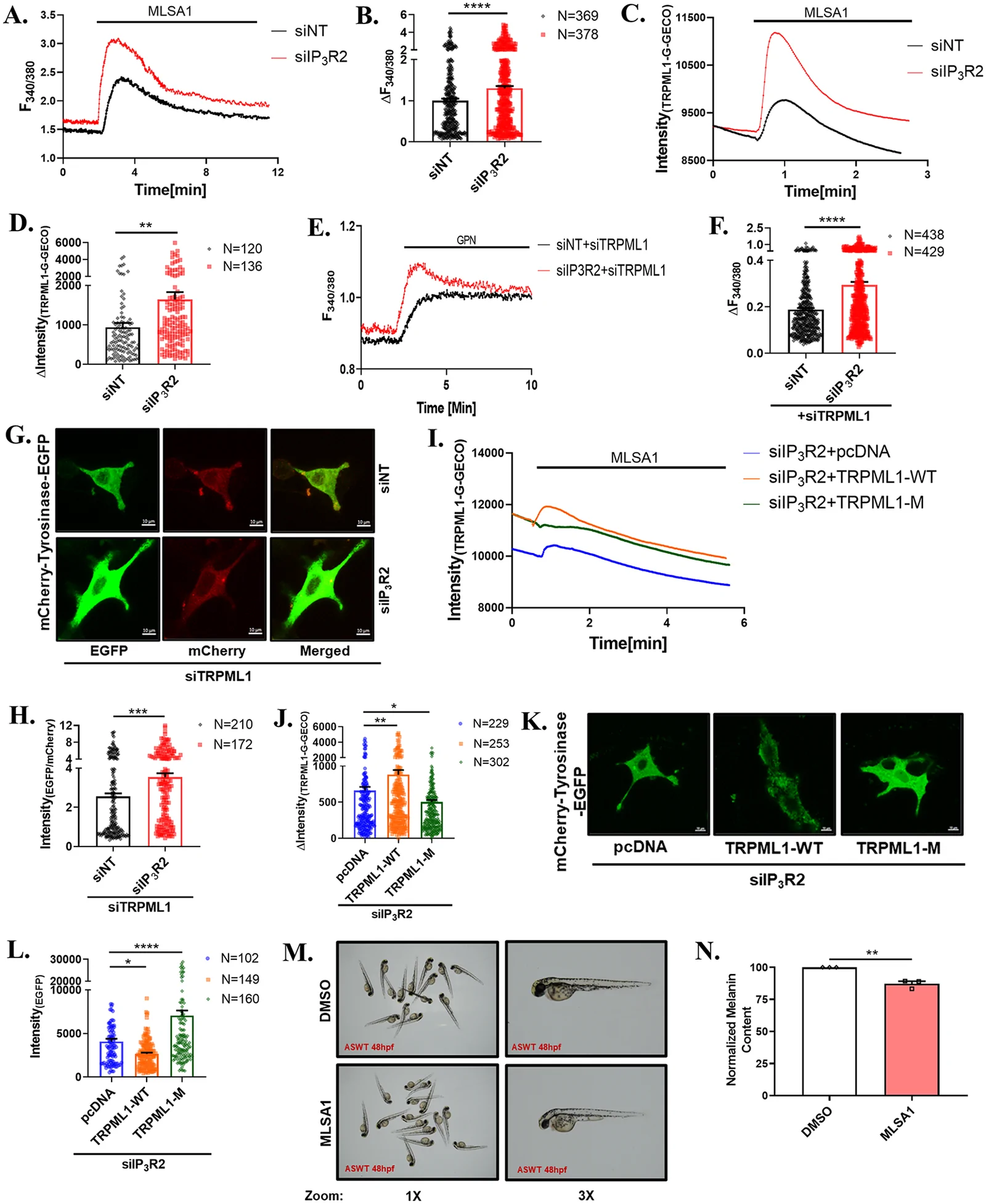
IP_3_R2 knockdown augments TRPML1 activity and enhances melanophagy flux. **(A)** Representative traces of Fura2AM-based Ca^2+^ imaging in B16 cells stimulated with the MLSA1 after 72 h of siRNA transfection. **(B)** Bar graph showing the quantification of Ca^2+^ imaging traces stimulated with 20 µM MLSA1, where ‘*N*’ denotes the total number of cells in that trace. **(C)** Representative Ca^2+^ imaging trace using the TRPML1-G-GECO probe in B16 cells stimulated with the MLSA1 after 72 h of siRNA transfection. **(D)** Bar graph showing the quantification of Ca^2+^ imaging traces stimulated with 20 µM MLSA1, where ‘*N*’ denotes the total number of ROI in that trace. **(E)** Representative traces of Fura2AM-based Ca^2+^ imaging in B16 cells stimulated with the GPN after 72 h of siRNA-mediated dual transfections of IP_3_R2 and TRPML1. **(F)** Bar graph showing the quantification of Ca^2+^ imaging traces stimulated with 300 µM GPN, where ‘*N*’ denotes the total number of cells in that trace. **(G)** Representative images of confocal imaging in B16 cells after co-transfected with siIP_3_R2 and siTRPML1 along with mCherry-Tyrosinase-EGFP probe, scale bar, 10 µm. **(H)** Bar graph shows the quantification of the autolysosome intensity in the EGFP:mCherry ratio, where ‘*N*’ denotes the number of cells. **(I)** Representative trace of lysosomal Ca^2+^ imaging using the TRPML1-G-GECO probe in B16 cells transfected with siIP_3_R2 demonstrating rescue with TRPML1-WT and TRPML1-M. **(J)** Bar graph showing the quantification of Ca^2+^ imaging traces stimulated with 20 µM MLSA1, where ‘*N*’ denotes the total number of ROI in that trace. **(K)** Representative images of confocal imaging using the mCherry-Tyrosinase-EGFP construct in B16 cells transfected with siIP_3_R2 demonstrating rescue with TRPML1-WT and TRPML1-M, scale bar, 10 µm. **(L)** Bar graph showing the quantification of the EGFP intensity (mCherry inherently present in the TRPML1 plasmid), where ‘*N*’ denotes the number of cells imaged. **(M)** Representative images of Assam wild type (ASWT) zebrafish at 48hpf treated with either MLSA1 or vehicle control. **(N)** Bar graph showing melanin content estimation of zebrafish embryo after 48hpf (*N* = 3 with 20 embryos in each “N”. Data presented are mean ± SEM. For statistical analysis, unpaired *t* test was performed for panels B, D, F, H, and Dunnett’s multiple comparisons test was performed for panels J, L using GraphPad Prism software. Here, * *p* < 0.05, ** *p* < 0.01, *** *p* < 0.001 and **** *p* < 0.0001.

Recent studies have reported that lysosomal Ca^2+^ efflux via TRPML channels triggers autophagy [66,67]. Moreover, literature suggests that the acidic environment within the lysosome stimulates TRPML1 activity [68–70]. Since we observed an increase in lysosomal Ca^2+^ levels and lower lysosomal pH upon IP_3_R2 silencing, we investigated role of TRPML channels in driving melanophagy. Our FURA2-AM-based cytosolic Ca^2+^ measurements revealed that stimulation with TRPML agonist MLSA1 results in higher lysosomal Ca^2+^ release in IP_3_R2 knockdown condition compared to siNT control (Fig 7A and 7B). We next corroborated role of TRPML1 in enhanced lysosomal Ca^2+^ release by using a genetically encoded Ca^2+^ sensor TRPML1-G-GECO that measures Ca^2+^ levels in TRPML1 proximity [71]. The live-cell Ca^2+^ imaging with TRPML1-G-GECO demonstrated that MLSA1 stimulation results in higher Ca^2+^ release from TRPML1 in IP_3_R2 knockdown condition (Fig 7C and 7D). This data suggests that IP_3_R2 downregulation augments TRPML1 activity.

We then carried out TRPML1 loss-of-function studies. We initially conducted qRT-PCR analysis to evaluate the specificity of siTRPML1 across different TRPML isoforms. This analysis demonstrated that siRNA-mediated TRPML1 knockdown is both robust and specific (S10A Fig). Using the TRPML1-G-GECO Ca^2+^ sensor, we measured MLSA1-stimulated Ca^2+^ release from TRPML1 and observed almost complete abrogation of TRPML1-mediated lysosomal Ca^2+^ release in siTRPML1 condition (S10B and S10C Fig). This data demonstrates that the siTRPML1 efficiently and specifically decreases TRPML1 expression and activity. We next validated TRPML1’s role in enhanced lysosomal Ca^2+^ release upon IP_3_R2 knockdown by co-silencing IP_3_R2 and TRPML1. Since lysosomal Ca^2+^ release experiments with MLSA1 are not ideal upon TRPML1 knockdown, we assessed lysosomal Ca^2+^ store content using GPN in cells co-transfected with siIP_3_R2 and siTRPML1 and used siNT and siTRPML1 co-transfected cells as control. Our results demonstrate that combined knockdown of IP_3_R2 and TRPML1 leads to a significant increase in GPN-induced Ca^2+^ elevation compared to the control condition (Fig 7E and 7F), indicating a greater accumulation of Ca^2+^ within the lysosomal lumen upon co-silencing. Collectively, this data demonstrates that enhanced lysosomal Ca^2+^ release observed upon IP_3_R2 knockdown is dependent on TRPML1.

We next examined role of TRPML1 in regulating lysosomal pH. Our analysis using LysoSensor Yellow/Blue revealed that TRPML1 knockdown decreases the Yellow/Blue ratio, suggesting an increase in lysosomal pH (S10D and S10E Fig). Furthermore, we assessed TRPML1’s role in melanophagy using mCherry-Tyrosinase-EGFP probe. TRPML1 knockdown did not change melanophagy flux compared to siNT control (S10F and S10G Fig). Therefore, our data suggests that TRPML1 knockdown alone does not impact melanophagy. We then investigated if TRPML1 contributes to the increase in melanophagy observed upon IP_3_R2 knockdown. We co-silenced IP_3_R2 and TRPML1 and assessed melanophagy with mCherry-Tyrosinase-EGFP probe. Our results show that the combined IP_3_R2 and TRPML1 knockdown leads to a reduction in melanophagy in comparison to the siNT + siTRPML1 control condition (Fig 7G and 7H). To further validate role of TRPML1 in IP_3_R2 silencing-induced melanophagy, we performed rescue experiments with either wild-type TRPML1 or TRPML1 non-conducting pore mutant (D471K/D472K) that cannot facilitate Ca^2+^ release from lysosomes [72]. Firstly, we corroborated the functionality of wild-type TRPML1 and pore-dead TRPML1 mutant (TRPML1-M) by measuring TRPML1-mediated lysosomal Ca^2+^ release with TRPML1-G-GECO. Our results show that overexpression of the wild-type TRPML1 leads to an increase in Ca^2+^ release compared to the control condition, while the pore-dead TRPML1 mutant exhibited a decrease in Ca^2+^ release relative to the control (S10H and S10I Fig). Next, we examined if TRPML1 contributes to enhanced lysosomal Ca^2+^ release observed upon IP_3_R2 knockdown. We ectopically expressed either wild-type TRPML1 or TRPML1-M in IP_3_R2-silenced cells and measured Ca^2+^ release using TRPML1-G-GECO. Our data show that wild-type TRPML1 augments lysosomal Ca^2+^ release while TRPML1-M decreases the Ca^2+^ release in comparison to vector control pcDNA in IP_3_R2-silenced cells (Fig 7I and 7J). This data corroborates that TRPML1-mediated Ca^2+^ release contributes to enhanced lysosomal Ca^2+^ release observed upon IP_3_R2 silencing. We then investigated role of TRPML1 in driving melanophagy upon IP_3_R2 knockdown. We assessed melanophagy levels using the mCherry-Tyrosinase-EGFP probe. Our confocal analysis demonstrate that in IP_3_R2 silenced cells, overexpression of wild-type TRPML1 further enhanced melanophagy while ectopic expression of TRPML1-M led to a significant decrease in melanophagy (Fig 7K and 7L). Finally, we validated TRPML1’s role in pigmentation in vivo using zebrafish model system. The addition of MLSA1 (TRPML activator) resulted in a noticeable reduction in pigmentation phenotype when compared to the vehicle control (Fig 7M). This phenotypic decrease was further validated through melanin content assays, which confirmed a reduction in pigmentation following MLSA1 treatment (Fig 7M and 7N). Collectively, these results clearly establish that downstream of IP_3_R2 knockdown, TRPML1-mediated lysosomal Ca^2+^ release plays a critical role in driving melanophagy and reducing pigmentation.

### TRPML1-mediated lysosomal Ca^2+^ release regulates melanophagy via TFEB translocation

**Fig 8:**
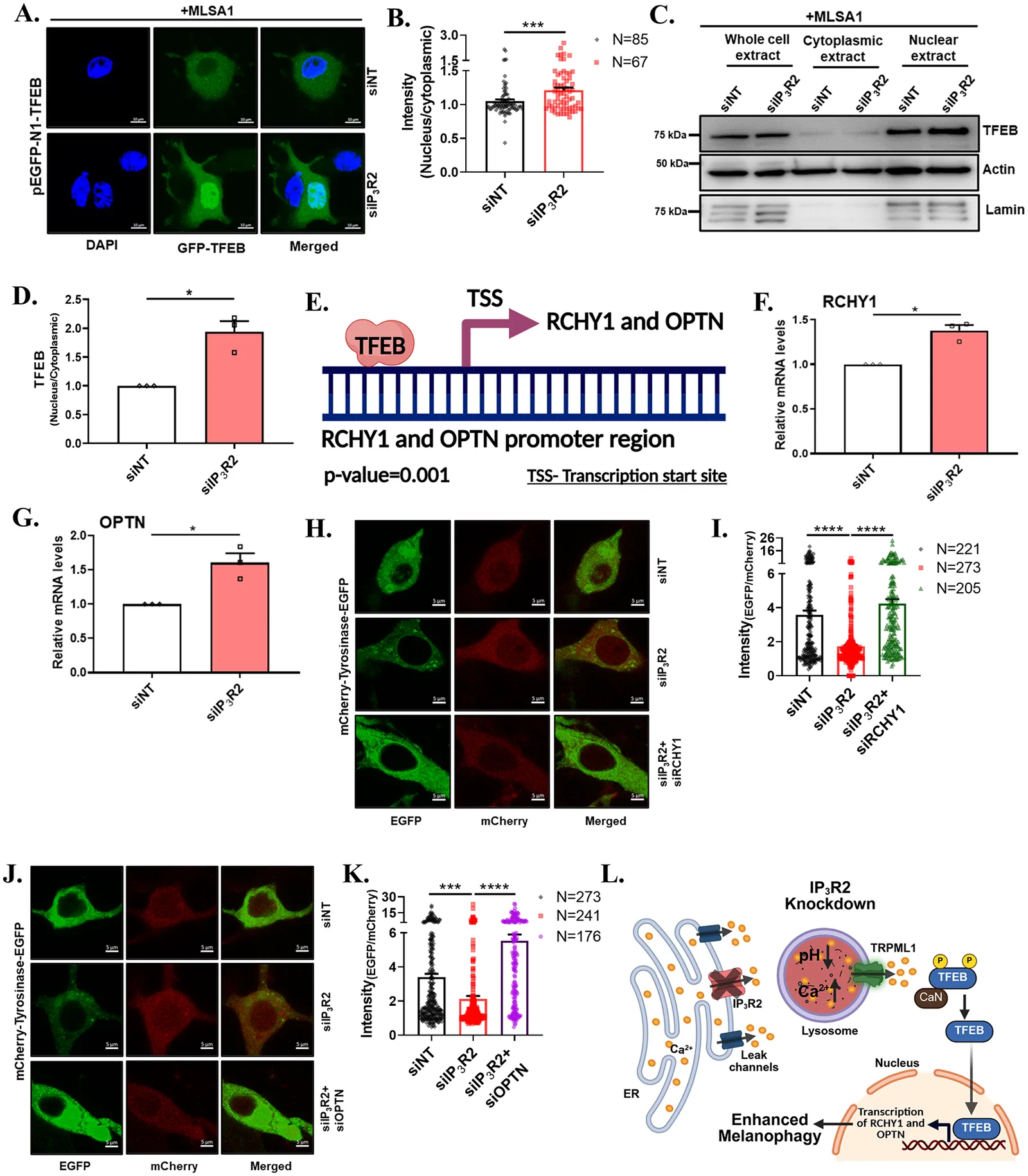
TRPML1-mediated lysosomal Ca^2+^ release regulates melanophagy via TFEB nuclear translocation. **(A)** Representative images of confocal imaging in B16 cells demonstrates TFEB nuclear translocation stimulated with 20 µM MLSA1 for 1 h after transfecting with siNT or siIP_3_R2 along with pEGFP-N1-TFEB construct, scale bar, 10 µm. **(B)** Bar graph showing the quantification of the GFP intensity from the cytoplasm and nucleus in the nucleus/cytoplasmic ratio following the addition of 20 µM MLSA1 for 1 h, where ‘*N*’ denotes the number of cells imaged. **(C)** Representative image of immunoblot showing expression of TFEB in B16 cells from the cytoplasm and nucleus following subcellular fractionation after siRNA silencing of IP_3_R2. **(D)** Bar graph showing the densitometric analysis of the relative changes in the levels of TFEB by quantitating nucleus/cytoplasmic ratio (*N* = 3). **(E)** A schematic illustration demonstrating the transcriptional regulation of the TFEB on expression of genes RCHY1 and OPTN. Created in BioRender. Motiani, R. (2026) https://BioRender.com/wu3kyes. **(F)** qRT-PCR analysis showing increase in RCHY1 mRNA expression after 72 h of siRNA-mediated IP_3_R2 silencing. (*N* = 3). **(G)** qRT-PCR analysis showing increase in OPTN mRNA expression after 72 h of siRNA-mediated IP_3_R2 silencing. (*N* = 3). **(H)** Representative images of confocal imaging in B16 cells transfected with siRNA along with mCherry-Tyrosinase-EGFP probe, scale bar, 5 µm. **(I)** Bar graph shows the quantification of the autolysosome intensity in the GFP:RFP ratio, where ‘*N*’ denotes the number of cells. **(J)** Representative images of confocal imaging in B16 cells transfected with siRNA along with mCherry-Tyrosinase-EGFP probe, scale bar, 5 µm. **(K)** Bar graph shows the quantification of the autolysosome intensity in the GFP:RFP ratio, where ‘*N*’ denotes the number of cells. **(L)** A schematic illustration demonstrating that IP_3_R2 silencing augments melanophagy, by releasing Lysosomal Ca^2+^ via TRPML1 which subsequently translocated TFEB into the nucleus. Created in BioRender. Motiani, R. (2026) https://BioRender.com/h5bus03. Data presented are mean ± SEM. For statistical analysis, one-sample *t* test was performed for panels B, D, F, G, and Tukey’s multiple comparisons test was performed for panels I and K, using GraphPad Prism software. Here, * *p* < 0.05, *** *p* < 0.001 and **** *p* < 0.0001.

Our data clearly demonstrates that downstream of IP_3_R2 silencing TRPML1 facilitates augmented lysosomal Ca^2+^ release and enhanced melanophagy. Next, we investigated the molecular mechanism through which TRPML1 drives melanophagy. Recent literature suggests that TRPML1-mediated lysosomal Ca^2+^ release activates autophagy via transcription factor EB (TFEB) nuclear translocation [66,67,73,74]. Therefore, we investigated the potential role of TFEB in enhancing melanophagy upon IP_3_R2 knockdown. Our confocal microscopy data using pEGFP-N1-TFEB shows an increase in TFEB nuclear localization in IP_3_R2-silenced condition compared to the control condition (Fig 8A and 8B). We further validated this observation by performing subcellular fractionation followed by probing nuclear-to-cytosolic ratio of TFEB via immunoblotting. This analysis revealed an increase in nuclear-to-cytoplasmic ratio of TFEB in the IP_3_R2 knockdown condition compared to control (Fig 8C and 8D). Collectively, the microscopy and biochemical findings show that there is elevated TFEB nuclear translocation upon IP_3_R2 silencing suggesting that it can be a likely mechanism underlying the enhanced melanophagy observed upon IP_3_R2 downregulation.

Previous studies have demonstrated that TFEB enhances transcription of several autophagic genes [66,75,76]. We performed qRT-PCR analysis in IP_3_R2-silenced and corresponding control cells. We observed that IP_3_R2 silencing led to an increase in the mRNA levels of several genes (Beclin1, MAP1LC3B, ATG9B and ATG5) involved in the initiation and progression of autophagy (S11A–S11D Fig). Recently, ring finger and CHY Zinc finger domain containing 1 (RCHY1, an E3-ligase) and optineurin (OPTN, a melanophagy receptor) were reported as key regulators of melanophagy [77]. We next examined if TFEB transcriptionally controls RCHY1 and OPTN to drive melanophagy. First, we performed extensive bioinformatic analysis using the Eukaryotic Promoter Database at a very stringent p-value threshold of 0.001 and found that TFEB has multiple potential binding sites on RCHY1 and OPTN promoters (Fig 8E). We corroborated this observation via qRT-PCR experiments, which revealed an increase in RCHY1 and OPTN expression upon IP_3_R2 knockdown suggesting that TFEB-mediated upregulation of these genes could be responsible for the IP_3_R2 silencing-induced melanophagy (Fig 8F and 8G). We further validated significance of RCHY1 and OPTN in IP_3_R2 knockdown-induced melanophagy. We performed RCHY1 and OPTN loss-of-function experiments. We initially conducted qRT-PCR analysis to evaluate the knockdown efficiency of RCHY1 and OPTN siRNAs. This analysis revealed that siRNAs effectively decrease RCHY1 and OPTN levels (S11E and S11F Fig). We subsequently evaluated the melanophagy using melanophagy probe, mCherry-Tyrosinase-EGFP upon co-silencing of IP_3_R2 and either OPTN or RCHY1 gene. Our analysis revealed a significant reduction in melanophagy with both RCHY1 and OPTN co-silencing compared to IP_3_R2 silencing alone (Fig 8H–8K). This indicates that downstream IP_3_R2 silencing, RCHY1 and OPTN are the critical regulators of melanophagy flux. Collectively, our data reveals that upon IP_3_R2 knockdown there is enhanced TRPML1-mediated lysosomal Ca^2+^ release, which leads to TFEB nuclear translocation and transcriptional upregulation of melanophagy regulators (RCHY1 and OPTN) to drive melanophagy (Fig 8L).

In addition to TRPMLs, other Ca^2+^ efflux channels, such as TPC2, are present on lysosomes and may contribute to the process of melanophagy following the IP_3_R2 silencing [78,79]. To evaluate this possibility, we conducted Ca^2+^ imaging using TPC2-A1-N (TPC2 activator) to assess TPC2 activity following IP_3_R2 silencing. Our findings revealed no significant changes in TPC2 activity compared to the siNT control (S11G and S11H Fig). We extended our investigation to evaluate TFEB translocation upon TPC2 activation downstream IP_3_R2 silencing. Confocal microscopy was performed to assess the nuclear/cytoplasmic ratio of TFEB. Our findings indicated no significant changes in TFEB translocation upon TPC2 activation in siIP_3_R2 condition compared to siNT control (S11I and S11J Fig). Taken together, these data sets suggest that downstream of IP_3_R2 silencing TPC2 channel does not contribute to melanophagy regulation.

In summary, our work reveals that IP_3_R2 acts as a critical driver of pigmentation by keeping a check on melanophagy. Our unbiased meta-analysis demonstrate that IP_3_R2 levels are higher in African skin samples in comparison to White skin thereby highlighting potential physiological relevance of IP_3_R2 in skin pigmentation. Indeed, our in vitro studies in multiple independent pigmentation models and in vivo experiments in zebrafish demonstrate that IP_3_R2’s ER Ca^2+^ release function is required for controlling pigmentation levels. Further, we report that IP_3_R2-mediated ER–mitochondrial and ER–lysosomal crosstalk is a crucial regulator of melanophagy. Our data show that IP_3_R2 silencing decreases mitochondrial Ca^2+^ whereas it increases lysosomal Ca^2+^ levels. This in turn induces melanophagy via AMPK-ULK and TRPML1-TFEB signaling cascades, respectively. Hence, this work uncovers that IP_3_R2-mediated Ca^2+^ signaling across organelles is a critical determinant of melanophagy and consequently skin pigmentation. Therefore, this signaling module may offer potential therapeutic options for the management of pigmentary disorders and skin malignancies. Hence, future studies focused on studying the role of IP_3_R2 and the signaling axes revealed in this work are warranted in skin pigmentary diseases and melanomas.

## Discussion

**Fig 9:**
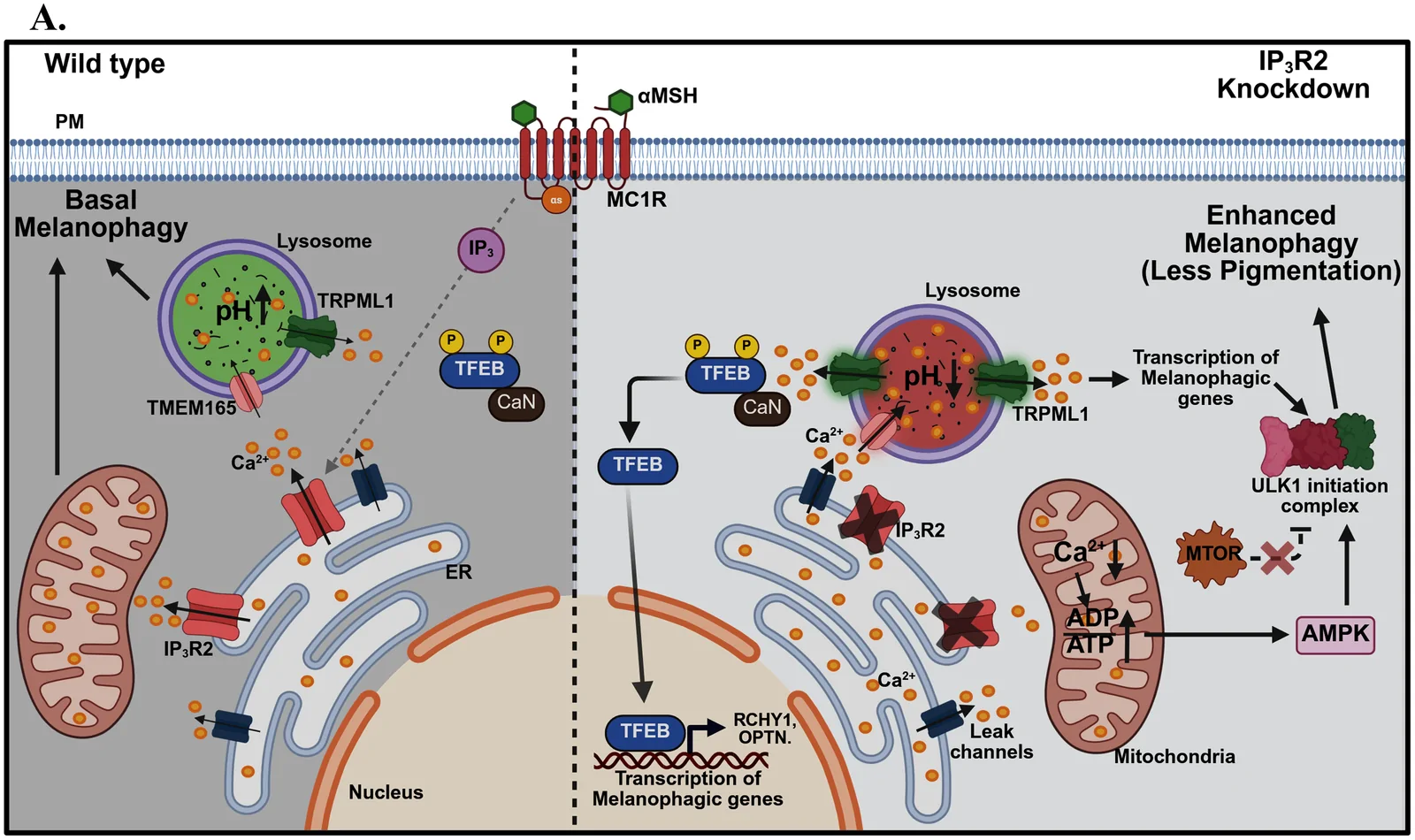
IP_3_R2 regulates melanophagy via inter-organelle Ca^2+^ dynamics. Schematic model illustrating the role of IP_3_R2-mediated Ca^2+^ signaling in regulating melanophagy. In wild-type melanocytes, αMSH–MC1R–IP_3_ signaling activates IP_3_R2, facilitating ER–mitochondrial Ca^2+^ transfer and mitochondrial ATP production. Lysosomal pH homeostasis limits excessive TRPML1-dependent Ca^2+^ release, keeping TFEB largely cytosolic, thereby leading to basal melanophagy. IP_3_R2 knockdown disrupts mitochondrial Ca^2+^ uptake, induces energy stress and activates AMPK–ULK1 signaling, which supports melanophagy. The increased proximity of the lysosome to the ER and Ca^2+^ release through ER leak channels elevates lysosomal Ca^2+^ levels. The lowered lysosomal pH facilitates TRPML1-mediated Ca^2+^ efflux, which dephosphorylates TFEB**,** promoting its nuclear translocation, transcription of melanophagy regulators (RCHY1 and OPTN), increased melanophagy, and thereby reduced pigmentation. Created in BioRender. Motiani, R. (2026) https://BioRender.com/h5bus03.

Inter-organelle communication is emerging as a key determinant of cellular physiology and a critical regulator of pathological outcomes. However, role of inter-organelle crosstalk in melanocyte function and pigmentation biology remains poorly understood. Disruptions in pigmentation pathways contribute to the development of pigmentary disorders, such as vitiligo and melasma. Further, they predispose to skin cancers, which are highly metastatic and associated with poor prognosis. Pigmentation is an outcome of balance between melanosome biogenesis and degradation. Although signaling modules that drive melanosome biogenesis are partially understood, the molecular mechanisms regulating melanophagy process remain largely unappreciated. Here, we reveal that IP_3_R2 positively regulates pigmentation by suppressing melanophagy (Fig 9). IP_3_R2-mediated ER-mitochondrial and ER-lysosomal Ca^2+^ signaling is a crucial modulator of melanosome degradation. IP_3_R2 silencing decreases mitochondrial Ca^2+^ uptake (Fig 5) while it increases lysosomal Ca^2+^ concentration (Figs 6–8). This in turn collectively enhances melanophagy and consequently decreases pigmentation.

Our data demonstrate that IP_3_R2 knockdown leads to an increase in stability of melanogenic proteins despite a concurrent decrease in pigmentation (S2 Fig). We performed live cell melanophagy assessment assays using two de novo generated ratiometric probes to show that IP_3_R2 silencing induces melanophagy (Figs 4 and S3). We further validated these results by performing biochemical assays (S4 Fig) and ultrastructural studies (S5 Fig). Mechanistically, the IP_3_R2 knockdown stimulates melanophagy via AMPK-ULK and TRPML1-TFEB signaling cascades that work downstream of ER-mitochondria and ER-lysosomal Ca^2+^ signaling, respectively. Interestingly, the role of ER Ca^2+^ transients was recently reported in stimulating autophagosome formation [80,81]. It was shown that IP_3_R2 silencing in Cos7 kidney cells increases lysosomal Ca^2+^ stores. This in turn led to enhanced lysosomal Ca^2+^ efflux that initiates formation of FIP200 puncta on ER membrane. These ER-associated FIP200 are crucial for autophagosome formation [80]. Although it was suggested that ER Ca^2+^ transients regulate lysosomal Ca^2+^ content and lysosomal Ca^2+^ efflux, the underlying molecular mechanisms responsible for increase in lysosomal Ca^2+^ stores and augmented lysosomal Ca^2+^ release remain unappreciated. Here, we show that IP_3_R2 silencing bring ER-lysosome closer and that results in higher lysosomal Ca^2+^ uptake via TMEM165 (Figs 6 and S9). Further, we demonstrate IP_3_R2 knockdown results in a decrease in lysosomal pH, which subsequently enhances the activity of the TRPML1 channel resulting in higher lysosomal Ca^2+^ release (Figs 6 and 7). Further, TRPML1-mediated lysosomal Ca^2+^ release drives melanophagy (Fig 7) by enhancing TFEB nuclear translocation and transcription of key melanophagy regulators RCHY1 and OPTN (Fig 8).

Interestingly, our quantitative TEM analysis reveals that ER-lysosome distance is significantly reduced upon IP_3_R2 knockdown, whereas the frequency of their contact events remains unchanged. This suggests that proximity and contact number represent dissociable features of organelle architecture in our system. One possible explanation of this observation could be a Ca^2+^-dependent reorganization of the ER network rather than an increase in tethering machinery. This ER reorganization can in turn enhance lysosomal proximity, without increasing the contact numbers, which supports the enhanced TMEM165-mediated lysosomal Ca^2+^ loading (Fig 6). However, further experiments would be required to test this hypothesis in future studies.

In this study, we generated and characterized two de novo ratiometric probes to measure melanophagy (Figs 4 and S3). As both the probes are single-construct ratiometric reporters with fixed fluorophore stoichiometry, cell-to-cell differences in absolute expression are intrinsically normalized within the ratio itself. Further, to confirm probe localization, we performed co-localization analysis with the melanosomal marker HMB45. This analysis showed a strong overlap for both the mCherry-Tyrosinase-eGFP probe (PCC ~0.80, OC ~0.85) and the Tyrosinase-mKeimaN1 probe (PCC ~0.80, OC ~0.80), confirming that the probes target to melanosomes (S3 Fig). Next, we used autophagy inducer and inhibitor (positive and negative controls) to demonstrate that these probes that detect changes in the melanophagy levels (S3 Fig). Importantly, we validated this with an established melanophagy inducer (RTG) to demonstrate that these probes can efficiently detect change in the melanophagy levels (S3 Fig). However, we have not yet characterized the degradation rates of these probes, which may done in future studies. These studies would help in strengthening the probes’ use for quantitative measures of melanophagy flux. Notably, our probe-based observations are independently corroborated by orthogonal biochemical assays and ultrastructural studies, which collectively strengthen the overall interpretation of melanophagy data. Therefore, we believe that these probes serve as robust and reliable tool for measuring relative melanophagy levels across different conditions.

It is important to highlight that the increased stability of melanogenic proteins (DCT, Tyrosinase, GP100) alongside enhanced melanophagy under IP_3_R2 knockdown could appear paradoxical. However, it is well established that proteasome inhibition activates autophagy as a compensatory degradation mechanism [28–32]. Indeed, our cycloheximide chase experiments show that DCT, Tyrosinase, and GP100 exhibit significantly higher stability under IP3R2 silencing (S2 Fig), consistent with impaired proteasomal degradation as reported earlier [26,27]. Further, we observed a substantial increase in the LC3II/LC3I ratio following combined IP_3_R2 silencing and MG132 treatment (Fig 3). Further, IP_3_R2 silencing alone significantly increased the LC3II/LC3I ratio in both the LD and αMSH-induced pigmentation models, indicating that autophagy induction occurs downstream of IP_3_R2 knockdown. Together, these data support a model in which, IP_3_R2 knockdown stabilizes melanogenic proteins via reduced proteasomal turnover (S2 Fig), with a downstream compensatory melanophagy engagement. However, in future, direct proteasome activity measurements and ubiquitination analyses of specific melanosomal substrates will be needed to substantiate this mechanism.

Interestingly, our data reveals that IP_3_R2 silencing in melanocytes selectively activates melanophagy while sparing mitophagy and ER-phagy. Our independent lines of evidence supports the relative selectivity of IP3R2 knockdown-induced autophagy towards melanosomes. First, our live-cell ratiometric melanophagy assays using two de novo generated probes, alongside well-established mitophagy and ER-phagy reporters, demonstrate that IP3R2 silencing preferentially induces melanophagy while mitophagy and ER-phagy are comparatively spared (S5 Fig). Second, transcriptional induction of OPTN and RCHY1, the selective melanophagy receptor and E3-ligase, respectively, provides a specific molecular mechanism driving melanosome degradation. We think that the selective melanophagy upon IP_3_R2 silencing could be a cell-type-specific phenomenon. Since melanocytes use a substantial amount of energy in melanosome biogenesis and melanogenesis (their prime physiological function), they may prefer to recycle the melanosomes via melanophagy for new melanosome biogenesis. Similar equivalents are extensively reported in context of mitophagy wherein cells specifically recycle mitochondria via mitophagy for new mitochondria biogenesis to conserve the cellular resources.

Although lysosomes have other Ca^2+^ release channels such as TPCs and P2X4 channels, they most likely do not contribute to melanophagy. Indeed, literature suggest that TPCs do not contribute to TFEB translocation and thereby they would most likely not regulate melanophagy [66]. Indeed, our data shows that downstream of IP_3_R2 silencing, TPC2 does not contribute to increase in lysosomal Ca^2+^ efflux and nuclear translocation of TFEB (S11 Fig). Likewise, P2X4 and TRPML2/3 channels get activated at high pH and are inactivated at low pH [82,83] whereas IP_3_R2 silencing decreases lysosomal pH. It is important to highlight that TRPML1 is activated at low pH [84,85] and therefore, it is stimulated downstream of IP_3_R2 knockdown.

Interestingly, an earlier study reported that TRPML3 gain-of-function mutant mice show depigmentation of fur and tail [86]. Though authors suggested loss of melanocyte function as a possible driver of depigmentation, the underlying molecular mechanism that induce depigmentation in this mice remain elusive. In future, it would be interesting to study the role of TRPML3-mediated lysosomal Ca^2+^ efflux in melanophagy to examine if it leads to depigmentation by enhancing melanophagy. Secondly, in the future studies, it would be worth to generate a TRPML1 gain-of-function mice and investigate its effect on mice pigmentation.

Intriguingly, our data demonstrates that IP_3_R2, but not IP_3_R1 and IP_3_R3, selectively regulate melanophagy. The most likely explanation for this could be the level of IP_3_R isoform expression. Indeed, Human Protein Atlas data show IP_3_R2 as the predominant IP_3_R isoform in melanocytes, with IP_3_R1 and IP_3_R3 expression at comparatively very low level. Further, IP_3_R2, but neither IP_3_R1 nor IP_3_R3, overexpression rescued the IP_3_R2 knockdown-induced decrease in ER Ca^2+^ efflux (S6 Fig) suggesting that expression levels may not be the only explanation. Another possibility could be that endogenous IP_3_R2 acts as the principal isoform populating ER subdomains apposed to mitochondria and lysosomes in melanocytes. Therefore, the direct isoform-resolved co-localization studies at these contact sites remain an important avenue for future work.

It is important to note that IP_3_R2 knockdown could in principle perturb ER Ca^2+^ handling broadly, rather than engaging a dedicated melanophagy-regulatory mechanism. However, several lines of evidence support physiological relevance. αMSH, a physiological melanogenic stimulus, triggers IP_3_R-dependent Ca^2+^ release [17,18], and αMSH-induced pigmentation is reduced upon IP_3_R2 silencing. Further, IP_3_R2 expression correlates with pigmentation levels across multiple microarray datasets (Fig 1). Importantly, IP_3_R2 expression is reduced in lighter-pigmented White skin as compared to darkly pigmented African skin (Fig 1). Moreover, rescue experiment using pore-dead IP_3_R2 mutant (IP_3_R2-M) demonstrates that IP_3_R2 promotes pigmentation through its Ca^2+^ channel activity rather than a structural role. Notably, all these findings are recapitulated in vivo in zebrafish pigmentation model. Collectively, our findings identify IP_3_R2 as a potential regulator of pigmentation. However, further validation in human pigmentary disorders and skin cancers is required before considering therapeutic applications.

Taken together, this study reveals an intricate interplay between IP_3_R2-mediated Ca^2+^ dynamics across organelles and melanophagy. Our work show that IP_3_R2 silencing reduces mitochondrial Ca^2+^ uptake and simultaneously increases lysosomal Ca^2+^ levels. This in turn enhances melanophagy via AMPK-ULK and TRPML1-TFEB signaling modules, respectively. In summary, we demonstrate that Ca^2+^ signaling across organelles is a crucial regulator of melanophagy and thereby pigmentation. Hence, this study holds the potential to unlock new avenues for treating pigmentary disorders and developing targeted strategies to manage skin malignancies.

## Materials and methods

### Ethics statement

Zebrafish used in this study were housed in the Laboratory of Calciomics and Systemic Pathophysiology at the Regional Centre for Biotechnology, India, with proper standard ethical protocols approved by the Institutional Animal Ethics Committee of Regional Centre for Biotechnology, India, (Protocol no. RCB/IAEC/2021/086) with care to minimize animal suffering. The animal care protocols adhered to the guidelines of the Committee for Control and Supervision of Experiments on Animals (CCSEA), India.

### Cell culture

B16-F10 murine melanoma cells were procured from the American Type Culture Collection and cultured in Dulbecco’s Modified Eagle’s Medium-high glucose (DMEM-HG; Sigma-Aldrich, D5648) supplemented with 10% fetal bovine serum (FBS; Gibco, 10270106), 1× Antibiotic Antimycotic (Thermo Fisher Scientific, 15240062). The B16-F10 cells were maintained at 60%–80% confluency in a humidified incubator with 5% CO_2_ levels. Additionally, Human Epidermal Melanocytes, neonatal, lightly pigmented donor (HEMn-LP; Gibco, C0025C) were cultured in Medium 254 (M254; Gibco, M254CF) supplemented with human melanocyte growth supplement-2 (HMGS-2; Gibco, S0165) 1× Antibiotic Antimycotic (Thermo Fisher Scientific, 15240062) under the same environmental conditions. Experiments were conducted using cells between passages 3 and 6. Essential cell culture reagents used during experiments were phosphate-buffered saline, pH 7.2 (PBS; HIMEDIA, M1452), Trypsin (2.5%), no phenol red (Gibco, 15090046).

### Microarray analysis

The publicly accessible microarray dataset (accession ID: GSE54638) was analysed using the GEO2R tool available at the microarray data repository Gene Expression Omnibus (https://www.ncbi.nlm.nih.gov/geo/geo2r) of NCBI. Whole-skin gene expression levels of the IP_3_R2 gene were compared between two ethnic populations, African and White. Normalized expression values after log transformation are visualized using the SRplot heatmap tool.

### Low-density (LD) pigmentation model system

B16 cells were seeded at an initial density of 100 cells/cm^2^ in Dulbecco’s Modified Eagle’s Medium supplemented with 10% fetal bovine serum, as described previously [17,22]. The cells were then permitted to develop pigmentation gradually, and the experiments were terminated on Day 6 or Day 7.

### siRNA transfection

Murine B16 melanoma cells were seeded at a density of 60,000 cells/well in a 6-well culture plate. The next day, transfection was performed using 50 nM of siRNA (see Table 1) incubated with TurboFect Transfection Reagent (Thermo Fisher Scientific, R0531) for 30 min in 1:3 ratio (v/v) in Opti-MEM Reduced Serum Medium (Gibco,31985070). The transfection mixture was then added to the wells of 6-well plates. After 24 h, melanocyte-stimulating hormone (αMSH; Sigma-Aldrich, M4135) was added for 48 h, followed by cell harvesting. If not otherwise stated.

**Table 1:** List of siRNA.

siRNA (common name)	Species	Manufacturer	Catalog no.	Target sequence
siITPR1 (siIP_3_R1)	Mouse	Dharmacon	L-040933-00-0005	GGUCAUCGAUCUCAUAAUG, AAAAUAAGCUCACGUUUGA, GACCGAGAGUAUUCCAUGAAA, CAACUAAGGUCCAUUGUGG
siITPR2 (siIP_3_R2)	Mouse	Dharmacon	L-041018-00-0005	CCACAAAGAUCACGAUUCA, CGGAAGAUCUCUACAACGA, GGAAGAGGGUAGCACAUUA, GAGAGGAGGUUUGUGACUA
siITPR3 (IP_3_R3)	Mouse	Dharmacon	J-065715-07-0005	GAAGAUUGCUGAUGUGGUA
siMCOLN1 (siTRPML1)	Mouse	Dharmacon	L-044469-00-0005	UGAGAUCCCUGAUUGUUAC, UCAUCUACCUGGGCUAUUG, AGAUUGGCAUUGAGGCAAA, GCAGUAUUCUCUUGGGUAC
siITPR2 (siIP_3_R2)	Human	Dharmacon	L-006208-02-0005	CGAAAUGGCCGCUCUAUUA, GCAAUGGGUUGGAGACUAU, GGAAUGAAAGGGCAAUUAA, GAGCAAUAACUACCGGAUU
siNT	**–**	Dharmacon	D-001810-10-20	UGGUUUACAUGUCGACUAA, UGGUUUACAUGUUGUGUGA, UGGUUUACAUGUUUUCUGA, UGGUUUACAUGUUUUCCUA
siTMEM165	Mouse	Eurogentec	–	GCCCCAGTTCATACCAACAAA
siOPTN	Mouse	Dharmacon	J-041705-05-0002	GCUAUGAAAGGGCGAUUUG
siRCHY1	Mouse	Dharmacon	J-065323-09-0002	AGUCACAUUCAUCGAAAUA

On day 3 of the low-density pigmentation model, 100 nM of siRNA (see Table 1) was incubated with DharmaFECT 2 Transfection Reagent (Dharmacon, T-2002-03) at a 1:3 ratio (v/v) in Opti-MEM Reduced Serum Medium for 45 min. The original LD day 3 media was saved, and the transfection mixture was added to the cells for 6 h. Subsequently, the saved day 3 media was replaced, with the addition of 1 μM αMSH. The cells were harvested on Day 6.

For HEMn-LP, the cells were first trypsinized, and a cell pellet containing 7–10 × 10^5^ cells was obtained. The cells were then resuspended in 100 µL of the Nucleofector Solution for Human Melanocytes—Neonatal, provided in the Human Melanocyte-Neonatal Nucleofector Kit. (Lonza, VPD1003). Subsequently, 5 µM siRNA (see Table 1) was added to this cell suspension. The cell-siRNA mixture was transferred to an Aluminum Cuvette (included in the kit) and nucleofection was performed using the U-024 program of the Nucleofector 2b device. After the electroporation, the cells were resuspended in Medium 254 supplemented with Human Melanocyte Growth Supplement-2 (HMGS-2) and then seeded in a culture flask for a 72 h incubation period.

### Lentiviral stable cell line generation

To achieve stable knockdown, mouse-specific short hairpin RNAs (shRNAs) targeting the non-targeting control (shNT) and IP_3_R2 (shITPR2) were obtained from Transomics Technologies and cloned into the lentiviral vector pGIPZ-mCMV-mCherry-Puromycin. As previously reported, the lentiviral constructs pCMV-VSVG (Addgene, 8454), pCMV-dR8.2 (Addgene, 8454), and pGIPZ-shNT/shITPR2 (Transomics Technologies, TLMSU1452) were mixed in a 1:3:2 ratio and combined with Lipofectamine 2000 (Thermo Fisher Scientific, 11668019) at a 1:2 ratio (w/v) in Opti-MEM medium [21,87]. The mixture was incubated for 45 min and then added to a flask containing 90% confluent HEK293T cells for 6 h. The transfection mixture was then removed, and DMEM-HG supplemented with 2% Tet-negative fetal bovine serum was added. Viral particles were collected 48 h post-transfection and concentrated using Amicon filters through centrifugation at 4,000 rpm for 30 min in a swinging bucket. The concentrated viral particles, along with 10 μg/mL polybrene, were then added to B16 cells seeded at 50% confluency in a T25 flask for a 48 h transduction period. Subsequently, 5 μg/mL of puromycin was applied to select the transduced cells. The knockdown was validated through western blot analysis.

### RNA extraction and real-time PCR

Total RNA was isolated from the cell samples using the RNeasy Mini Kit (Qiagen, 74104) following the manufacturer’s protocol. Complementary DNA was then synthesized from the extracted RNA through reverse transcription using the High-Capacity cDNA Reverse Transcription Kit (Thermo Fisher Scientific, 4368814)) following the manufacturer’s protocol. Subsequently, quantitative real-time PCR with complimentary DNA (cDNA) was conducted using SYBR green (Takara, RR420A), following the manufacturer’s protocol utilizing the Quant Studio 6 Flex from Applied Biosystems or CFX96 Touch Real-Time PCR Detection System from Bio-Rad. The expression analysis was normalized against the GAPDH housekeeping gene unless otherwise stated. Primers used in this study are listed in Table 2.

**Table 2:** List of qRT-PCR primers (FP stands for forward and RP for reverse primer).

Gene name	Sequence	Species
ITPR1 (FP)	ACATCCTCGCTCACCAGTTG	Mouse
ITPR1 (RP)	TGTTCCATTGTCCGGTCCAG	Mouse
ITPR2 (FP)	GTGGATATGGCGAGGGTCTG	Mouse
ITPR2 (RP)	GGCTGGTACTGTTGTCGGAA	Mouse
ITPR3 (FP)	AGGCTCCATTCCGTGACAAG	Mouse
ITPR3 (RP)	TGCGGTAGTCCTCCTGAGAA	Mouse
ITPR2 (FP)	GATGAGAGTGGCCCCTTAGC	Human
ITPR2 (RP)	CCCTCACTATGTCGTCCAGC	Human
DCT (FP)	TAATTGTGGAGGCTGCAAGTTC	Mouse
DCT (RP)	AGGATGGCCGGCTTCTTC	Mouse
TYR (FP)	CGTAATCCTGGAAACCATGACA	Mouse
TYR (RP)	GTCAAACTCAGACAAAATTCCACATC	Mouse
GP100 (FP)	AAGTGCTGAAAGGTGGCTCA	Mouse
GP100 (RP)	CCACCGTCTTGACCAGGAAC	Mouse
TMEM165 (FP)	TCATAGTGTCCGAACTCGGCGA	Mouse
TMEM165 (RP)	GGATGACTGTTGTGGCATAGCC	Mouse
BECN1 (FP)	CAGCCTCTGAAACTGGACACGA	Mouse
BECN1 (RP)	CTCTCCTGAGTTAGCCTCTTCC	Mouse
ATG9B (FP)	GAGCAGGACTATGAACGGCTAG	Mouse
ATG9B (RP)	GTCCAGGTTCTGGATGTGATGC	Mouse
MAP1LC3B (FP)	GCTTGCAGCTCAATGCTAAC	Mouse
MAP1LC3B (RP)	CCTGCGAGGCATAAACCATGTA	Mouse
ATG5 (FP)	CTTGCATCAAGTTCAGCTCTTCC	Mouse
ATG5 (RP)	AAGTGAGCCTCAACCGCATCCT	Mouse
ATP6VOD1 (FP)	GCATCTCAGAGCAGGACCTTGA	Mouse
ATP6VOD1 (RP)	GGATAGGACACATGGCATCAGC	Mouse
ATP6V1H (FP)	GTTGCTGCTCACGATGTTGGAG	Mouse
ATP6V1H (RP)	TGTAGCGAACCTGCTGGTCTTC	Mouse
MCOLN1 (FP)	GCGCCTATGACACCATCAA	Mouse
MCOLN1 (RP)	TATCCTGGCACTGCTCGAT	Mouse
MCOLN2 (FP)	TACGTCCTGGTCACTATCAGCG	Mouse
MCOLN2 (RP)	GAGCAAGATGCTGCACACGTCA	Mouse
MCOLN3 (FP)	CTACAGGCAAGGAACCATCTGC	Mouse
MCOLN3 (RP)	TGTGGAAGTCCAGGCTCAGGTT	Mouse
RCHY1 (FP)	GCCTAACCACGAATCTTCGAGG	Mouse
RCHY1 (RP)	ACACGGCAAGACGTGAGCAACA	Mouse
OPTN (FP)	AGGTGGAGAGACTTGAAGTCGC	Mouse
OPTN (RP)	TCCTCGCTGTCTGCTTCTCAGT	Mouse
ITPR2 (FP)	CTGCATTGAAACTCACGGCC	Zebrafish
ITPR2 (RP)	CAGCACGTCTTCTCCACCAT	Zebrafish
Actb1 (FP)	GGCAATGAGCGTTTCCGTTG	Zebrafish
Actb1 (RP)	TACCGCAAGATTCCATACCCAG	Zebrafish

### Antibodies and western blot

Total cellular protein extracts were prepared by solubilizing the samples in a buffer containing Membrane-bound Protein Extraction Buffer (10 mM Tris-HCl, 100 mM NaCl, 1 mM EDTA, 1 mM EGTA, 1 mM NaF, 20 mM Na4P2O7, 2 mM Na3VO4, 1% Triton X-100 (v/v), 10% Glycerol, 0.5% sodium deoxycholate, 0.01% SDS (w/v), at pH 7.4) and cOmplete Mini, EDTA-free Protease Inhibitor Cocktail (Roche, 4693159001) with incubation for 30 min at 4 °C. The soluble fraction was then recovered in the supernatant after spinning at 15,000 rpm for 15 min at 4 °C, and the total protein concentration was quantified using the Pierce BCA Protein Assay Kits (Thermo Fisher Scientific, 23227).

Proteins were resolved using 8%–14% gel using the SDS-PAGE method, then transferred to a 0.45 μ Polyvinylidene fluoride (PVDF) membrane using the Mini-PROTEAN Tetra electrophoresis system (BioRad). The PVDF membrane was blocked in Tris-buffered saline containing 0.1% Tween-20 and 5% BSA or non-fat dry milk for (2 h, 20 °C), washed in the same medium and incubated with the primary antibody (16 h, 4 °C) in the blocking buffer. After three washes, the membrane was incubated with the secondary antibody (2 h, 20 °C) and then washed. Protein bands were visualized using the Immobilon Forte Western HRP substrate (Millipore, WBLUF0500) in the Image Quant (LAS4000) imaging system. Densitometric quantification was performed on unsaturated images using ImageJ software. Antibodies used in this study are listed in Table 3

**Table 3:** Details of antibodies.

Antibody	Dilution	Catalog no.	Company
Beta-Tubulin	1:5,000	Ab21058	Abcam
Beclin1 (D40C5)	1:1,000	3495	Cell Signalling Technology
LC3A/B (D3U4C)	1:1,000	12741	Cell Signalling Technology
IP_3_R-II (A-5)	1:1,000	sc-398434	Santa Cruz Biotechnology
Anti-TRP2/DCT	1:2,000	Ab74073	Abcam
Anti-Melanoma GP100	1:2,000	Ab137078	Abcam
Tyrosinase	1:2,000	35-6000	Invitrogen
ULK1 (D8H5)	1:1,000	8054	Cell Signalling Technology
Phospho-ULK1 (Ser757)	1:1,000	6888	Cell Signalling Technology
AMPKα (D5A2)	1:1,000	5831	Cell Signalling Technology
Phospho-AMPKα (Thr172)	1:1,000	2535	Cell Signalling Technology
p70 S6 Kinase (49D7)	1:1,000	9202	Cell Signalling Technology
Phospho-p70 S6 Kinase (Thr389)	1:1,000	97596	Cell Signalling Technology
4E-BP1 (53H11)	1:1,000	9644	Cell Signalling Technology
Phospho-4E-BP1 (Thr37/46) (236B4)	1:1,000	2855	Cell Signalling Technology
mTOR (7C10)	1:1,000	2983	Cell Signalling Technology
Phospho-mTOR (Ser2448) (D9C2)	1:1,000	5536	Cell Signalling Technology
TFEB	1:1,000	4240	Cell Signalling Technology
Lamin	1:1,000	MA5–35284	Invitrogen
Beta-Actin	1:10,000	A3854	Sigma
Calnexin	1:2,000	Ab22595	Abcam

### Colocalization immunofluorescence assay

B16 cells cultured on glass slides were transfected with either 1 µg mCherry-Tyrosinase-EGFP or Tyrosinase-mKeimaN1 using TurboFect Transfection Reagent as per the manufacturer’s protocol. After 48 h, the cells were washed with PBS and fixed with 4% paraformaldehyde in PBS for 15 min. After three washes with PBS, the cells were permeabilized with PBS containing 0.3% Triton-X100 for 15 min and blocked with PBS containing 2% bovine serum albumin and 0.1% Triton-X100 for 1 h. The cells were then incubated with anti-HMB45 antibody (Thermo Fisher Scientific, MA1-34759) at 1:40 ratio for overnight at 4℃. After incubation, the cells were washed thrice with PBS containing 0.1% Tween20 and incubated with Alexa Fluor 647 chicken anti-mouse cross-adsorbed secondary antibody (1:500) (Thermo Fisher Scientific, A21463) at 25 °C for 2 h. Finally, the cells were washed three times with PBS containing 0.1% Tween20 mounted using SlowFade Gold antifade moutant with 4′,6-diamidono-2-phenylindole (DAPI) (Thermo Fisher Scientific, S36938). Images were acquired using confocal microscopy equipped with a 63X/1.40 oil immersion objective (Laser Scanning Confocal Microscope: LSM 880, Carl Zeiss). Cells were excited with 633 nm, 543 nm, 488 nm and 405 nm lasers in an alternating manner, and the resulting fluorescence emissions were captured at 650–707 nm, 573–625 nm, 508–548 nm and 420–487 nm ranges, respectively. The confocal images were then deconvoluted using ZEN 2.3 SP1 FP1 (black) software (version 14.0) and analyzed using Fiji (Image J) software, with background signals subtracted from each acquired images during quantitative analysis.

Similarly, to check colocalization between LC3(A/B) and HMB45 upon siRNA silencing, B16 cells cultured on glass slides were transfected with either siNT or siIP_3_R2 using TurboFect Transfection Reagent as per the manufacturer’s protocol. After 48 h, cells were fixed, permeabilized and blocked following the above protocol. The cells were then incubated with anti-HMB45 antibody (Thermo Fisher Scientific, MA1-34759) at 1:40 ratio along with anti- LC3A/B (Cell signalling Technologies, 12741) at 1:200 ratio for overnight at 4 ℃. After incubation, the cells were washed thrice with PBS containing 0.1% Tween20 and incubated with Alexa Fluor 488 goat anti-rabbit-conjugated secondary antibody (1:500) (Thermo Fisher Scientific, A11070) along with Alexa Fluor 568 goat anti-mouse-conjugated secondary antibody (1:500) (Thermo Fisher Scientific, A11019) at 25 °C for 2 h. Finally, the cells were washed three times with PBS containing 0.1% Tween20 mounted using SlowFade Gold antifade mountant with 4′,6-diamidono-2-phenylindole (DAPI) (Thermo Fisher Scientific, S36938). Images were acquired using confocal microscopy equipped with a 63×/1.40 oil immersion objective (Laser Scanning Confocal Microscope: LSM 880, Carl Zeiss). Cells were excited with 543 nm, 488 nm and 405 nm lasers in an alternating manner, and the resulting fluorescence emissions were captured at 573–625 nm, 508–548 nm and 420–487 nm ranges, respectively. The confocal images were then deconvoluted using ZEN 2.3 SP1 FP1 (black) software (version 14.0) and analyzed using Fiji (Image J) software, with background signals subtracted from each acquired images during quantitative analysis.

### Melanin content assay

The melanin content of the cellular samples was quantified using a previously described experimental protocol [17,21]. Equal numbers of cells from each condition were lysed in 1N Sodium hydroxide solution (NaOH) through heating at 80 °C for 3–4 h. The samples were then centrifuged to remove any cellular debris. The absorbance of the resulting supernatants was measured at 405 nm using a Fluorescence Spectrophotometer. The melanin content of the samples was estimated by comparing their absorbance values to a standard curve (µg/ml) generated using synthetic melanin. In some samples, mean pixel intensity of cell pellet was calculated using image J (NIH).

Melanin content of the zebrafish embryos: Equal number of Zebrafish embryos (~20 embryos) were lysed in 10N NaOH through heating at 80 °C for 3–4 h. The samples were then centrifuged to remove any cellular debris. The absorbance of the resulting supernatants was measured at 405 nm using a Fluorescence Spectrophotometer. The melanin content of the samples was estimated by comparing their absorbance values to a standard curve (µg/ml) generated using synthetic melanin.

### Cytosolic Ca^2+^ imaging

Intracellular Ca^2+^ levels were assessed through fluorescence imaging, as described in prior reports [16,88]. Cells were cultured on confocal dishes (SPL Life Sciences, 200350) and incubated with the ratiometric Ca^2+^ indicator fura-2AM (Thermo Fisher Scientific, F1221) for 30 min at 37 °C. After washing the cells in Hepes-buffered saline solution and Ca^2+^-free HBSS buffer, specific cells were selected at 20× dry objective and imaged using a digital fluorescence microscopy system (Nikon Eclipse Ti2 microscope equipped with a CoolLED pE-340 Fura light source and a high-speed PCO camera). Fura-2AM was alternately excited at 340 and 380 nm, and the corresponding emission signals were captured at 510 nm. The resulting Ca^2+^ traces represent the average response from multiple cells within a single imaging dish, with the number of cells (N) indicated for each trace.

### Mitochondrial Ca^2+^ imaging

Intracellular mitochondrial Ca^2+^ levels were measured using fluorescence-based Ca^2+^ imaging as described previously [18]. B16 cells were seeded on confocal dishes. After the required treatment and transfections, 1.5 μg of pCMV CEPIA2mt plasmid (a gift from Masamitsu lino, Addgene, 58218) per dish was transfected using TurboFect at a 1:2 ratio (w/v). The following day, the cells were washed three times with Ca^2+^-free HEPES-buffered saline solution and incubated in HEPES-buffered saline solution. Several cells were selected using a 60× oil objective, and imaging was performed using a digital fluorescence microscopy system (Nikon Eclipse Ti2 microscope equipped with a CoolLED pE-340 Fura light source and a high-speed PCO camera). The CEPIA2mt-transfected cells were excited at 488 nm, and the emission signal was captured at 500–550 nm. Fifty μM histamine was used as a stimulus to release Ca^2+^ from the IP_3_ receptor channels, and the resulting changes in mitochondrial Ca^2+^ were measured using the CEPIA2mt probe.

B16-F10 cells were cultured in cover-glass bottom dishes to study the functional role of IP_3_R2. Rescue experiments were conducted by overexpressing 1.5 μg of plasmid encoding either wild-type IP_3_R2, mutant variant IP_3_R2 M, IP_3_R1 or IP_3_R3 (all gift from Dr. David Yule) in cells with stable IP_3_R2 knockdown. Cultured B16 cells (IP_3_R2^+/+^, IP_3_R2^−/−^) were seeded in cover-glass bottom dishes and transfected with 1.5 μg of the CEPIA2mt Ca^2+^ indicator construct, along with 1 μg of either the mouse IP_3_R2 or the mouse IP_3_R2 mutant plasmid, using a 1:2 ratio (w/v) of TurboFect transfection reagent at 50%–60% confluency. After 48 h, several transfected cells were selected, and intracellular Ca^2+^ dynamics were examined using fluorescence-based Ca^2+^ imaging as described previously.

### TRPML1-G-GECO1.2-ERES Ca^2+^ imaging

Lysosomal Ca^2+^ measurements were conducted as previously described [71]. Following the necessary transfections, B16 cells were transfected with 1.5 μg of the TRPML1-G-GECO1.2-ERES (Addgene, 207144) construct at 50%–60% confluency in cover-glass bottom dishes. The cells were washed three times with Ca^2+^-free HBSS buffer the next day. Specific cells were selected, and imaging was performed using a digital fluorescence microscopy system with a 60X oil objective (Nikon Eclipse Ti2 microscope equipped with a CoolLED pE-340 Fura light source and a high-speed PCO camera). The TRPML1-G-GECO1.2-ERES-transfected cells were excited at 488 nm, and the corresponding emission signals were captured at 500–550 nm. Twenty μM MLSA1(Sigma-Aldrich, SML0627) or 300 μM GPN (Gly-Phe β-naphthylamide) (Santa Cruz Biotechnology, sc-252858) was utilized as a stimulus to induce Ca^2+^ release from the TRPMLs channels. The resulting Ca^2+^ traces represent the average response from multiple cells within a single imaging dish, with the number of cells (n) indicated for each trace.

B16-F10 cells were cultured in cover-glass bottom dishes to study the functional role of Mucolipin1 or TRPML1. The cells were co-transfected with 1.5 μg of TRPML1-G-GECO1.2-ERES and 1 μg of Mucolipin1-pHcRed C1 (Addgene, 62959) or the TRPML1 pore-dead mutant, Mucolipin1 D471-472K-pHcRed C1 (Addgene, 62961), at 50%–60% confluency using TurboFECT transfection reagent at a 1:2 ratio (w/v). Forty-eight h post-transfection, the cells were washed three times with Ca^2+^-free 1× HBSS buffer. Green fluorescent cells were selected, and Ca^2+^ imaging was performed using a digital fluorescence microscopy system with a 60X oil objective. Twenty μM MLSA1 as the TRPML channel agonist was used as a stimulus to induce Ca^2+^ release.

### Oregon green 488 BAPTA-1 dextran (OBDx)

Lysosomes of the cells were identified through the endocytosis of a Ca^2+^-sensitive probe, as previously described [64]. Specifically, the cells were incubated with 100 μg/mL of Oregon-BAPTA-dextran (Thermo Fisher Scientific, O6798) in the culture medium for 12 h, followed by an additional 12-hour pulse-chase period with phenol red-free DMEM to allow for lysosomal staining. The cells were then washed thrice with HBSS buffer. The intensity of the Ca^2+^-dependent fluorescence was measured using Confocal microscopy equipped with a 63×/1.40 oil immersion objective (Laser Scanning Confocal Microscope: LSM 880, Carl Zeiss). Cells were excited with a 488 nm laser, and the resulting fluorescence emission was captured within the 497–572 nm range. The Ca^2+^ fluorescence intensity was quantified using Fiji (Image J) software, with background signals subtracted from each acquired images during quantitative analysis.

### pMRX-IP-GFP-LC3-RFP plasmid transfection

Autophagy flux was analyzed using fluorescence microscopy by monitoring the degradation of the GFP-LC3 reporter, with RFP serving as an internal control, as previously described [33]. Cultured B16 cells seeded in cover-glass bottom dishes were transfected with the pMRX-IP-GFP-LC3-RFP plasmid (Addgene, 84573) using Turbofect Transfection reagent at a 1:2 ratio (w/v) for 48 h. Live cell images were acquired using confocal microscopy equipped with a 63×/1.40 oil immersion objective (Laser Scanning Confocal Microscope: LSM 880, Carl Zeiss). Cells were excited with 543 nm and 488 nm lasers in an alternating manner, and the resulting fluorescence emissions were captured at 551–632 nm and 497–572 nm ranges, respectively. The confocal images were then deconvoluted using ZEN 2.3 SP1 FP1 (black) software (version 14.0) and analyzed using Imaris or Fiji (Image J) software, with background signals subtracted from each acquired images during quantitative analysis.

### MCU Overexpression

The mitochondrial Ca^2+^ uniporter (MCU) serves as the principal channel complex facilitating the uptake of Ca^2+^ into the mitochondrial matrix [18]. B16 cells transfected with 1.5 µg of pDEST40-MCU-V5-HIS (Addgene, 31731) using the TurboFECT transfection reagent for a duration of 72 h to conduct confocal and Ca^2+^ imaging.

### TMEM165 overexpression

TMEM165, designated as a Ca^2+^/H⁺ exchanger, imports Ca^2+^ pH dependently into lysosomes [65]. B16 cells were transfected with 1 µg of pDONR221-TMEM165 (Addgene, 132272) using the TurboFECT transfection reagent for a duration of 48 h to conduct confocal and Ca^2+^ imaging.

### Cloning and transfection of mCherry-Tyrosinase-EGFP plasmid

The mCherry-Tyrosinase-EGFP melanophagy construct consists of the Tyrosinase sequence targeting specifically to Melanosome. To clone mCherry, a red fluorescent protein to pEGFP-TYR (Addgene, 32781), mCherry was amplified from vector mCherry N1(Clontech, 632523) having NHE1 and XHO1 sites present at 633–1,386 bp. Amplified Vector mcherry N1 cloned upstream of Tyrosinase-EGFP of NHE1-XHO1 site at 591–613 bp of pEGFP-TYR plasmid construct to generate mCherry-Tyrosinase-EGFP**.**

Melanophagy flux was analyzed by examining the differential stability of the green and red fluorescent proteins within the acidic lysosomal environment. B16 cells were transfected with the cloned mCherry-Tyrosinase-EGFP plasmid construct at 50%–60% confluency using a TurboFect transfection reagent at a 1:2 ratio (w/v). Twenty-four h post-plasmid transfection, live-cell images were acquired using confocal microscopy equipped with a 63×/1.40 oil immersion objective (Laser Scanning Confocal Microscope: LSM 880, Carl Zeiss). Cells were alternatingly excited with 543 and 488 nm lasers, and the resulting fluorescence emissions were captured at 551–632 nm and 497–572 nm ranges, respectively. The confocal images were then deconvoluted using ZEN 2.3 SP1 FP1 (black) software (version 14.0) and analyzed using Imaris or Fiji (Image J) software, with background signals subtracted from each acquired images during quantitative analysis.

### Cloning and transfection of Tyrosinase-mKeimaN1 plasmid

Keima is a fluorescent protein derived from coral that exhibits pH-dependent emission spectra, emitting distinct colors in acidic and neutral environments. The cumulative fluorescence signal can be used to quantify autophagy at a single time point. The Tyrosinase-mKeimaN1 melanophagy construct consists of the Tyrosinase sequence specifically targeting Melanosomes. To generate this construct, the Tyrosinase sequence was amplified from the pEGFP-TYR plasmid (Addgene, 32781), which harbors ECOR1 and KPN1 restriction sites at 629–2,231 bp. The amplified Tyrosinase sequence was then cloned upstream of the mKeima-Red-N1 vector (Addgene,54597), between the ECOR1 and KPN1 sites at 665–685 bp, to create the Tyrosinase-mKeima melanophagy reporter construct.

B16 cells were cultured and transfected with 2.5 μg of the Tyrosinase-mKeima N1 construct using TurboFECT transfection reagent at a 1:2 ratio (w/v) when the cells were 50%–60% confluent. After 48 h of plasmid transfection, live-cell imaging was performed using a confocal microscope equipped with a 63×/1.40 oil immersion objective (Laser Scanning Confocal Microscope: LSM 880, Carl Zeiss). Cells were excited with 543 and 488 nm lasers in an alternating manner, and the resulting fluorescence emissions were captured in the 556–678 nm and 493–620 nm ranges, respectively. The confocal images were then deconvoluted using ZEN 2.3 SP1 FP1(black) software (version 14.0) and analyzed using Imaris or Fiji (Image J) software, with background signals subtracted from each acquired images during quantitative analysis.

### Measurement of organelle macroautophagy

Keima, a bimodal excitation fluorescent protein was used to monitor selective organelle macroautophagy. Mitochondrial degradation (mitophagy) was measured using mKeima-Red-Mito-7 (Addgene, 56018) and Endoplasmic Reticulum degradation (ER-phagy) was measured using GST-Keima-cb5 (Addgene, 137755) fluorescent probe.

B16 cells were cultured and transfected with 1 μg of the plasmid construct using TurboFECT transfection reagent at a 1:2 ratio (w/v). After 24 h of plasmid transfection, live-cell imaging was performed using a confocal microscope equipped with a 63×/1.40 oil immersion objective (Laser Scanning Confocal Microscope: LSM 880, Carl Zeiss). Cells were excited with 543 nm and 488 nm lasers in an alternating manner, and the resulting fluorescence emissions were captured in the 556–678 nm and 493–620 nm ranges, respectively. The confocal images were then deconvoluted using ZEN 2.3 SP1 FP1(black) software (version 14.0) and analyzed using Imaris or Fiji (Image J) software, with background signals subtracted from each acquired images during quantitative analysis.

### Measurement of distance between ER and mitochondria

A split-GFP-based contact site sensor (SPLICS) has been developed to quantify the contact sites between the endoplasmic reticulum and mitochondria across a range of distances. B16 cells were transfected with 1 µg of the SPLICS Mt-ER Short P2A plasmid (Addgene, 164108) using the TurboFect transfection reagent at a 1:2 ratio (w/v). Live-cell imaging was performed 24 h after the plasmid transfection, utilizing a confocal microscope equipped with a 63×/1.40 oil immersion objective (Laser Scanning Confocal Microscope: LSM 880, Carl Zeiss). The cells were excited with 488 nm lasers, and the resulting fluorescence emissions were captured within the 497–572 nm range. The confocal images were then deconvoluted using ZEN 2.3 SP1 FP1 (black) software (version 14.0) and analyzed using Fiji (Image J) software, with background signals subtracted from each acquired images during quantitative analysis.

### Cycloheximide chase assay

B16 cells at 50%–60% confluency were transfected with siRNA using TurboFect transfection reagent at 1:3 ratio. 1 μM Melanocyte Stimulating Hormone was added 24 h post-transfection. Seventy-two h after transfection, the cells were incubated with 20 μg/ml cycloheximide (Abcam, 120093) for 0, 4, and 8 h. Subsequently, the cells were harvested, lysed, and prepared for western blotting analysis.

### Measurement of lysosomal pH

To assess changes in lysosomal pH, B16 cells grown on cover-glass bottom dishes were stained with the Lysosensor Yellow-Blue DND-160 dye at a 1:500 ratio in growth medium for 5 min in a humidified CO_2_ incubator at 37 °C. The cells were then washed thrice with 1× HBSS buffer, and live-cell imaging was performed using a confocal microscope equipped with a 63×/1.40 oil immersion objective (Laser Scanning Confocal Microscope: LSM 880, Carl Zeiss). The Lysosensor Yellow-Blue imaging was conducted by exciting the samples at 405 nm and capturing the emission signals in the 490–556 nm (yellow in more acidic organelles) and 417–481 nm (blue in less acidic organelles) ranges [89]. The confocal images were deconvoluted using ZEN 2.3 SP1 FP1 (black) software (version 14.0) and analyzed using Imaris or Fiji (Image J) software, with background signals subtracted from each acquired images during quantitative analysis.

pHluorin2 is an advanced, ratiometric, pH-sensitive green fluorescent protein. This GFP variant exhibits a dual-modal excitation spectrum, whereby acidification leads to a decrease in 405 nm excitation coupled with a corresponding increase in 488 nm excitation. Cells cultured on cover-glass bottom dishes were transfected with 0.5 μg of LAMP1-RpHLuorin2 plasmid (Addgene, 171720) using Turbofect Transfection reagent at a 1:2 ratio (w/v). After 24 h post-transfection, live-cell imaging was performed using a confocal microscope equipped with a 63×/1.40 oil immersion objective (Laser Scanning Confocal Microscope: LSM 880, Carl Zeiss). The cells were alternately excited with 405 nm and 488 nm lasers, and the resulting fluorescence emissions were captured within the 490–560 nm range. The confocal images were then deconvoluted using ZEN 2.3 SP1 FP1 (black) software (version 14.0) and analyzed using Imaris or Fiji (Image J) software, with background signals subtracted from each acquired images during quantitative analysis.

### TFEB nuclear translocation

B16 melanoma cells cultured in cover-glass bottom dishes at 50%–60% confluency were transfected with small interfering RNA (siRNA) using the TurboFect transfection reagent at a 1:3 ratio. After 24 h, 1 μM Melanocyte Stimulating Hormone was added to the cells. Forty-eight h post-siRNA transfection, the B16 cells were transfected with 1 μg of the pEGFP-N1-TFEB plasmid (Addgene, 38119) construct using the TurboFECT reagent at a 1:2 ratio (w/v). Before live-cell confocal microscopy, the cells were stimulated with 20 μM MLSA1 or 10 μM TPC2-A1-N for 1 hour. The confocal imaging was performed using a confocal microscope equipped with 63X/1.40 oil immersion objective (Laser Scanning Confocal Microscope: LSM 880, Carl Zeiss). The cells were incubated with Hoechst, a nuclear stain, at a 1:2000 dilution for 5 min to label the nuclei. The cells were then excited with 405 nm and 488 nm lasers alternatingly, and the resulting fluorescence emissions were captured within the 420–486 nm and 510–587 nm wavelength ranges, respectively. The acquired confocal images were then deconvoluted using the ZEN 2.3 SP1 FP1 (black) software (version 14.0) and analyzed using the Fiji (Image J) software, with background signals subtracted from each acquired images during quantitative analysis.

### Subcellular fractionation

The cells were collected and resuspended in a cytosolic buffer (1× PBS, 0.1% NP-40, 1 × Protease Inhibitor Cocktail). This mixture was incubated for 10 min at room temperature, representing the whole-cell lysate. The lysates were then centrifuged at 10,000 rpm for 1 min. The supernatant obtained from this step constitutes the cytoplasmic extract. The pellet was resuspended in 4× loading dye (100 mM Tris-HCl, 4% SDS, 20% glycerol, 0.2% bromophenol) and heated at 95 °C for 20–30 min.

### Proximity ligation assay

B16 cells at 50%–60% confluency were transfected with siRNA using a TurboFect transfection reagent at 1:3. 1 μM Melanocyte Stimulating Hormone was added 24 h post-transfection. At 72 h after transfection, cells grown on a coverslip were fixed using 4% Paraformaldehyde (PFA) at room temperature for 15 min. The cells were then washed with 1× PBS and permeabilized using 0.3% TritonX-100 for 15 min at room temperature. To investigate ER-lysosome interactions, primary antibodies against VAP-A (ER) (Santa Cruz Biotechnology, sc-293278) and LAMP1 (lysosomes) (Cell signaling Technology, 9091) were used. Incubations with Duolink PLA probe anti-rabbit PLUS (Sigma, DUO92002) and anti-mouse MINUS (Sigma, DUO92004), ligase and polymerase (Sigma, DUO92013), and the washes between each step were precisely as recommended by the manufacturer’s protocol. Cells were then mounted in SlowFade Gold Antifade Mountant with DAPI (Invitrogen, S36938) to label the nucleus. PLA products were visualized using the Zeiss microscope with 63×, and spots were quantified using Fiji (Image J) software, with background signals subtracted from each acquired images during quantitative analysis.

### ADP/ATP measurement assay

B16 cells at 50%–60% confluency were transfected with siRNA using a TurboFect transfection reagent at 1:3. 1 μM Melanocyte Stimulating Hormone was added 24 h post-transfection. Seventy-two h post-transfection, cells were trypsinized and followed the manufacturer’s protocol (Sigma, MAK135). Data was acquired using a Luminometer and ratio was calculated according to the manufacturer’s protocol.

### Transmission Electron Microscopy (TEM)

Cells were collected and resuspended in Phosphate buffer (PB). Remove PB buffer by centrifugation, and cells were resuspended in TEM fixative for 2 h at 4 ℃. Remove the TEM fixative by centrifugation and resuspend it in the PB buffer. Samples were processed, and ultrathin sections were sliced at around 60–90 nm. The double staining method uses uranyl acetate and alkaline lead citrate to obtain a good contrast of sections [90]. TEM images captured in JEM1400 Flash equipped with tungsten filament as an electron source and a highly sensitive sCMOS camera. Images captured at a magnification of 30,000X and a high voltage of 80kV.

### Morpholino design and microinjections

ATG-blocking antisense morpholino oligos were designed against zebrafish IP_3_R2 gene according to manufactures protocol (GENE TOOLS) (Table 4).

**Table 4:** Details of morpholinos used.

Gene name	Sequence	Company
Scrambled morpholino	CCTCTTACCTCAGTTACAATTTATA	GENE TOOLS
IP_3_R2 morpholino	CTGTGTTCCGACATTTCTGCAGGTT	GENE TOOLS

### Overexpression and complementation/rescue assay of IP_3_R2 in zebrafish

Plasmids were linearized using Not1 and then used as a template for generating an In-vitro transcript using the mMESSAGE mMACHINE T7 Transcription Kit according to the manufacturer’s protocol.

### MLSA1 treatment in zebrafish

Zebrafish embryos were treated with MLSA1 at a final concentration of 5 µM at two developmental time points, 8hpf and 33hpf. After treatment, the embryos were kept in an incubator at 28 °C under standard culture conditions. Pigmentation phenotypes were examined and documented at 48 hpf using bright-field microscopy. After phenotypic assessment, embryos were collected, and stored at −20 °C for subsequent molecular analysis.

### Statistical analysis

All experiments were performed at least 3 times. All statistical analysis was performed using Graph pad prism8.0 software. Data are represented as mean ± SEM. An unpaired Student *t* test or one-sample *t* test was performed to check statistical significance between 2 groups. A one-way ANOVA test was performed to compare the mean of more than 2 groups. Two-way ANOVA was performed to compare the mean between two groups at different time points. p-value < 0.05 was considered as significant and presented as ‘’*’’, *p*-value < 0.01 is presented as “**”, *p*-value < 0.001 is presented as “***” and *p*-value < 0.0001 is presented as “****”.

## Supporting information

S1 Data All numerical values underlying the Figs 1–9. (XLSX)

S2 Data All numerical values underlying the S1–S11 Figs. (XLSX)

S1 Raw Images Raw images of Figs 3A, 3C, 3E, 3G, 3I, 3K, 3O, 5L, 8C, S1E, S1G, S1K, S1M, S1Q, S2E, S2G, S2I, S2K, S3A, S4C, S7A, S7C, S7E and S7G. (PDF)

S1 Fig Supporting main Fig 1. IP_3_R2 positively regulates pigmentation. **(A)** Pictorial representation of The Human Protein Atlas showing IP_3_Rs protein expression in melanocytes. Created in BioRender. Motiani, R. (2026) https://BioRender.com/t2dov3u. **(B)** qRT-PCR analysis showing expression of IP_3_Rs isoform after 72 h of siRNA-mediated IP_3_R1 silencing in LD model system (*N* = 3). **(C)** qRT-PCR analysis showing expression of IP_3_Rs isoform after 72 h of siRNA-mediated IP_3_R2 silencing in LD model system (*N* = 3). **(D)** qRT-PCR analysis showing expression of IP_3_Rs isoform after 72 h of siRNA-mediated IP_3_R3 silencing in LD model system (*N* = 3). **(E)** Representative image of immunoblot showing expression of IP_3_R2 in B16 cells after siRNA silencing of IP_3_R2 in LD model system. **(F)** Bar graph showing the densitometry of IP_3_R2 band normalized to β-Tubulin (*N* = 3). **(G)** Representative image of immunoblot showing expression of IP_3_R2 in B16 cells after stable knockdown of IP_3_R2 in LD model system. **(H)** Bar graph showing the densitometry of IP_3_R2 band normalized to β-Tubulin (*N* = 3). **(I)** Representative image of pellet pictures shows B16 cells having stable knockdown of IP_3_R2 in LD model system. **(J)** Bar graph showing mean pixel intensity of B16 cells having stable knockdown of IP_3_R2 in LD model system (*N* = 3). **(K)** Representative image of immunoblot showing the expression of IP_3_R2 in B16 cells after 72 h of siRNA silencing of IP_3_R2, with a 48 h exposure to 1 μM of αMSH. **(L)** Bar graph showing the densitometry of the IP_3_R2 band normalized to β-Tubulin (*N* = 3). **(M)** Representative image of immunoblot showing expression of IP_3_R2 in B16 cells after stable knockdown of IP_3_R2 with 48 h exposure to 1 μM of αMSH. **(N)** Bar graph showing the densitometry of IP_3_R2 band normalized to β-Tubulin (*N* = 3). **(O)** Representative image of pellet pictures shows B16 cells having stable knockdown of IP_3_R2 along with adding 1 µM αMSH. **(P)** Bar graph showing mean pixel intensity of B16 cells having stable knockdown of IP_3_R2 along with adding 1 µM αMSH (*N* = 3). **(Q)** Representative image of immunoblot showing the expression of IP_3_R2 in lightly pigmented primary human melanocytes after 72 h of IP_3_R2 silencing. **(R)** Bar graph showing the densitometry of the IP_3_R2 band normalized to β-Tubulin (*N* = 3). **(S)** qRT-PCR analysis showing expression of IP_3_Rs isoform after 72 h of overexpression with functional IP_3_R2 in LD model system (*N* = 3). **(T)** qRT-PCR analysis showing expression of IP_3_Rs isoform after 72 h of overexpression with functional IP_3_R2-M in LD model system (*N* = 4). Data presented are mean ± SEM. For statistical analysis, one-sample *t* test was performed for panels F, H, J, L, N, P, R and Dunnett’s multiple comparisons test was performed for panels B, C, D, S, T using GraphPad Prism software. Here, ‘ns’ means non-significant; * *p* < 0.05, ** *p* < 0.01, *** *p* < 0.001and **** *p* < 0.0001.
(TIF)

S2 Fig Supporting main Fig 3. IP_3_R2 knockdown increases the stability of melanogenic proteins. **(A)** qRT-PCR analysis showing expression of IP_3_R2 in B16 cells incubated with 1 μM αMSH for 12, 24, and 48 h following 24 h of transfection with siNT or siIP_3_R2 (*N* = 3). **(B)** qRT-PCR analysis showing expression of GP100 in B16 cells incubated with 1 μM αMSH for 12, 24, and 48 h following 24 h of transfection with siNT or siIP_3_R2 (*N* = 3). **(C)** qRT-PCR analysis showing expression of Tyrosinase in B16 cells incubated with 1 μM αMSH for 12, 24, and 48 h following 24 h of transfection with siNT or siIP_3_R2 (*N* = 3). **(D)** qRT-PCR analysis showing expression of DCT in B16 cells incubated with 1 μM αMSH for 12, 24, and 48 h following 24 h of transfection with siNT or siIP_3_R2 (*N* = 3). **(E)** Representative image of immunoblot showing expression of GP100 in B16 cells after IP_3_R2 silencing. **(F)** Bar graph showing the densitometry of GP100 band normalized to β-Tubulin (*N* = 3). **(G)** Representative image of immunoblot showing expression of Tyrosinase in B16 cells after IP_3_R2 silencing. **(H)** Bar graph showing the densitometry of Tyrosinase band normalized to β-Tubulin (*N* = 3). **(I)** Representative image of immunoblot showing expression of DCT in B16 cells after IP_3_R2 silencing. **(J)** Bar graph showing the densitometry of DCT band normalized to β-Tubulin (*N* = 3). **(K)** Representative image of immunoblot showing expression of the IP_3_R2 and the melanogenic enzymes DCT, Tyrosinase, and DCT in B16 cells for different time interval of cycloheximide treatment after siRNA transfections. **(L)** The line graph shows the percentage of the proteins remaining relative to 0 h of cycloheximide treatment by densitometry of the IP_3_R2 band normalized to β-Tubulin. **(M)** The line graph shows the percentage of the proteins remaining relative to 0 h of cycloheximide treatment by densitometry of the GP100 band normalized to β-Tubulin. **(N)** The line graph shows the percentage of the proteins remaining relative to 0 h of cycloheximide treatment by densitometry of the Tyrosinase band normalized to β-Tubulin. **(O)** The line graph shows the percentage of the proteins remaining relative to 0 h of cycloheximide treatment by densitometry of the DCT band normalized to β-Tubulin. Data presented are mean ± SEM. For statistical analysis, Sidak’s multiple comparisons test was performed for panels A, B, C, D, and one-sample *t* test was performed for panels F, H, J using GraphPad Prism software. Here, ‘ns’ means non-significant; * *p* < 0.05, and **** *p* < 0.0001.
(TIF)

S3 Fig Supporting main Figs 3 and 4. **Functional validation of melanophagy probes. (A)** Representative image of immunoblot showing expression of LC3II and LC3I in B16 cells after siRNA silencing of IP_3_R2 in LD model system. **(B)** Bar graph showing the densitometric analysis of the LC3II/LC3I ratio (*N* = 4). **(C)** Representative images of confocal imaging in B16 cells stimulated with 4 µM rapamycin and 100 nM BafilomycinA1 for 5 h after transfection with mCherry-Tyrosinase-EGFP probe, scale bar, 5 µm. **(D)** Bar graph shows the quantification of the autolysosome intensity in the EGFP:mCherry ratio, where ‘*N*’ denotes the number of cells. **(E)** Representative images of confocal imaging in B16 cells showing co-localization between HMB45 and mCherry-Tyrosinase-EGFP probe, scale bar, 10 µm. **(F)** Bar graph shows the PCC and OC between HMB45 and mCherry of the mCherry-Tyrosinase-EGFP probe, where ‘*N*’ denotes the number of images analyzed. **(G)** Representative images of confocal imaging in B16 cells stimulated with 10 µM RTG for 24 h after transfection with mCherry-Tyrosinase-EGFP probe, scale bar, 5 µm. **(H)** Bar graph shows the quantification of the autolysosome intensity in the EGFP:mCherry ratio, where ‘*N*’ denotes the number of cells. **(I)** Representative images of confocal imaging in B16 cells stimulated with 4 µM rapamycin and 100 nM BafilomycinA1 for 5 h after transfection with Tyrosinase-mKeimaN1 probe, scale bar, 10 µm. **(J)** Bar graph shows the quantification of the autolysosome intensity in the 543:488 nm ratio, which is alternatively excited at 543 nm and 488 nm, where ‘*N*’ denotes the number of cells. **(K)** Representative images of confocal imaging in B16 cells showing co-localization between HMB45 and Tyrosinase-mKeimaN1 probe, scale bar, 5 µm. **(L)** Bar graph shows the PCC and OC between HMB45 and 543 nm of the Tyrosinase-mKeimaN1 probe, where ‘*N*’ denotes the number of images analyzed. Data presented are mean ± SEM. For statistical analysis, unpaired *t* test was performed for panel H, one-sample *t* test was performed for panel B, and Dunnett’s multiple comparisons test was performed for panels D, J using GraphPad Prism software. Here, ** *p* < 0.01, *** *p* < 0.001 and **** *p* < 0.0001.
(TIF)

S4 Fig Supporting main Fig 4. IP_3_R2 silencing increases melanosome turnover. **(A)** Representative images of confocal imaging in B16 cells demonstrates co-localization between HMB45 and LC3(A/B) upon IP_3_R2 silencing, scale bar, 5 µm. **(B)** Bar graph shows the PCC between HMB45 and LC3(A/B), where ‘*N*’ denotes the number of images. **(C)** Representative image of immunoblot showing expression of Tyrosinase in B16 cells after siRNA transfection including 6 h of treatment with 100 nM of the lysosomal inhibitor Bafilomycin A1. **(D)** Bar graph showing the densitometry of the Tyrosinase band normalized to β-Tubulin (*N* = 4). Data presented are mean ± SEM. For statistical analysis, unpaired *t* test was performed for panel B, and Tukey’s multiple comparisons test was performed for panel D, using GraphPad Prism software. Here, ‘ns’ means non-significant; * *p* < 0.05, ** *p* < 0.01 and **** *p* < 0.0001.
(TIF)

S5 Fig Supporting main Fig 4. IP_3_R2 silencing leads to selective melanosome degradation. **(A)** Representative TEM image of stage III or IV melanosome showing B16 cells transfected with siNT or siIP_3_R2, scale bar, 500 nm. **(B)** Bar graph shows the quantification of the melanosome number, where ‘*N*’ denotes the melanosome number per image. **(C)** Representative TEM image showing B16 cells having stable IP_3_R2 knockdown demonstrating rescue with IP_3_R2 and IP_3_R2-M, scale bar, 500 nm. **(D)** Bar graph shows the quantification of the melanosome number, where ‘*N*’ denotes the melanosome number per image. **(E)** Representative images of confocal imaging in B16 cells treated with 10 µM FCCP for 8 h or transfected with siNT or siIP_3_R2 along with mKeima-Red-Mito-7 construct, scale bar, 5 µm. **(F)** Bar graph shows the quantification of the autolysosome intensity in the 543 nm:488 nm ratio, which is alternatively excited at 543 nm and 488 nm, where ‘*N*’ denotes the number of cells. **(G)** Representative images of confocal imaging in B16 cells treated with 1mM 4-PBA for 48 h or transfected with siNT or siIP_3_R2 along with GST-Keima-cb5 construct, scale bar, 5 µm. **(H)** Bar graph shows the quantification of the autolysosome intensity in the 543 nm:488 nm ratio, which is alternatively excited at 543 nm and 488 nm, where ‘*N*’ denotes the number of cells. Data presented are mean ± SEM. For statistical analysis, unpaired *t* test was performed for panels B, F, H and Dunnett’s multiple comparisons test was performed for panel D, using GraphPad Prism software. Here, ‘ns’ means non-significant; ** *p* < 0.01, *** *p* < 0.001and **** *p* < 0.0001.
(TIF)

S6 Fig Supporting main Fig 5. IP_3_R2 specifically restores mitochondrial Ca^2+^ uptake in IP_3_R2-deficient melanocytes. **(A)** Representative mitochondrial Ca^2+^ imaging trace using the Cepia2mt probe in B16 cells having stable IP_3_R2 knockdown upon stimulation with Histamine. **(B)** Bar graph showing the quantification of Ca^2+^ imaging traces stimulated with 50 µM histamine, where ‘*N*’ denotes the total number of ROI in that trace. **(C)** Representative mitochondrial Ca^2+^ imaging trace using the Cepia2mt probe in B16 cells having stable IP_3_R2 knockdown with overexpression of either one of IP_3_R1 or IP_3_R2 or IP_3_R3, stimulated with Histamine. **(D)** Bar graph showing the quantification of Ca^2+^ imaging traces stimulated with 50 µM histamine, where ‘*N*’ denotes the total number of ROI in that trace. Data presented are mean ± SEM. For statistical analysis, an unpaired *t* test was performed for panel B, and Tukey’s multiple comparisons test was performed for panel D, using GraphPad Prism software. Here, ‘ns’ means non-significant; ** *p* < 0.01, and **** *p* < 0.0001.
(TIF)

S7 Fig Supporting main Fig 5. IP_3_R2 knockdown-induced melanophagy is mTOR independent. **(A)** Representative image of immunoblot showing expression of p-ULK1 and total ULK1 in B16 cells after siRNA silencing of IP_3_R2. **(B)** Bar graph showing the densitometric analysis of the relative changes in p-ULK1 to total ULK1 (*N* = 3). **(C)** Representative image of immunoblot showing expression of p-mTOR and total mTOR in B16 cells after siRNA silencing of IP_3_R2. **(D)** Bar graph showing the densitometric analysis of the relative changes in the levels of p-mTOR to total mTOR (*N* = 3). **(E)** Representative image of immunoblot showing expression of p-70S6K and total 70S6K in B16 cells after siRNA silencing of IP_3_R2. **(F)** Bar graph showing the densitometric analysis of the relative changes in the levels p-70S6K to total 70S6K (*N* = 3). **(G)** Representative image of immunoblot showing expression of p-4EBP1 and total 4EBP1 in B16 cells after siRNA silencing of IP_3_R2. **(H)** Bar graph showing the densitometric analysis of the relative changes in the p-4EBP1 to total 4EBP1 (*N* = 3). Data presented are mean ± SEM. For statistical analysis, one-sample *t* test was performed for panels B, D, F and H, using GraphPad Prism software. Here, ‘ns’ means non-significant; ** *p* < 0.01.
(TIF)

S8 Fig Supporting main Fig 6. Decreased lysosomal pH accompanied by elevated lysosomal Ca^2+^ levels. **(A)** Representative images of confocal imaging in B16 cells after transfected with siNT or siIP_3_R2 along with LAMP1-RpHLuorin2 construct, scale bar, 10 µm. **(B)** Bar graph showing the quantification of the intraluminal lysosomal pH, in the 405 nm:488 nm ratio, which is alternatively excited at 405 nm and 488 nm, where ‘*N*’ denotes the number of cells. **(C)** Representative images of confocal imaging in B16 cells loaded with LysoSensor Yellow/Blue DND-160 dye for 5 min in lightly pigmented and darkly pigmented primary melanocytes, scale bar, 10 µm. **(D)** Bar graph showing the quantification of the intraluminal lysosomal pH in Yellow/Blue ratio, where ‘*N*’ denotes the number of cells imaged. **(E)** qRT-PCR analysis showing expression of ATP6V0D1 after 36 h of siRNA-mediated IP_3_R2 silencing accompanied with 12 h of αMSH treatment (*N* = 4). **(F)** qRT-PCR analysis showing expression of ATP6V1H after 36 h of siRNA-mediated IP_3_R2 silencing accompanied with 12 h of αMSH treatment (*N* = 3). **(G)** Representative images of confocal imaging in B16 cells loaded with OG-BAPTA-dextran (OBDx) in lightly pigmented and darkly pigmented primary melanocytes, scale bar, 10 µm. **(H)** Bar graph showing the quantification of the Green intensity, where ‘*N*’ denotes the number of cells imaged. **(I)** Representative traces of Fura2AM-based Ca^2+^ imaging in B16 cells stimulated with the Tg followed by Bafilomycin A1 after 72 h of siRNA transfection. **(J)** Bar graph showing the quantification of Ca^2+^ imaging traces stimulated with 2 µM Tg, where ‘*N*’ denotes the total number of cells imaged. **(K)** Bar graph showing the quantification of Ca^2+^ imaging traces stimulated with 1 µM Bafilomycin A1, where ‘*N*’ denotes the total number of cells imaged. **(L)** Representative traces of Fura2AM-based Ca^2+^ imaging in B16 cells stimulated with the Tg followed by GPN after 72 h of siRNA transfection. **(M)** Bar graph showing the quantification of Ca^2+^ imaging traces stimulated with 2 µM Tg, where ‘*N*’ denotes the total number of cells imaged. **(N)** Bar graph showing the quantification of Ca^2+^ imaging traces stimulated with 300 µM GPN, where ‘*N*’ denotes the total number of cells imaged. Data presented are mean ± SEM. For statistical analysis, an unpaired *t* test was performed for panels B, D, H, J, K, M, N, one-sample *t* test was performed for panels E and F, using GraphPad Prism software. Here, * *p* < 0.05, *** *p* < 0.001 and **** *p* < 0.0001.
(TIF)

S9 Fig Supporting main Fig 6. TMEM165 alters lysosomal Ca^2+^ homeostasis and pigmentation. **(A)** qRT-PCR analysis showing increase in TMEM165 mRNA expression after 72 h of siRNA-mediated IP_3_R2 silencing. (*N* = 3). **(B)** qRT-PCR analysis showing decrease expression of TMEM165 after 72 h of siRNA-mediated TMEM165 silencing. **(C)** Representative images of confocal imaging in B16 cells loaded with OG-BAPTA-dextran (OBDx) after transfected with siNT or siTMEM165, scale bar, 5 µm. **(D)** Bar graph showing the quantification of the Green intensity, where ‘*N*’ denotes the number of cells imaged. **(E)** Representative image of pellet pictures shows B16 cells transfected with siNT or siTMEM165. **(F)** Bar graph showing mean pixel intensity of B16 cells transfected with siNT or siTMEM165 (*N* = 3). **(G)** Representative images of confocal imaging in B16 cells transfected with siNT or siTMEM165 along with mCherry-Tyrosinase-EGFP probe, scale bar, 5 µm. **(H)** Bar graph shows the quantification of the autolysosome intensity in the EGFP:mCherry ratio, where ‘*N*’ denotes the number of cells. **(I)** Representative images of confocal imaging in B16 cells loaded with OG-BAPTA-dextran (OBDx) after transfection with siRNA, scale bar, 5 µm. **(J)** Bar graph showing the quantification of the Green intensity, where ‘*N*’ denotes the number of cells imaged. **(K)** Representative images of confocal imaging in B16 cells transfected with siRNA along with mCherry-Tyrosinase-EGFP probe, scale bar, 5 µm. **(L)** Bar graph shows the quantification of the autolysosome intensity in the EGFP:mCherry ratio, where ‘*N*’ denotes the number of cells. **(M)** Representative images of confocal imaging in B16 cells loaded with OG-BAPTA-dextran (OBDx) after overexpressing with pcDNA or TMEM165 OE plasmid, scale bar, 5 µm. **(N)** Bar graph showing the quantification of the Green intensity, where ‘*N*’ denotes the number of cells imaged. **(O)** Representative images of confocal imaging in B16 cells overexpressing with pcDNA or TMEM165 OE plasmid along with mCherry-Tyrosinase-EGFP probe, scale bar, 5 µm. **(P)** Bar graph shows the quantification of the autolysosome intensity in the EGFP:mCherry ratio, where ‘*N*’ denotes the number of cells. Data presented are mean ± SEM. For statistical analysis, an unpaired *t* test was performed for panels D, H, N, P one-sample *t* test was performed for panels A, B, F and Tukey’s multiple comparisons test was performed for panels J and L, using GraphPad Prism software. Here, * *p* < 0.05, ** *p* < 0.01, *** *p* < 0.001 and **** *p* < 0.0001.
(TIF)

S10 Fig Supporting main Fig 7. IP_3_R2 knockdown enhances TRPML1 activity. **(A)** qRT-PCR analysis showing expression of TRPMLs isoform after 48 h of siRNA-mediated TRPML1 silencing (*N* = 3). **(B)** Representative Ca^2+^ imaging trace using the TRPML1-G-GECO probe in B16 cells stimulated with the MLSA1 after 72 h of siRNA transfection. **(C)** Bar graph showing the quantification of Ca^2+^ imaging traces stimulated with 20 µM MLSA1, where ‘*N*’ denotes the total number of ROI in that trace. **(D)** Representative images of confocal imaging in B16 cells loaded with LysoSensor Yellow/Blue DND-160 dye for 5 min after transfected with siNT or siTRPML1, scale bar, 5 µm. **(E)** Bar graph showing the quantification of the intraluminal lysosomal pH in Yellow/Blue ratio, where ‘*N*’ denotes the number of cells imaged. **(F)** Representative images of confocal imaging in B16 cells after transfected with siNT or siTRPML1 along with mCherry-Tyrosinase-EGFP probe, scale bar, 10 µm. **(G)** Bar graph shows the quantification of the autolysosome intensity in the EGFP:mCherry ratio, where ‘*N*’ denotes the number of cells. **(H)** Representative Ca^2+^ imaging trace using the TRPML1-G-GECO probe in B16 cells stimulated with the MLSA1 after overexpressing with TRPML1-WT and TRPML1-M. **(I)** Bar graph showing the quantification of Ca^2+^ imaging traces stimulated with 20 µM MLSA1, where ‘*N*’ denotes the total number of ROI in that trace. Data presented are mean ± SEM. For statistical analysis, Dunnett’s multiple comparisons test was performed for panels A, I, and an unpaired *t* test was performed for panels C, E, G using GraphPad Prism software. Here, ‘ns’ means non-significant; * *p* < 0.05, ** *p* < 0.01, *** *p* < 0.001and **** *p* < 0.0001.
(TIF)

S11 Fig Supporting main Fig 8. IP_3_R2 knockdown increases melanophagy flux without altering TPC2 levels. **(A)** qRT-PCR analysis showing increase in BECN1 mRNA expression after 72 h of siRNA-mediated IP_3_R2 silencing. (*N* = 3). **(B)** qRT-PCR analysis showing increase in MAP1LC3B mRNA expression after 72 h of siRNA-mediated IP_3_R2 silencing. (*N* = 4). **(C)** qRT-PCR analysis showing increase in ATG9B mRNA expression after 72 h of siRNA-mediated IP_3_R2 silencing. (*N* = 3). **(D)** qRT-PCR analysis showing increase in ATG5 mRNA expression after 72 h of siRNA-mediated IP_3_R2 silencing. (*N* = 3). **(E)** qRT-PCR analysis showing decrease in RCHY1 mRNA expression after 72 h of siRNA-mediated RCHY1 silencing. (*N* = 4). **(F)** qRT-PCR analysis showing decrease in OPTN mRNA expression after 72 h of siRNA-mediated OPTN silencing. (*N* = 4). **(G)** Representative traces of Fura2AM-based Ca^2+^ imaging in B16 cells stimulated with the TPC2-A1-N after 72 h of siRNA transfection. **(H)** Bar graph showing the quantification of Ca^2+^ imaging traces stimulated with 10 µM TPC2-A1-N, where ‘*N*’ denotes the total number of cells imaged. **(I)** Representative images of confocal imaging in B16 cells demonstrates TFEB nuclear translocation stimulated with 10 µM TPC2-A1-N for 1 h, after transfecting with siNT or siIP_3_R2 along with pEGFP-N1-TFEB construct, scale bar, 10 µm. **(J)** Bar graph showing the quantification of the EGFP intensity from the cytoplasm and nucleus in the nucleus/cytoplasmic ratio following the addition of 10 µM TPC2-A1-N for 1 h, where ‘*N*’ denotes the number of cells. Data presented are mean ± SEM. For statistical analysis, an unpaired *t* test was performed for panels H, J and one-sample *t* test was performed for panels A, B, C, D, E and F using GraphPad Prism software. Here, ‘ns’ means non-significant; * *p* < 0.05, ** *p* < 0.01, *** *p* < 0.001and **** *p* < 0.0001.
(TIF)

## Abbreviations

αMSH: α-melanocyte-stimulating hormone
AMPK: AMP-activated protein kinase
GPCR: G protein-coupled receptor
IP_3_R2: Inositol 1,4,5-trisphosphate receptor 2
LD: low-density
LROs: lysosome-related organelles
LSD: lysosomal storage disorders
MCU: Mitochondrial Ca^2+^ Uniporter
OC: Overlap Coefficient
PCC: Pearson Correlation Coefficient
PLA: proximity ligation assays
PLC: Phospholipase C
PTU: phenylthiourea
qRT-PCR: quantitative real-time PCR
siNT: siNon-targeting
siRNA: small interfering RNA
TEM: Transmission Electron Microscopy
TFEB: transcription factor EB
ULK1: Unc-51-like kinase1
UPS: ubiquitin-proteasome system
UV: ultraviolet
4EBP1: 4E-binding protein 1
