# Supplementary material for: IP3R2-mediated inter-organelle calcium signaling suppresses melanosome degradation
Source: PLoS Biol. 2026 Aug 25;24(8):e3003971. doi: 10.1371/journal.pbio.3003971 (PMC13533435; doi:10.1371/journal.pbio.3003971)

Fig 3A

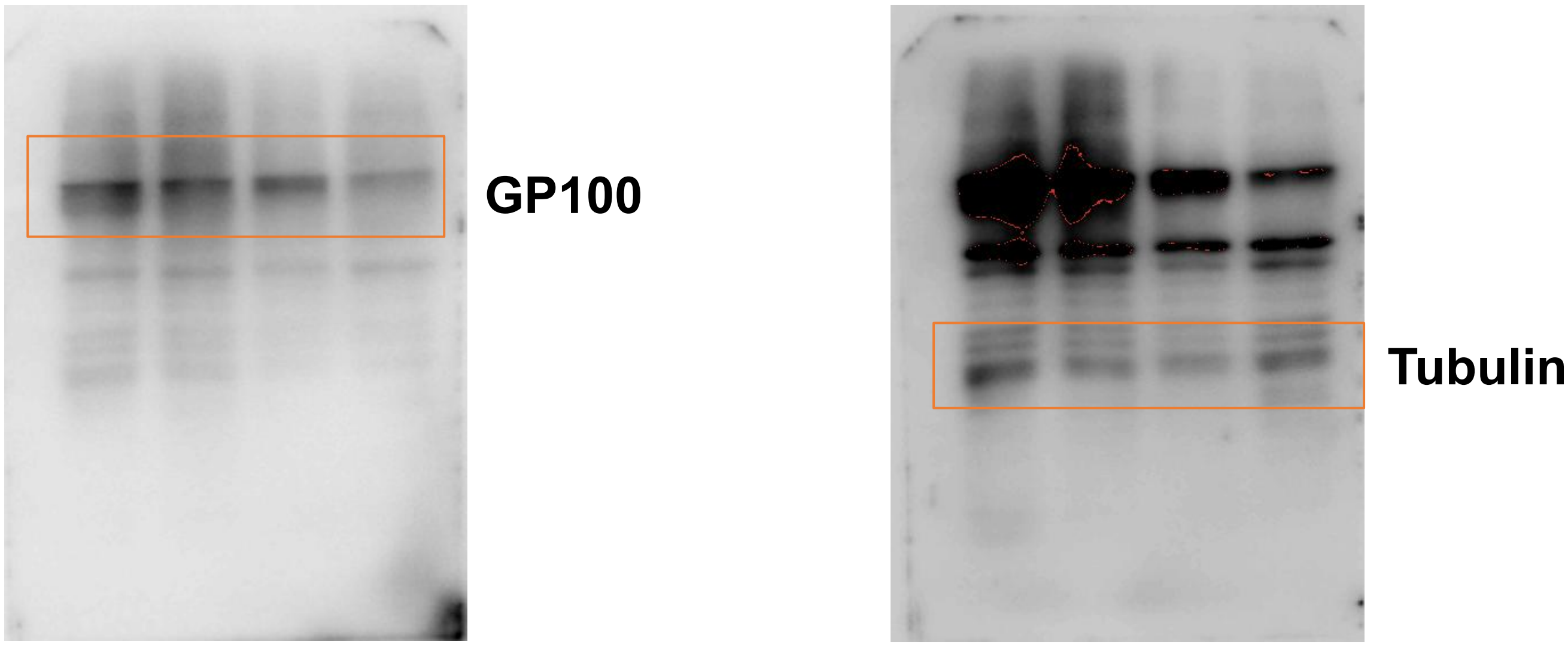

Fig 3C

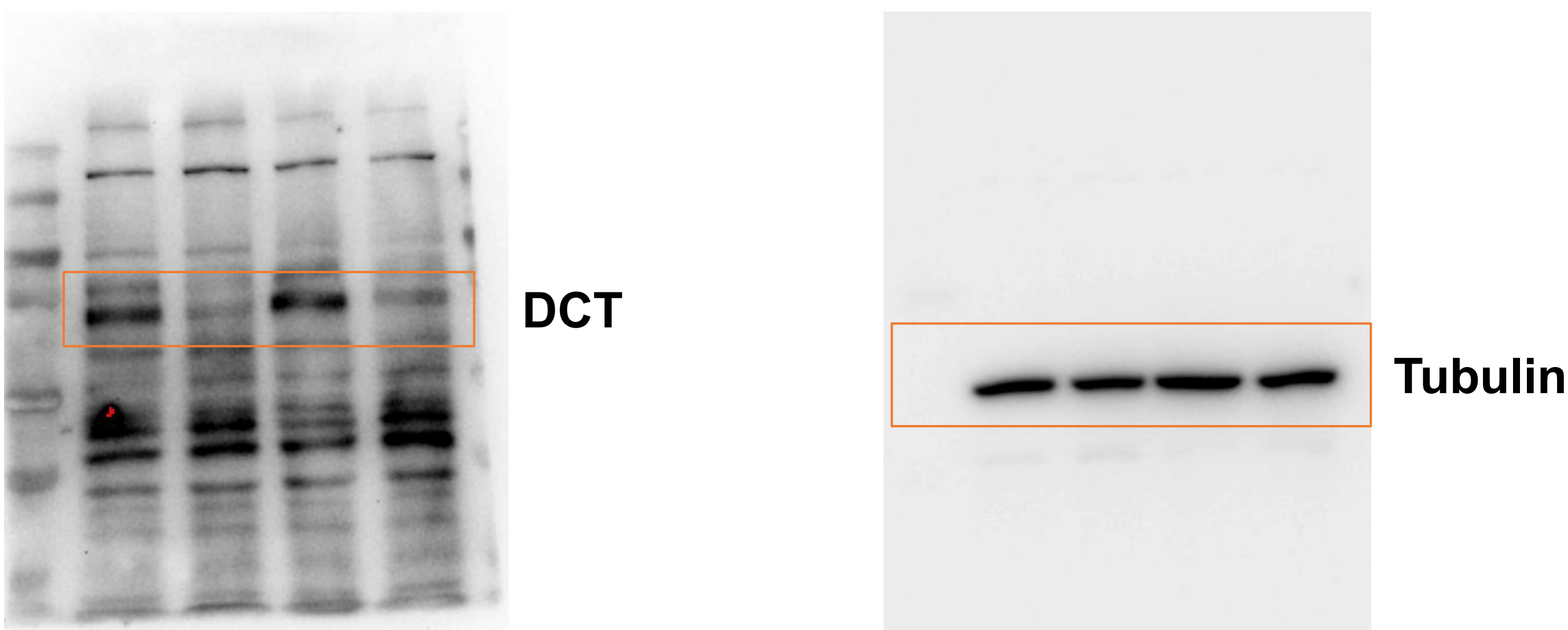

Fig 3E

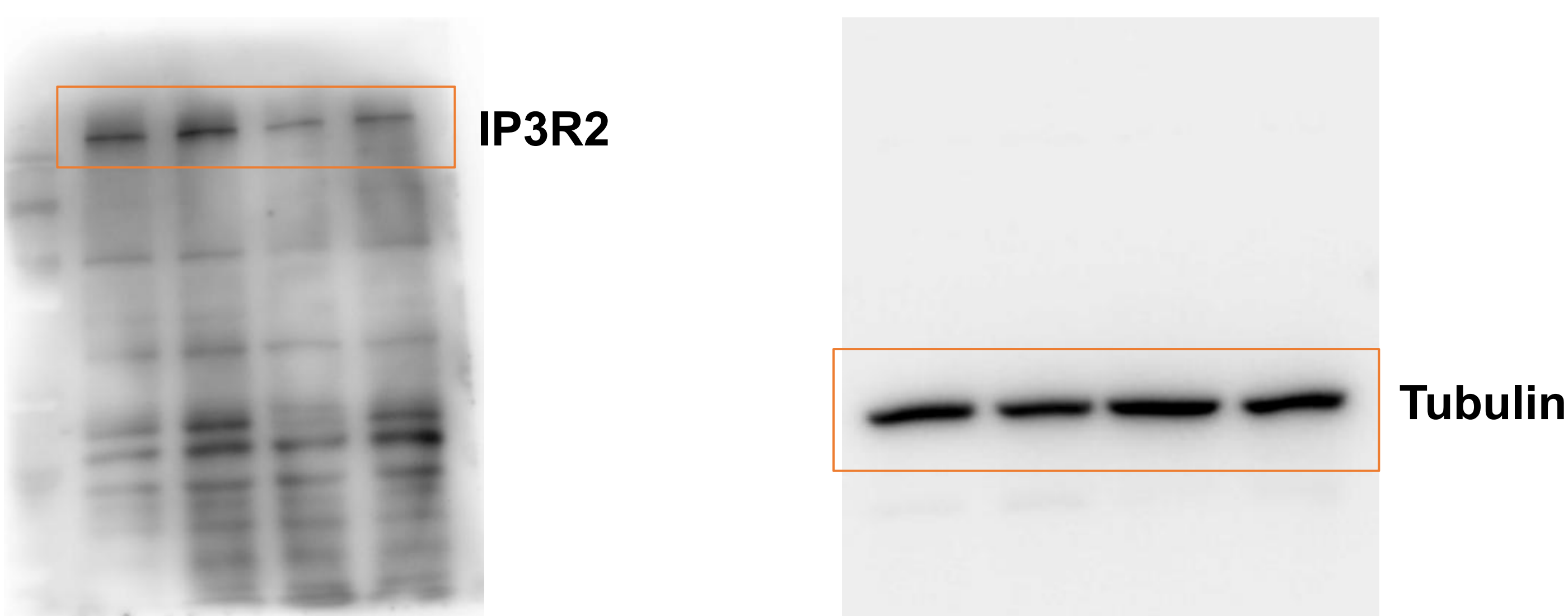

Fig 3G

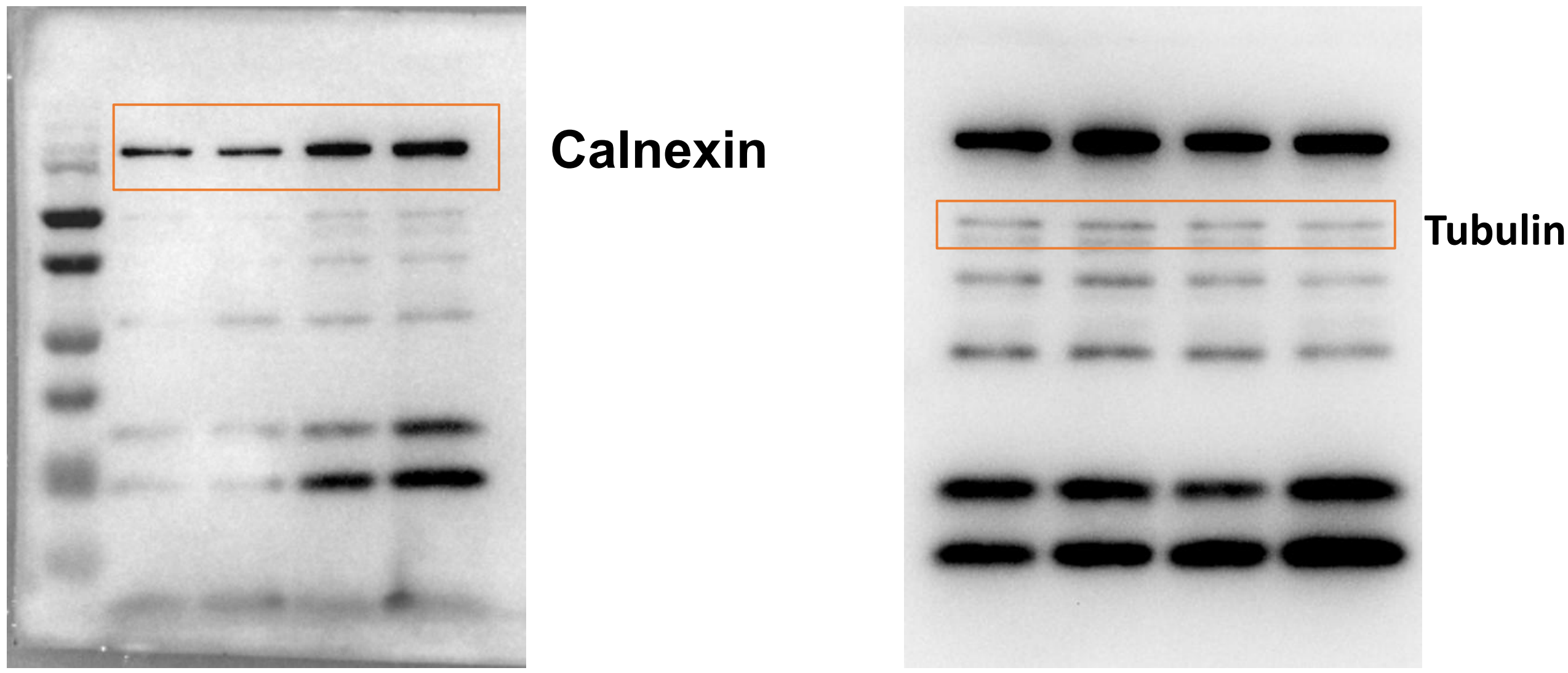

Fig 3I

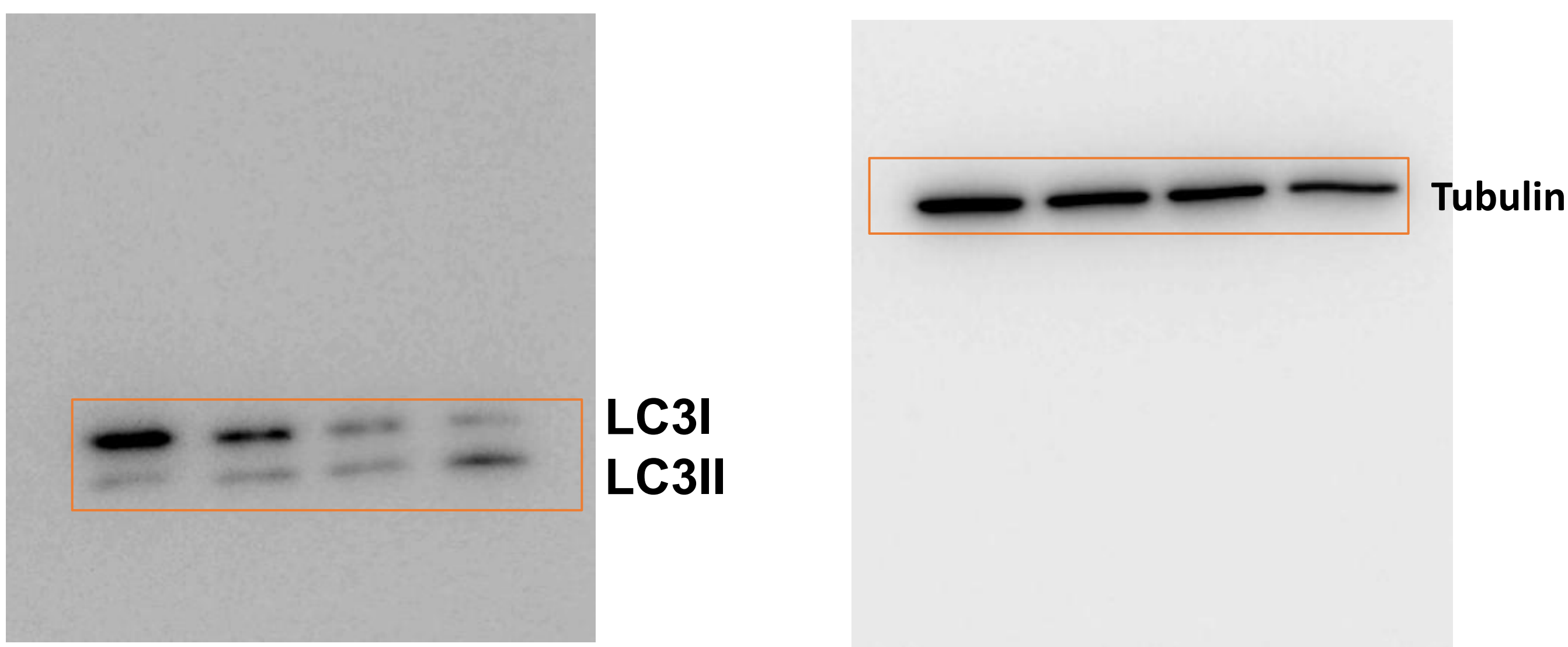

Fig 3K

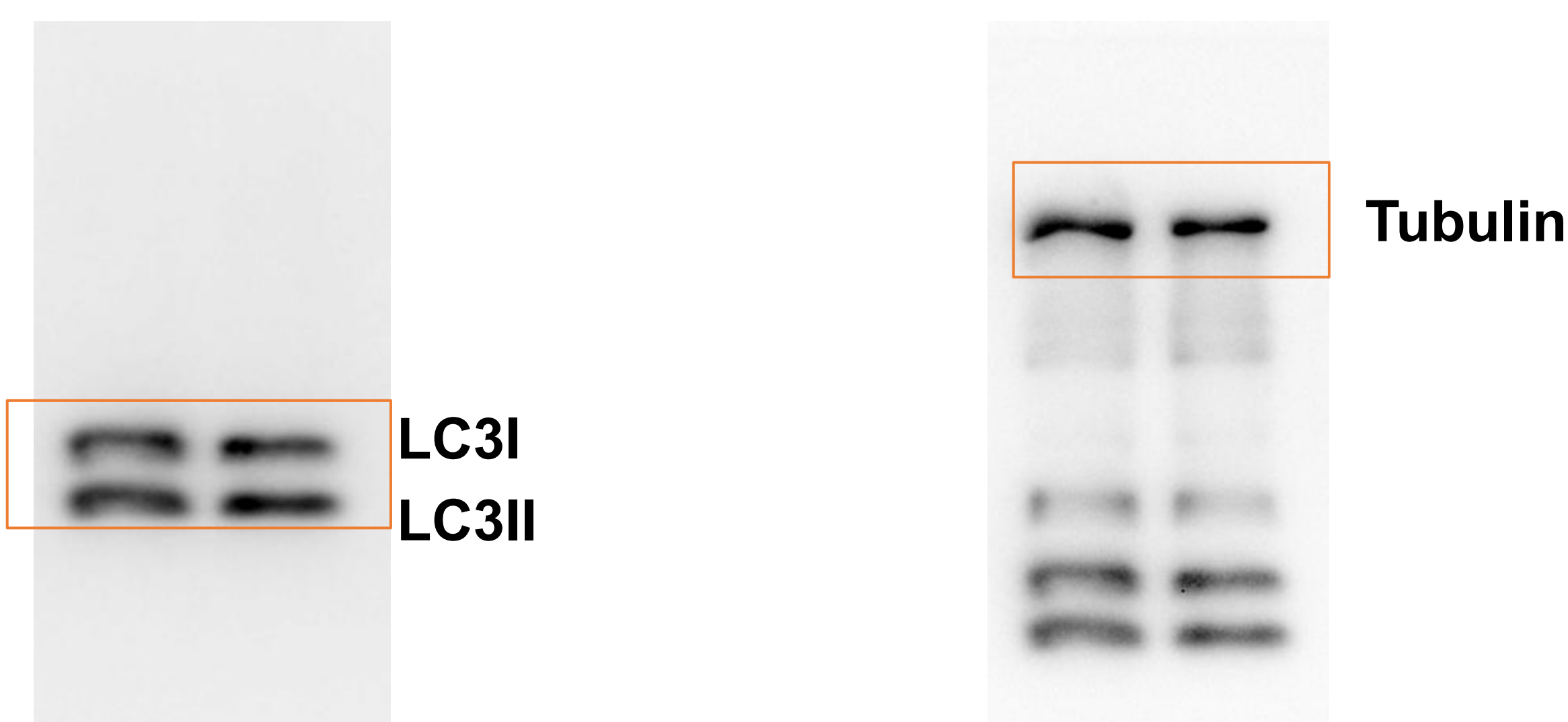

Fig 3O

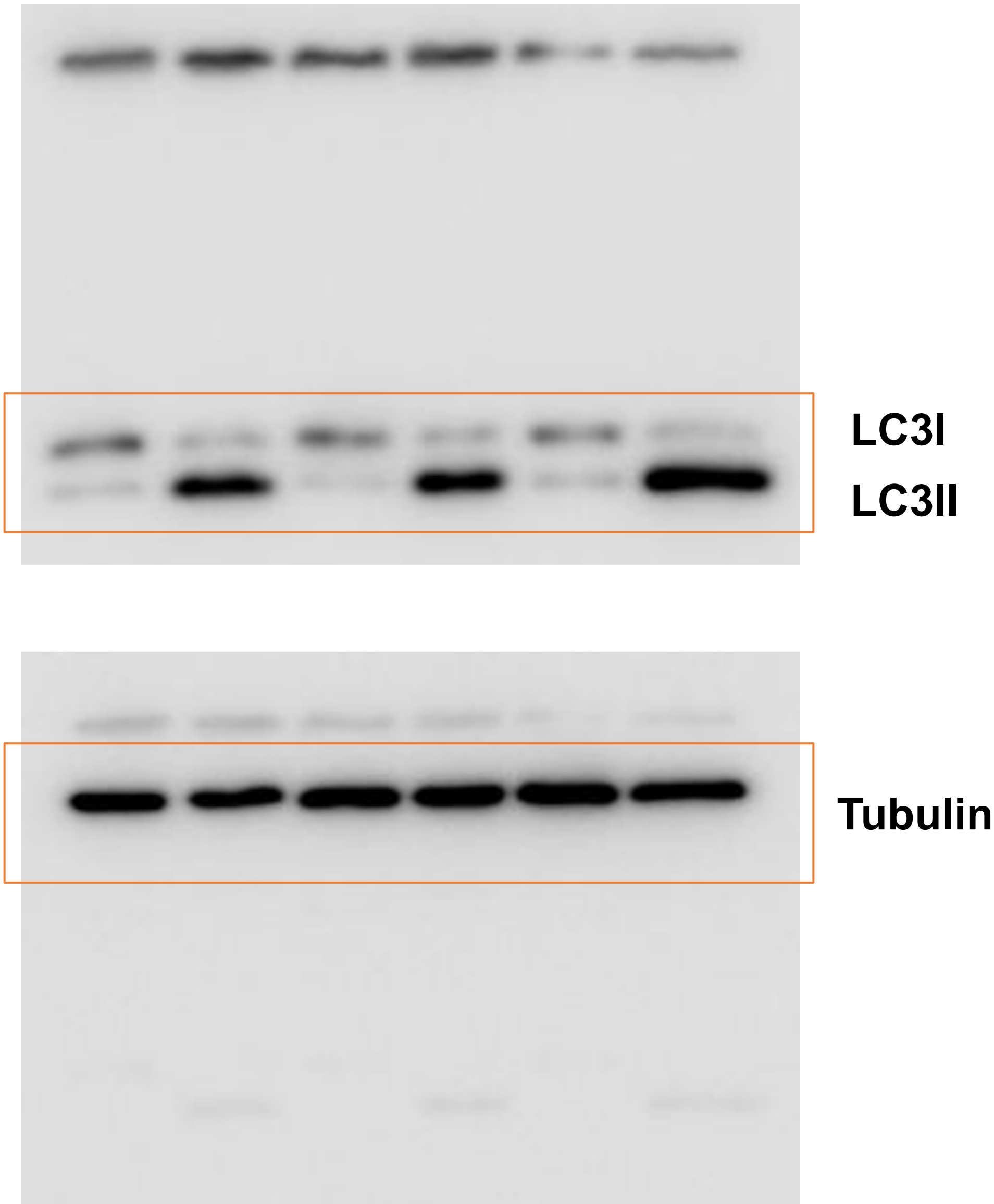

Fig 5L

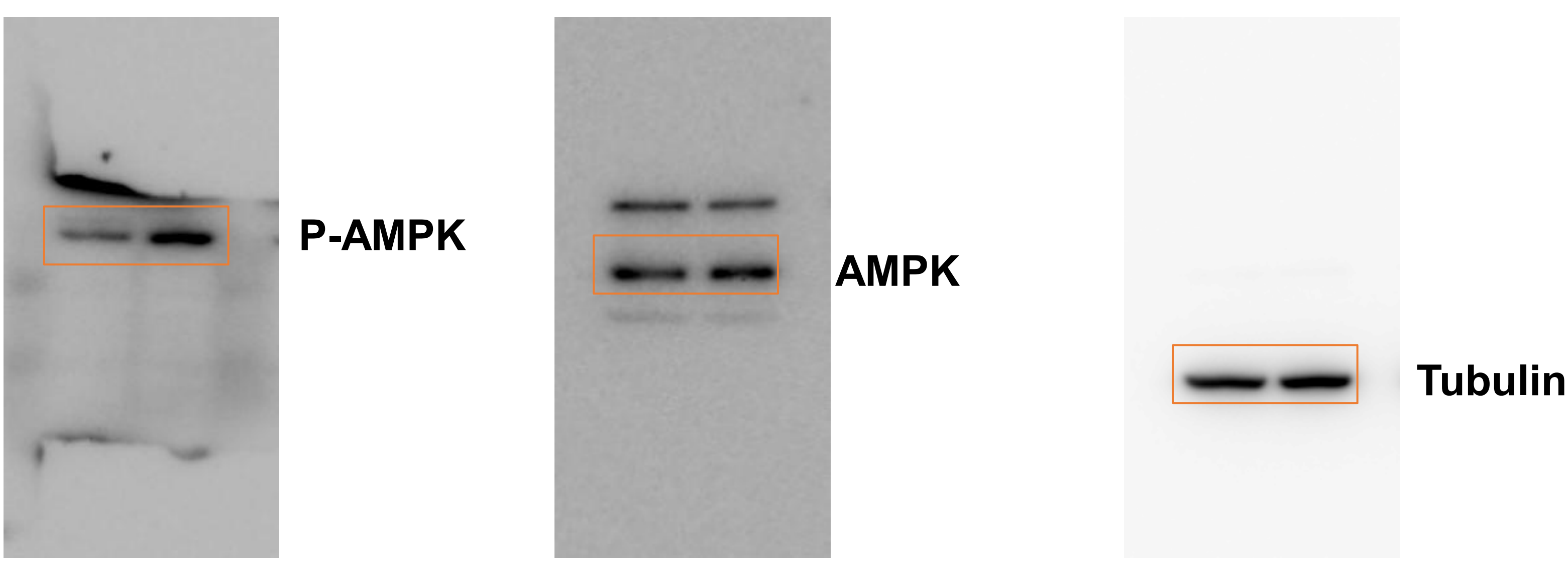

Fig 8C

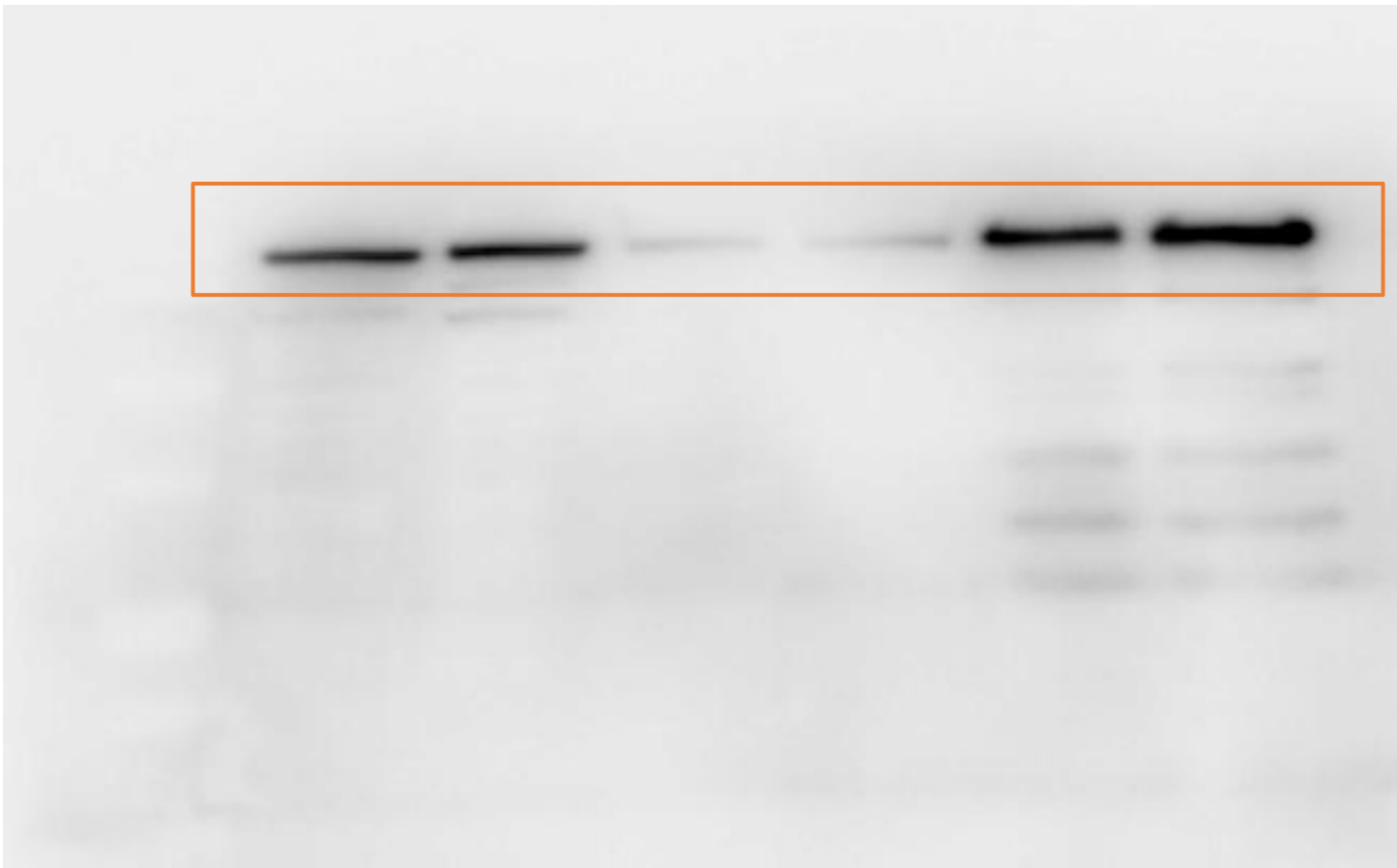

TFEB

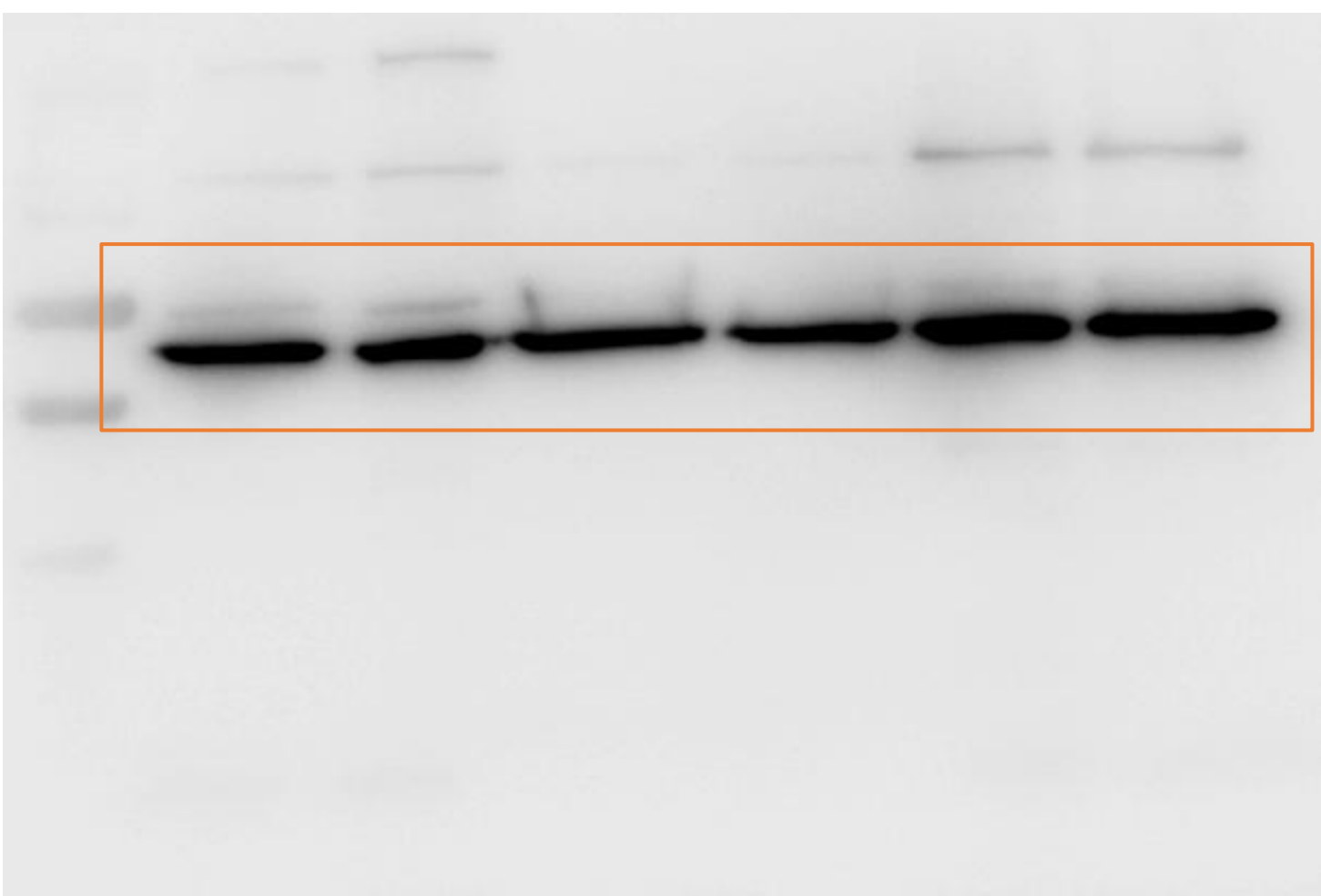

Actin

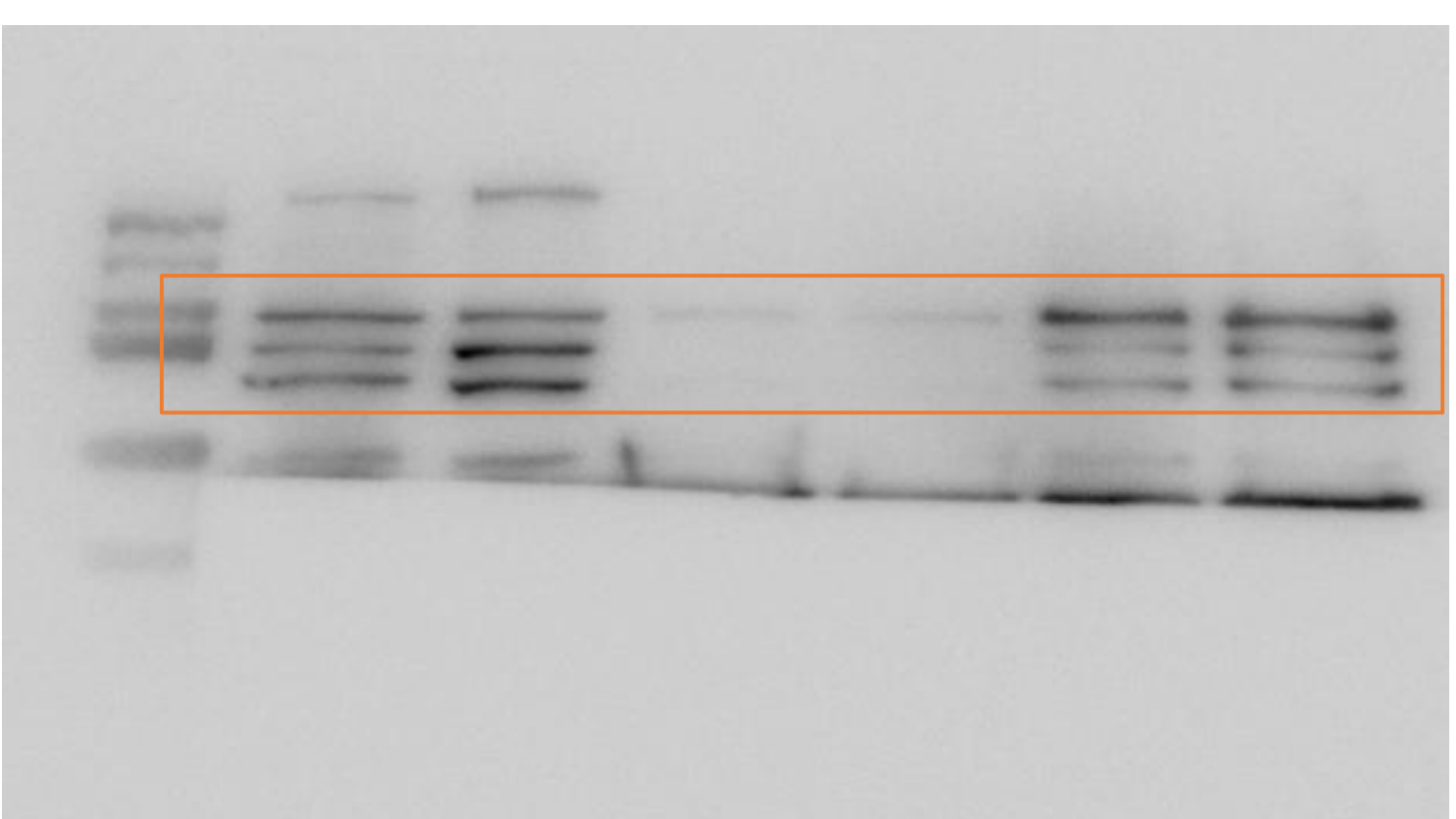

Lamin

S1E Fig

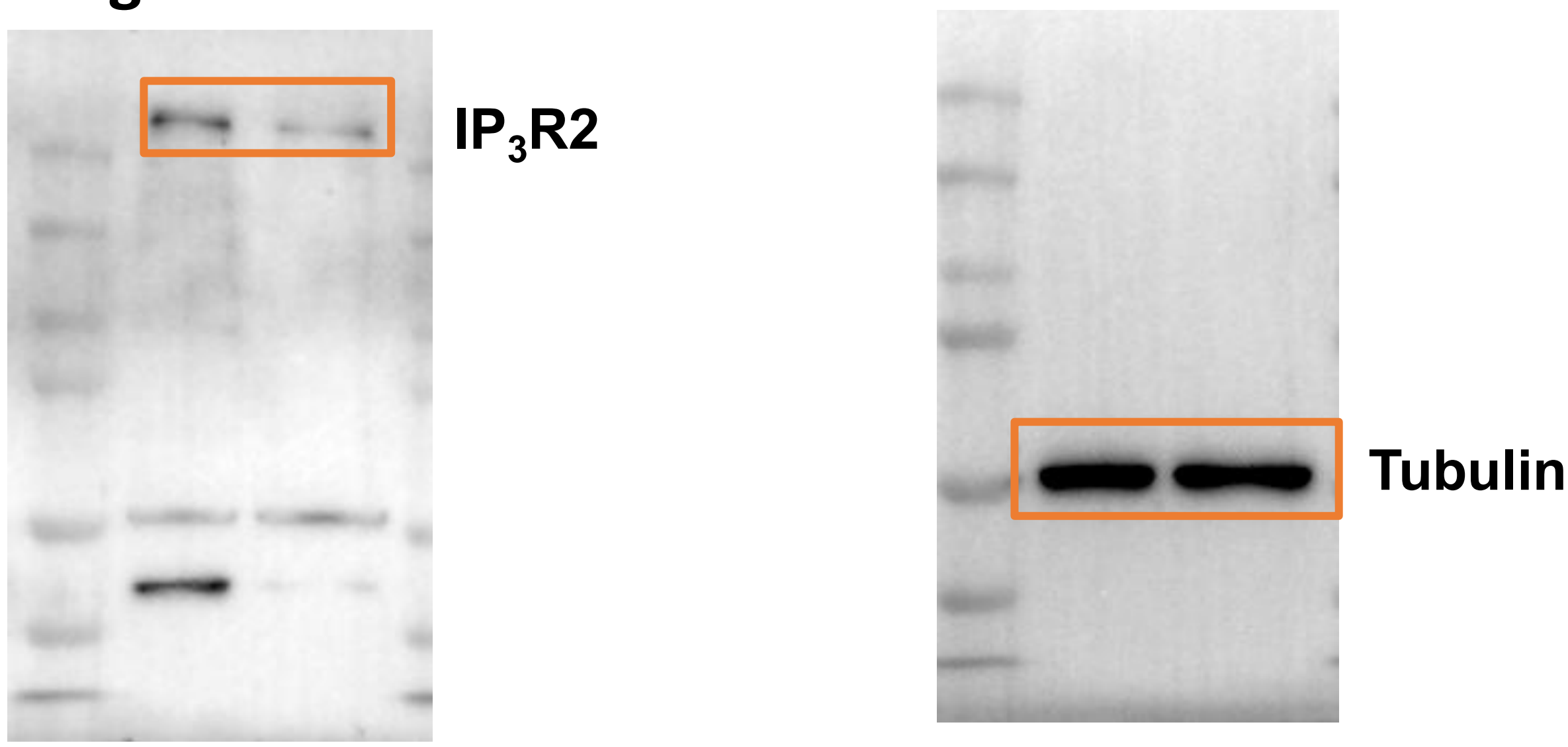

S1G Fig

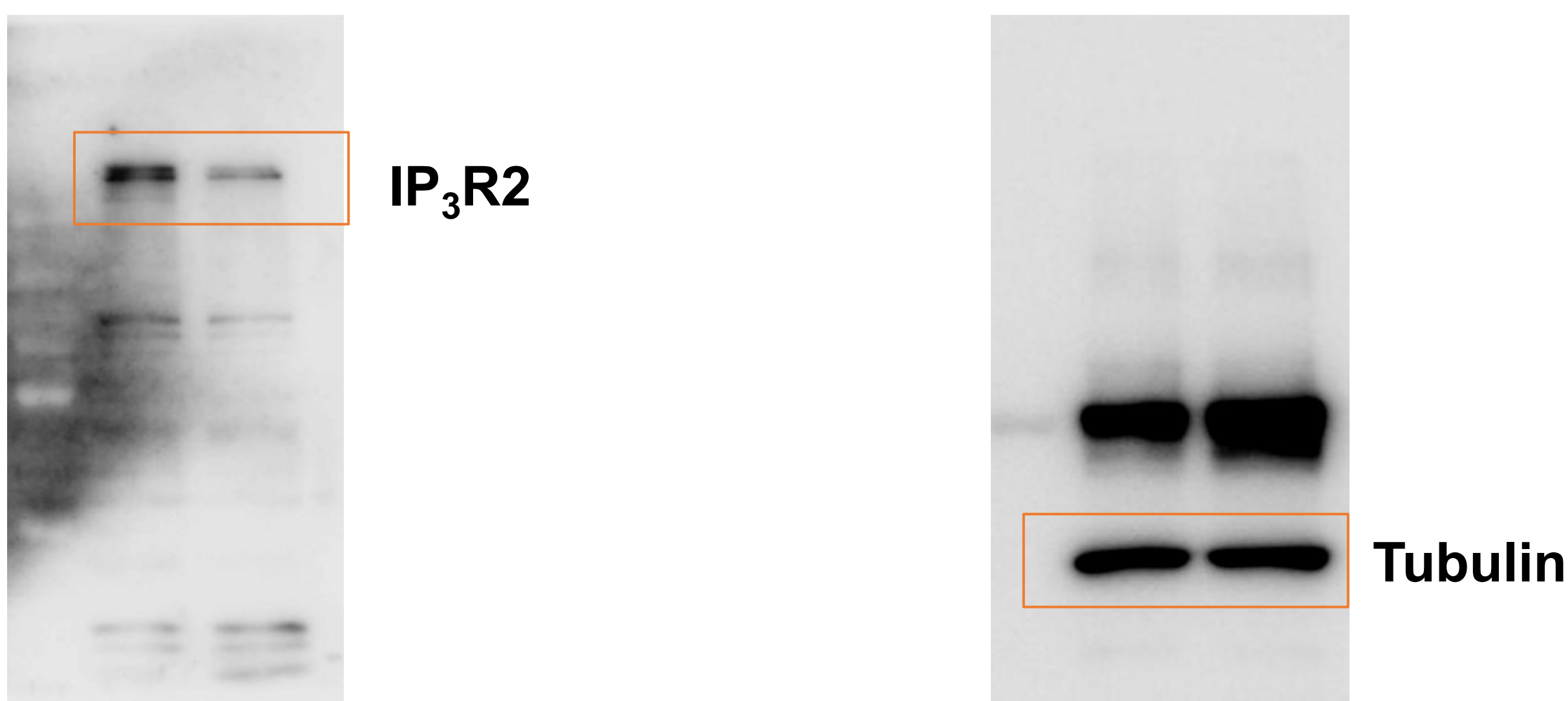

S1K Fig

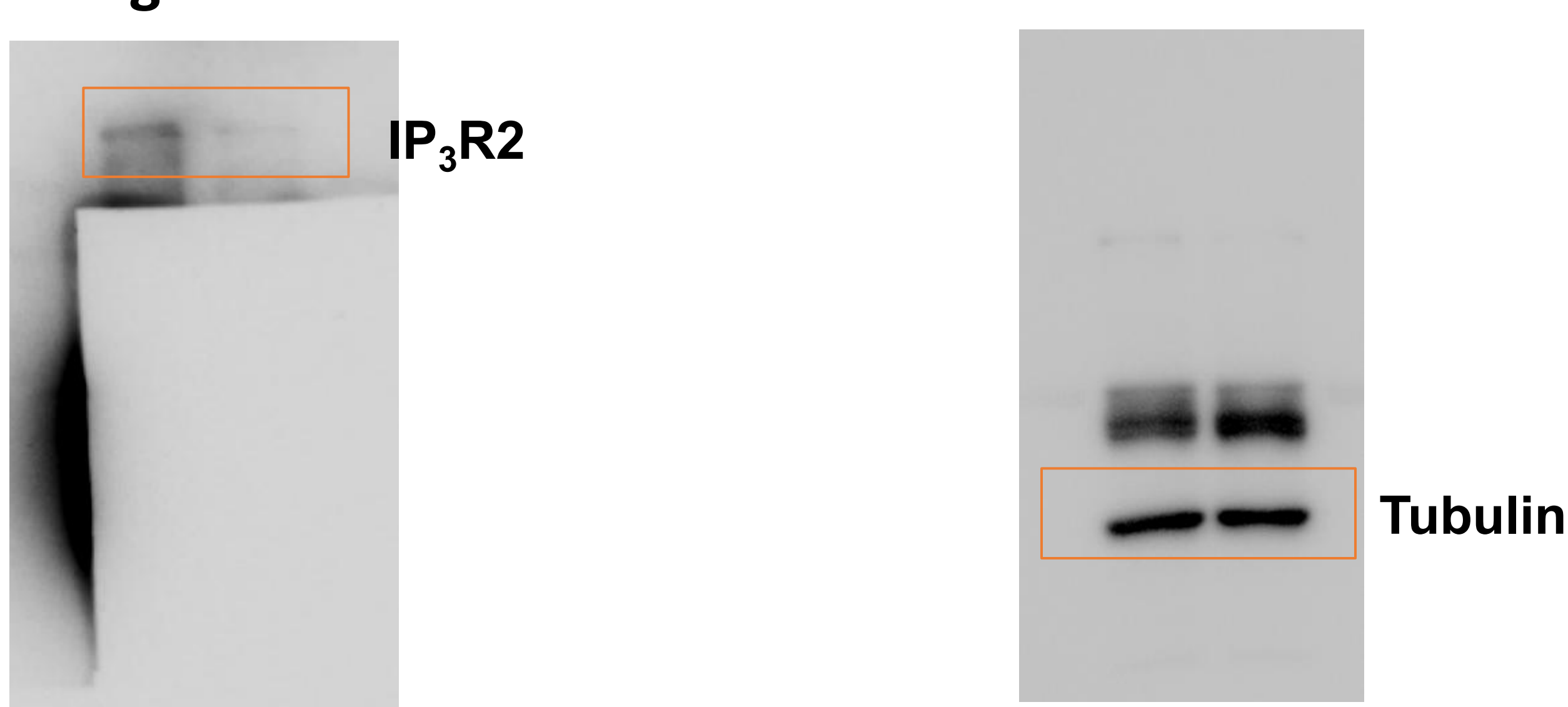

S1M Fig

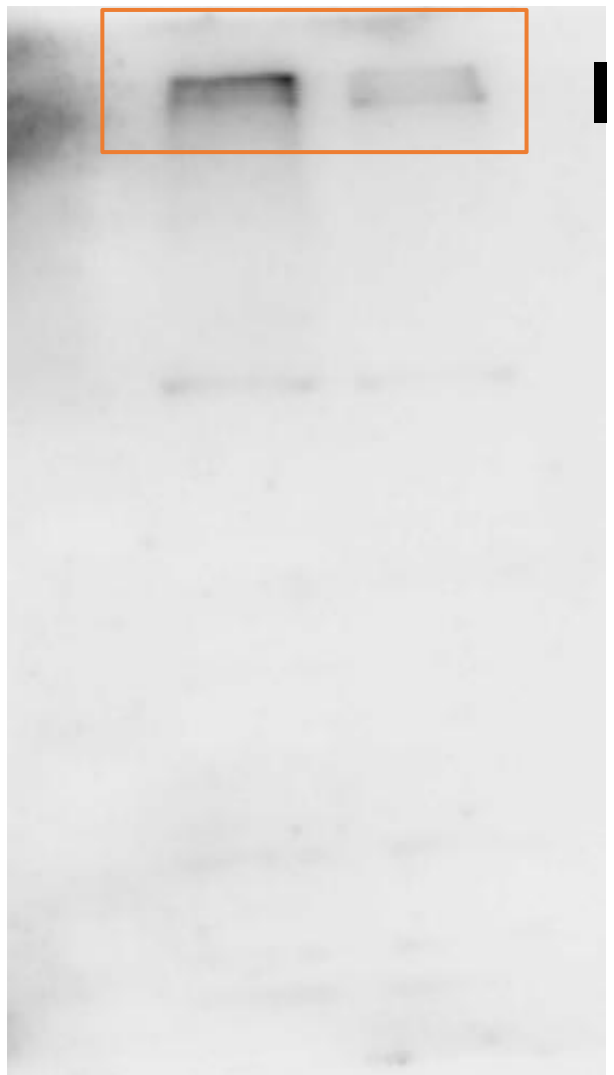

IP$_3$R2

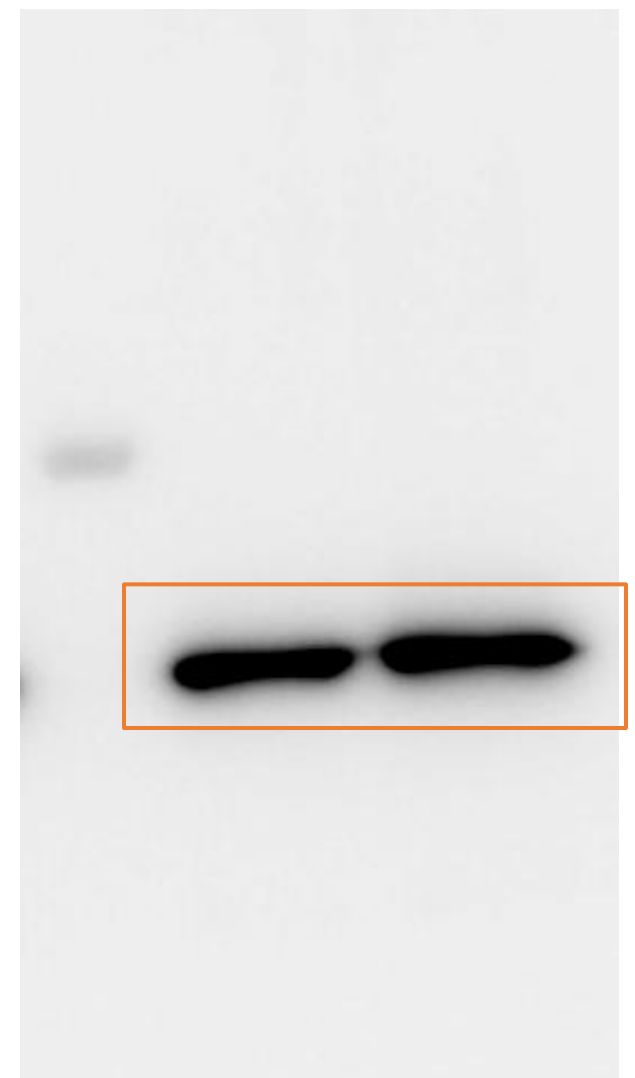

Tubulin

S1Q Fig

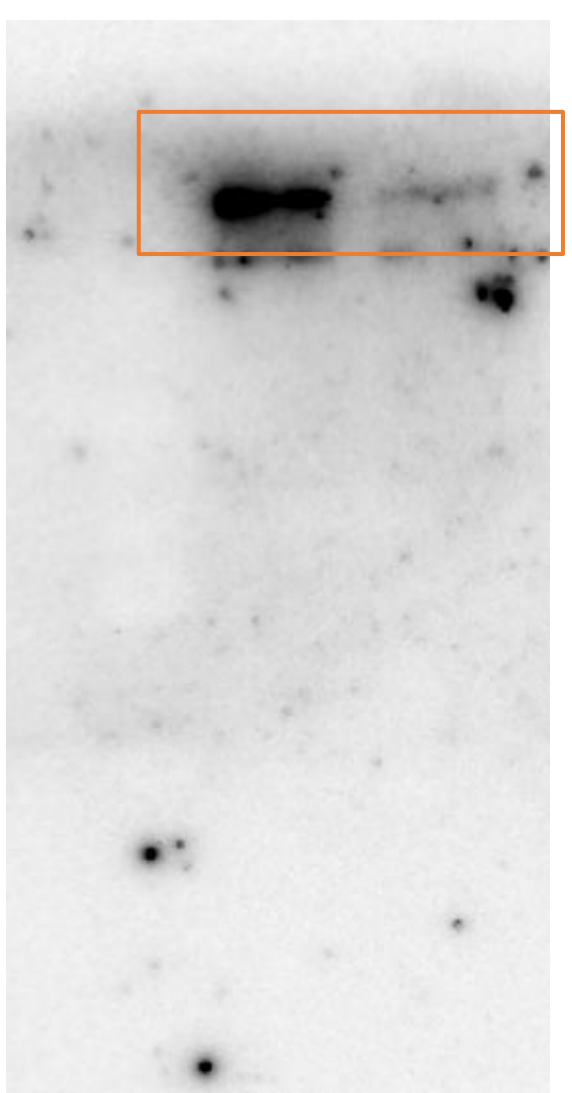

IP$_3$R2

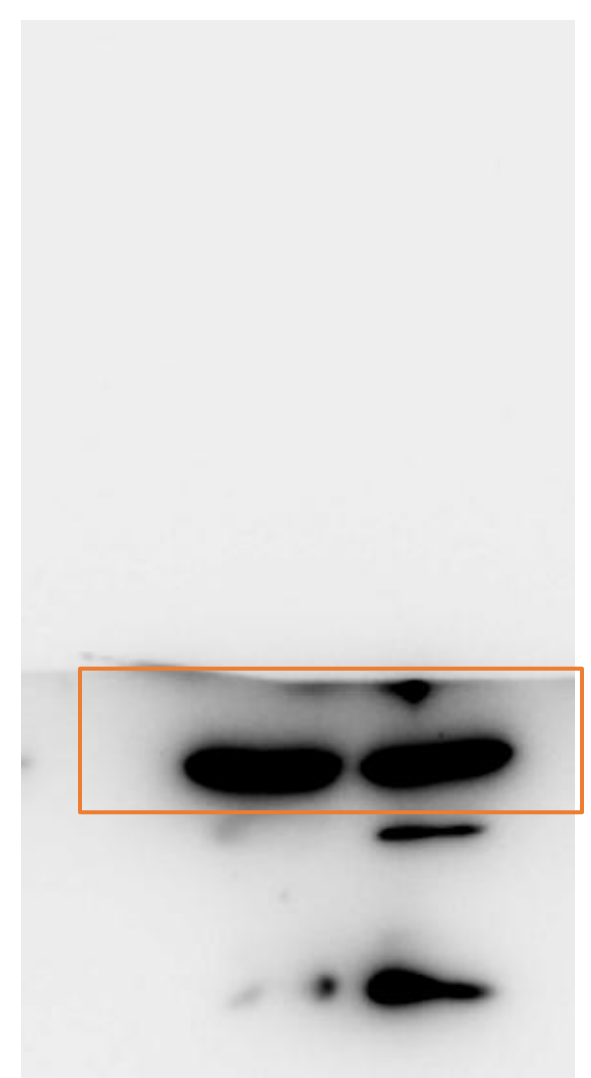

Tubulin

S2E Fig

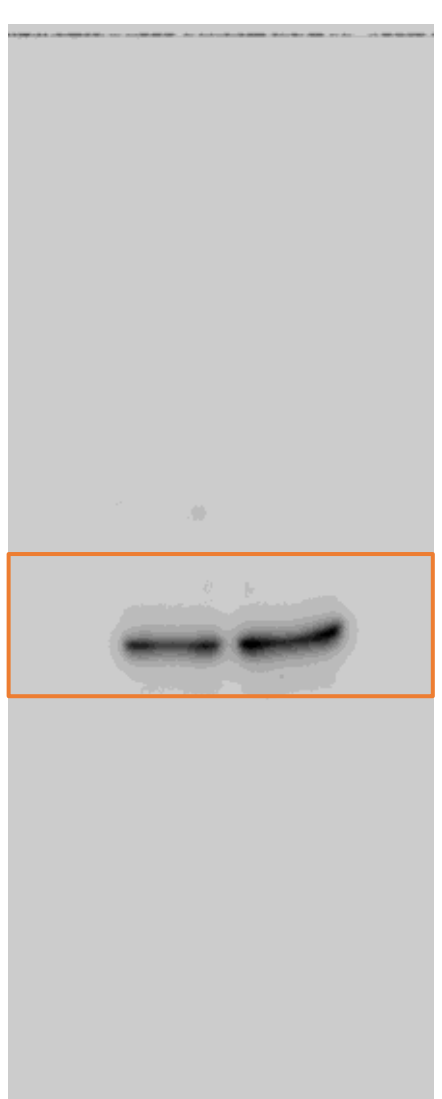

GP100

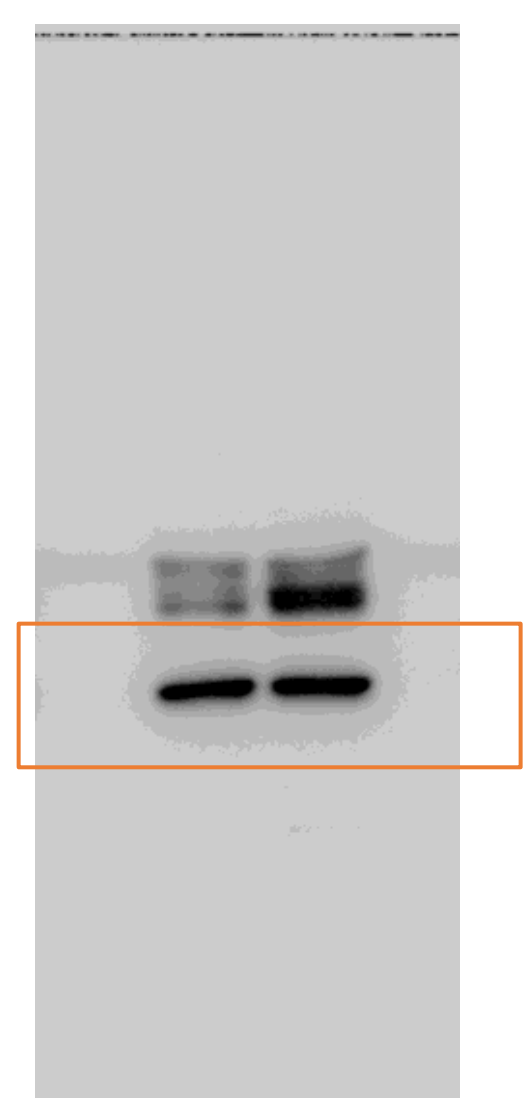

Tubulin

S2G Fig

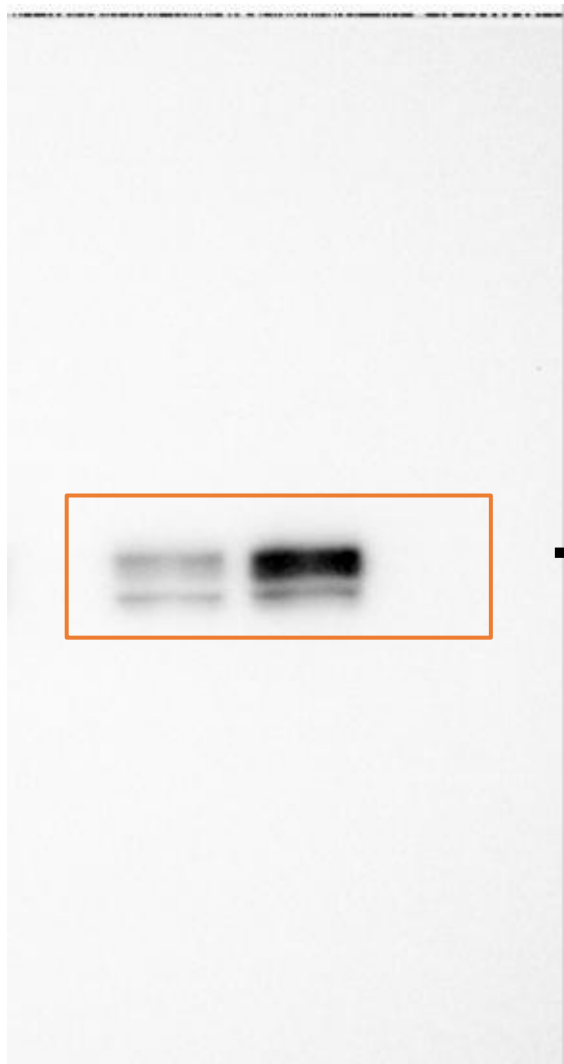

Tyrosinase

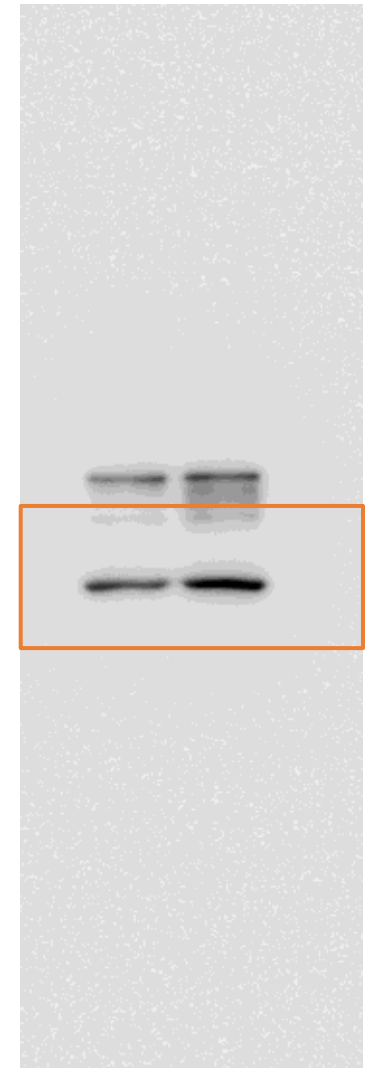

Tubulin

S2I Fig

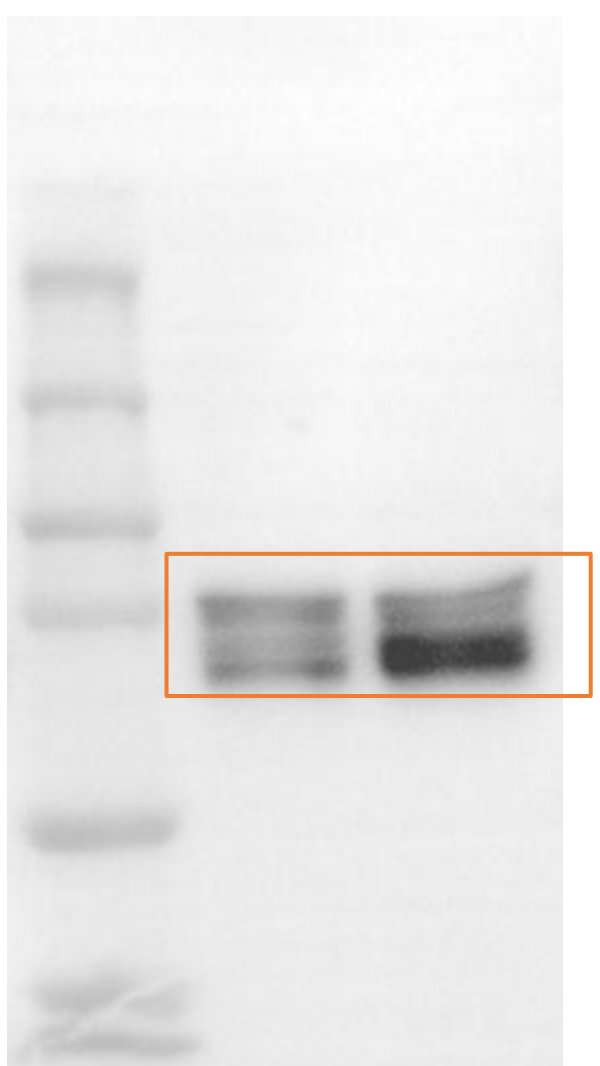

DCT

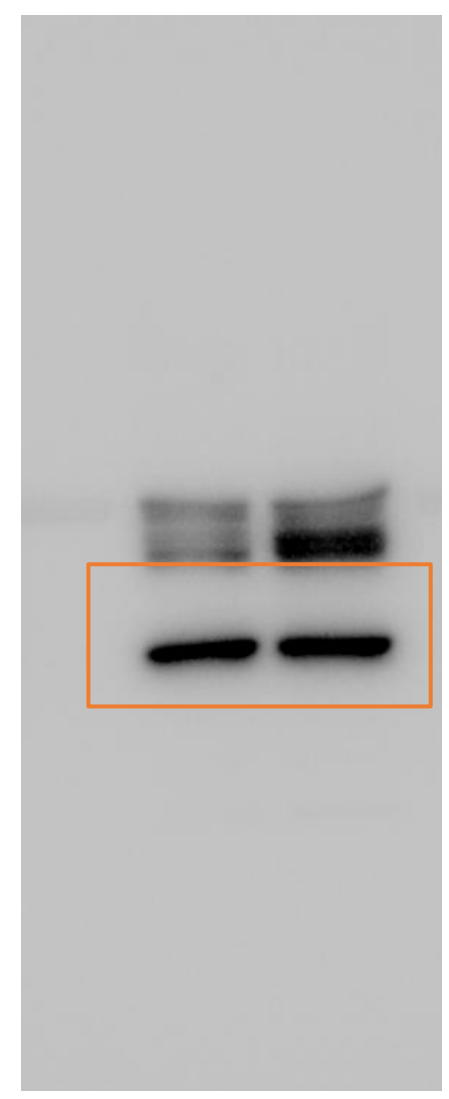

Tubulin

S2K Fig

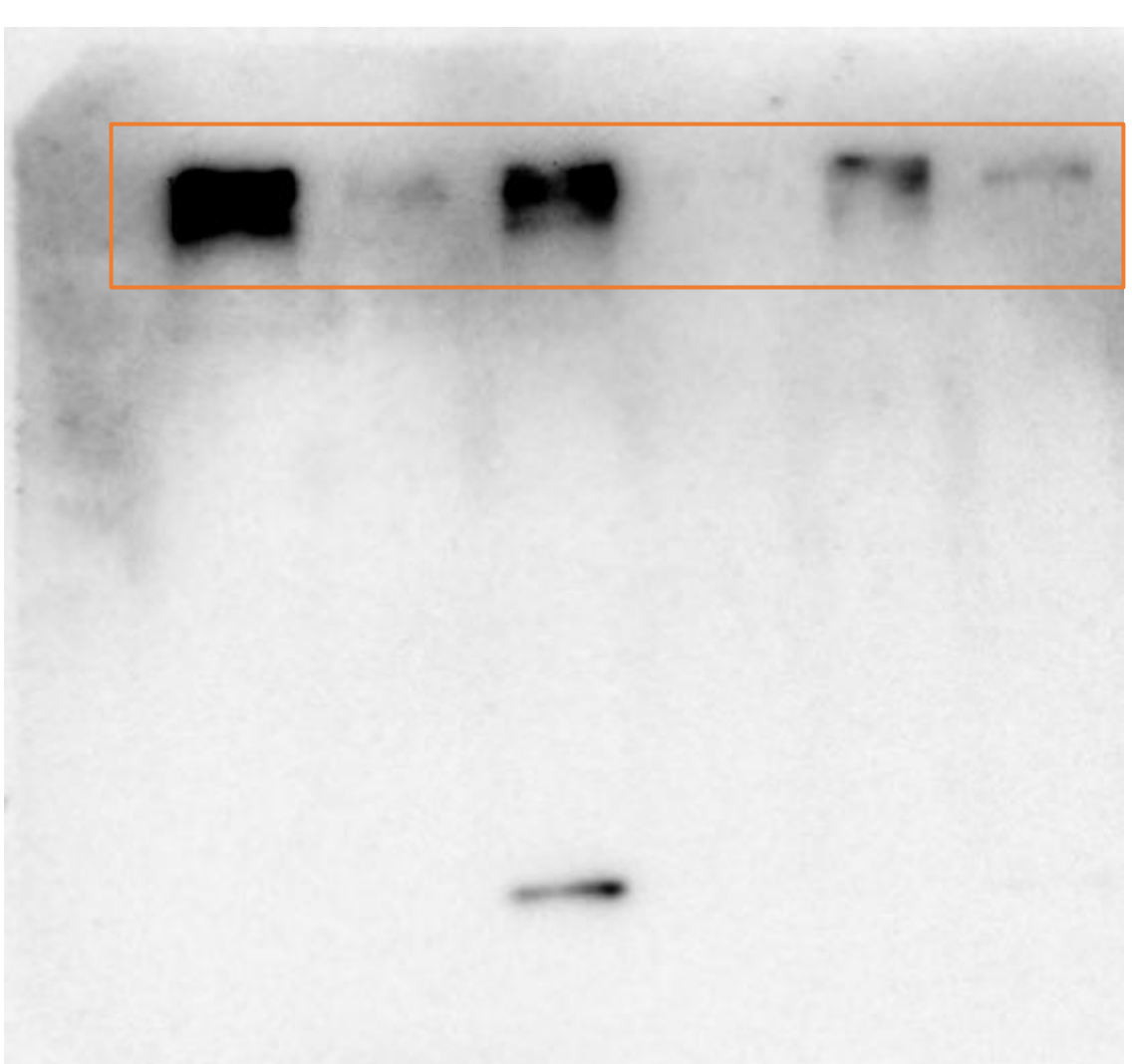

IP$_3$R2

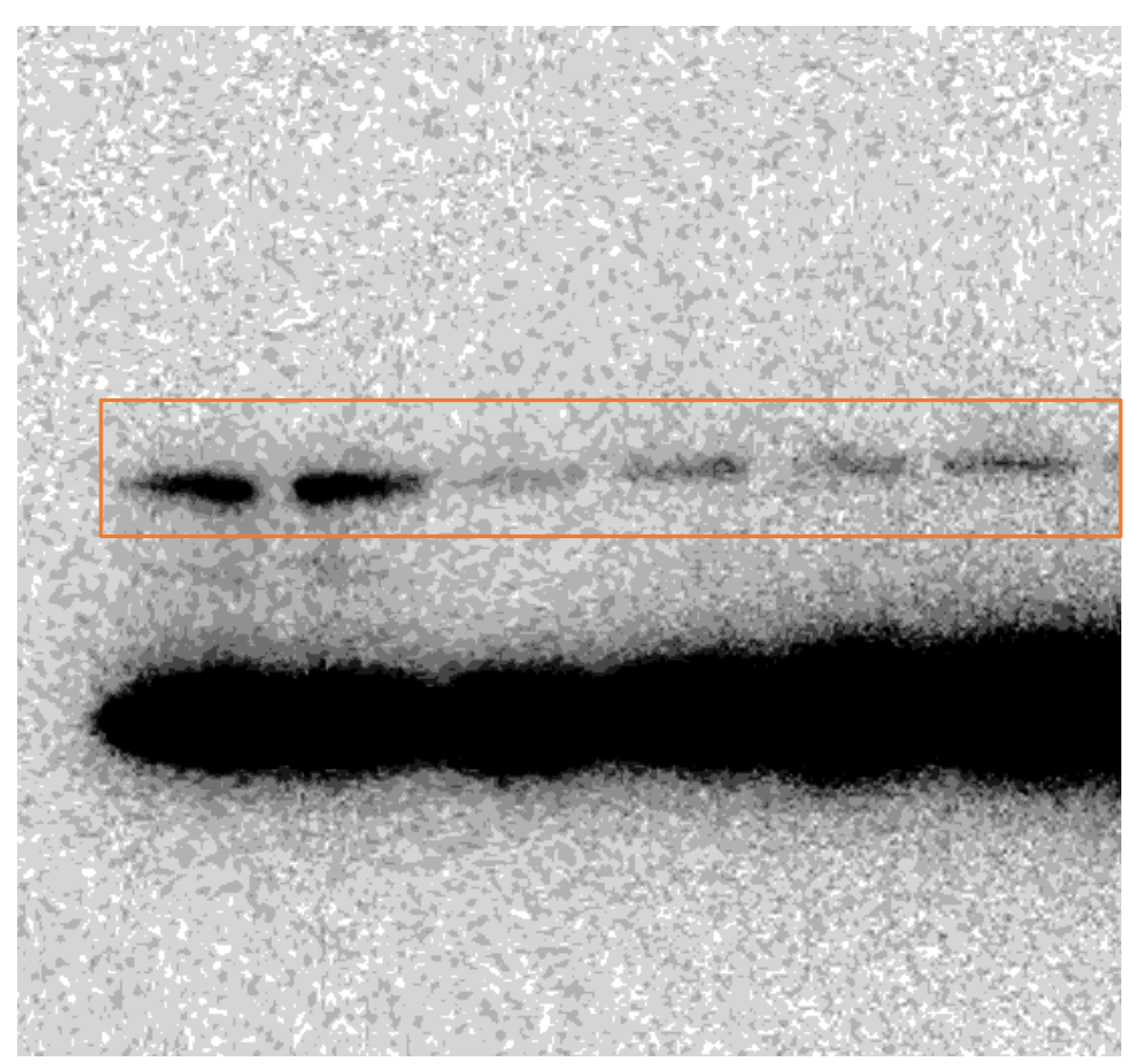

DCT

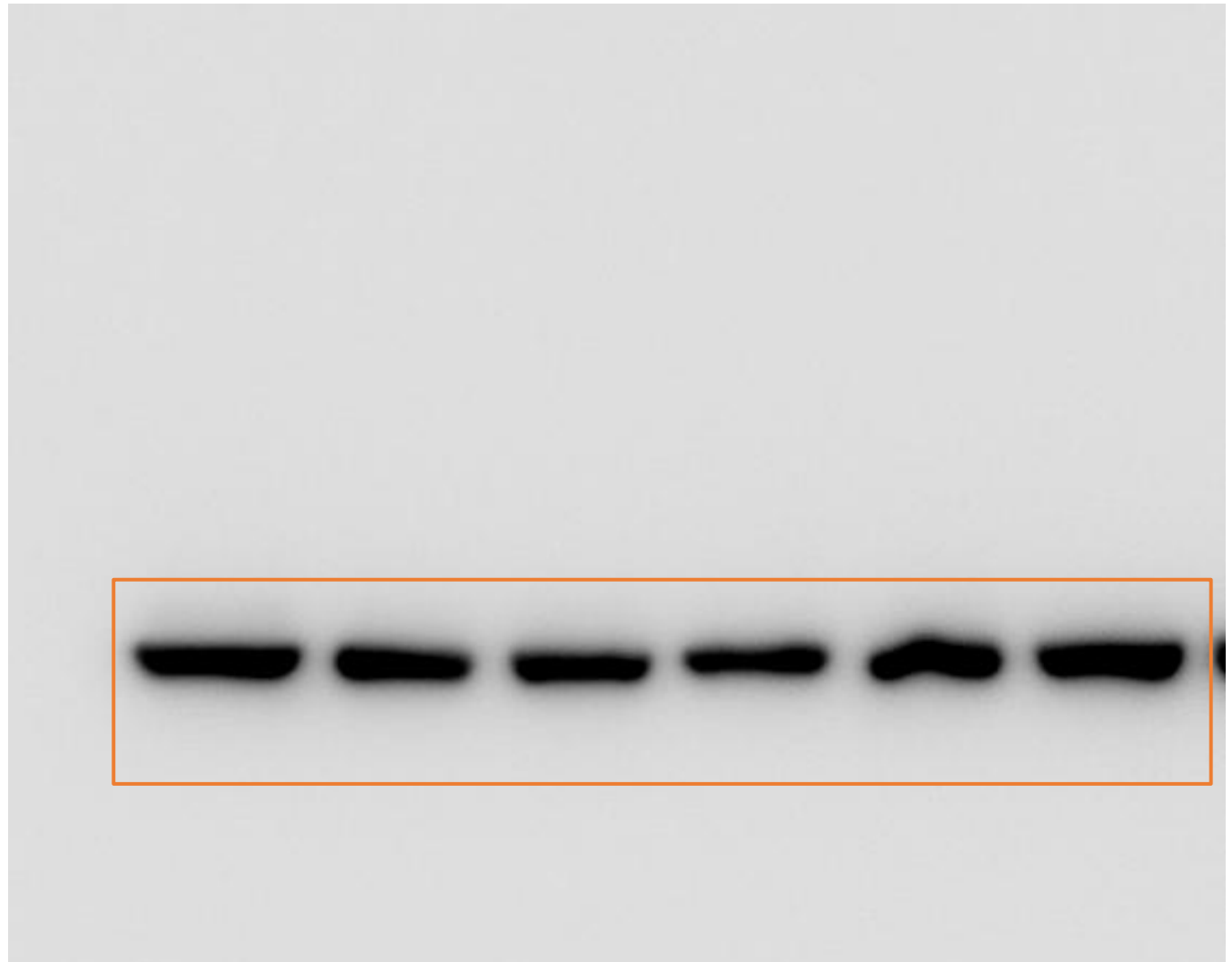

Tubulin

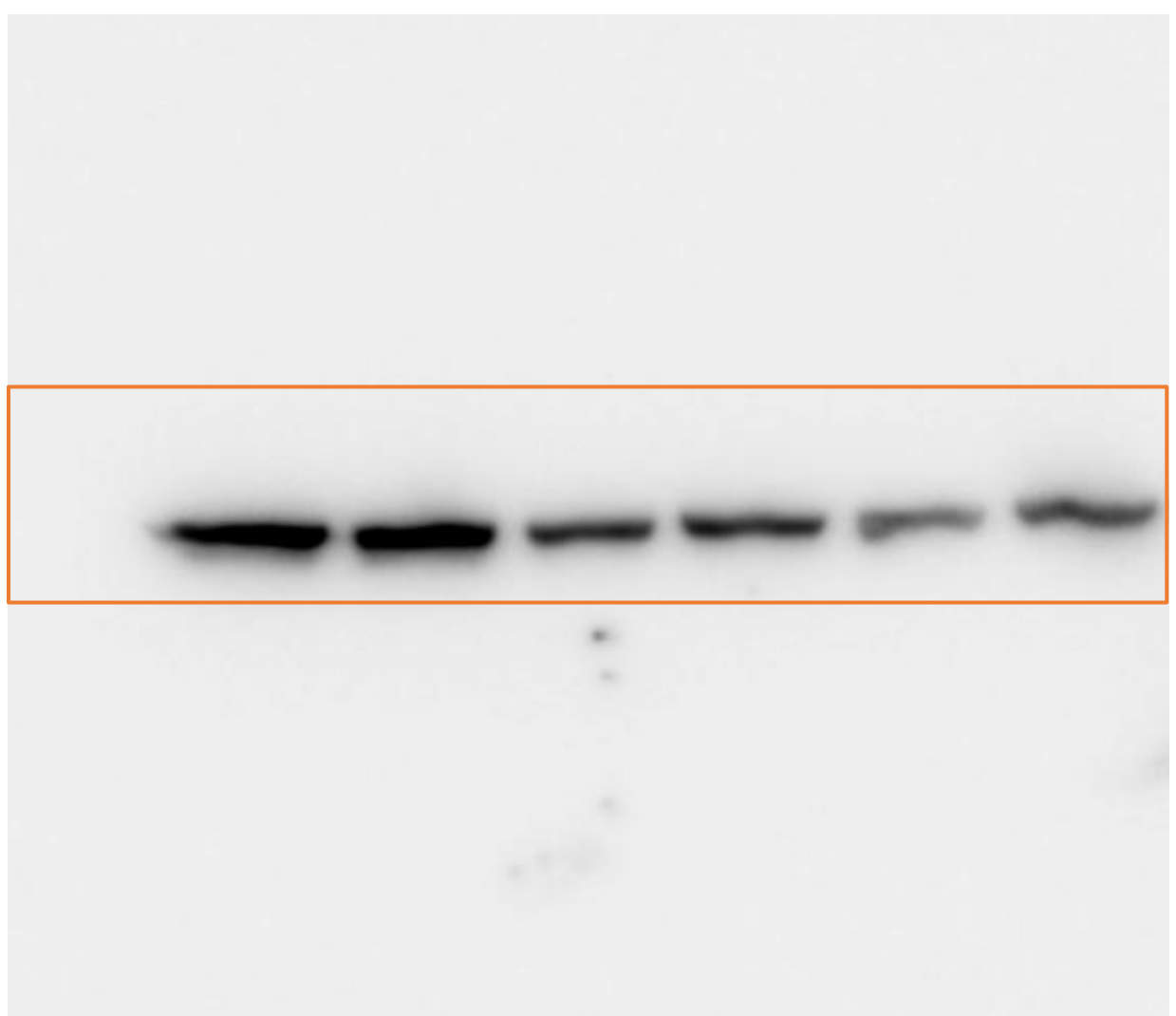

Tyrosinase

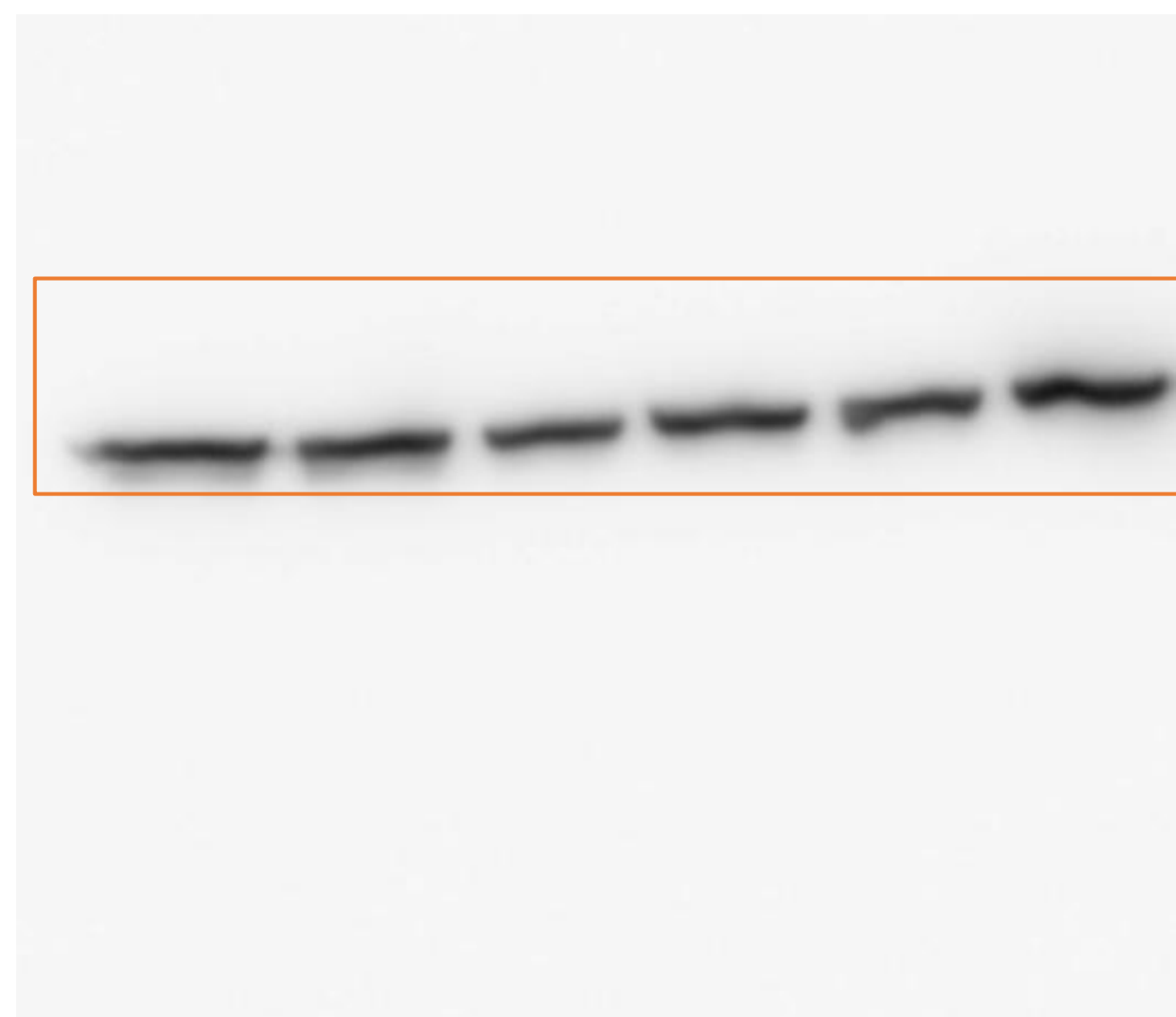

GP100

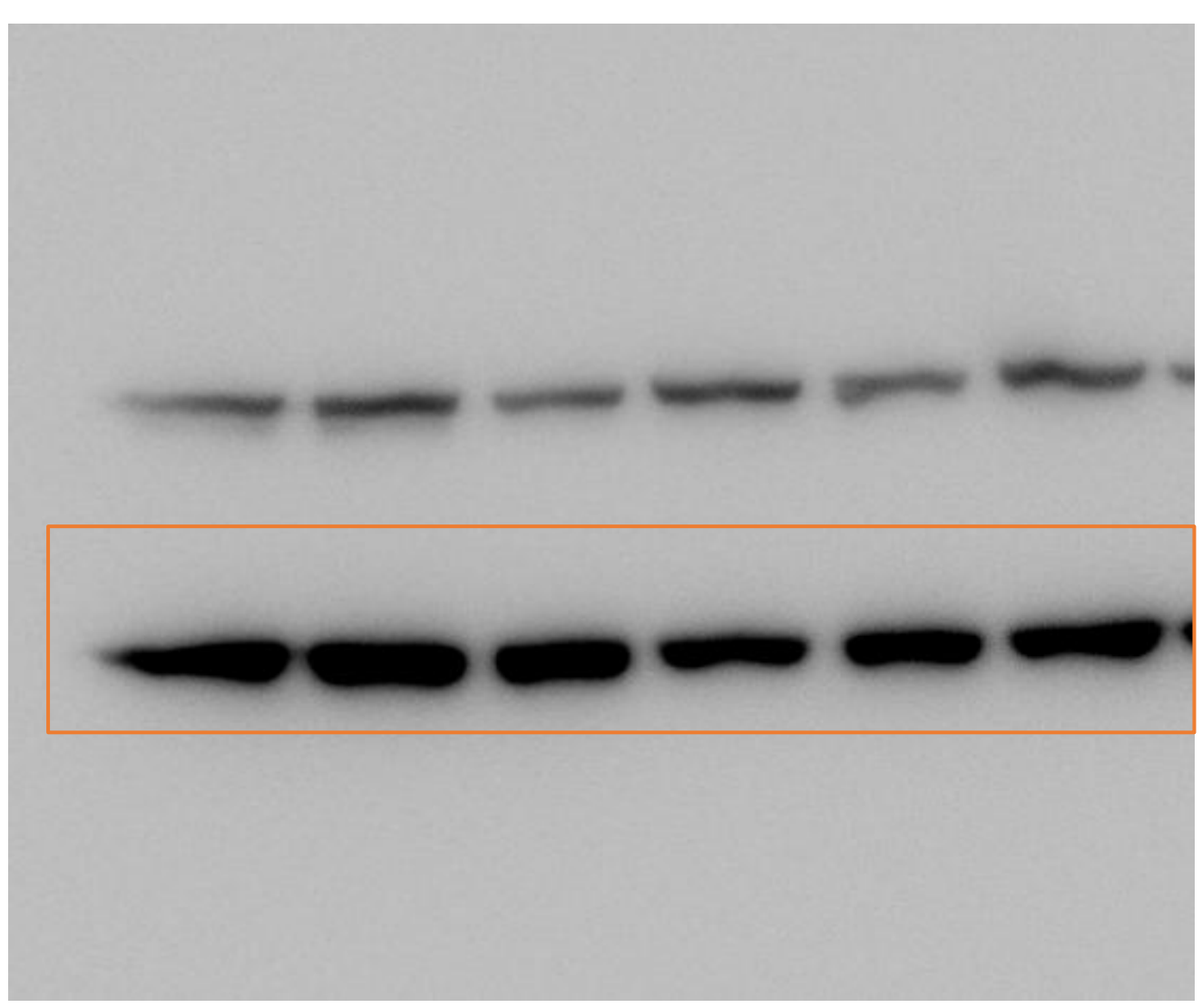

Tubulin

S3A Fig

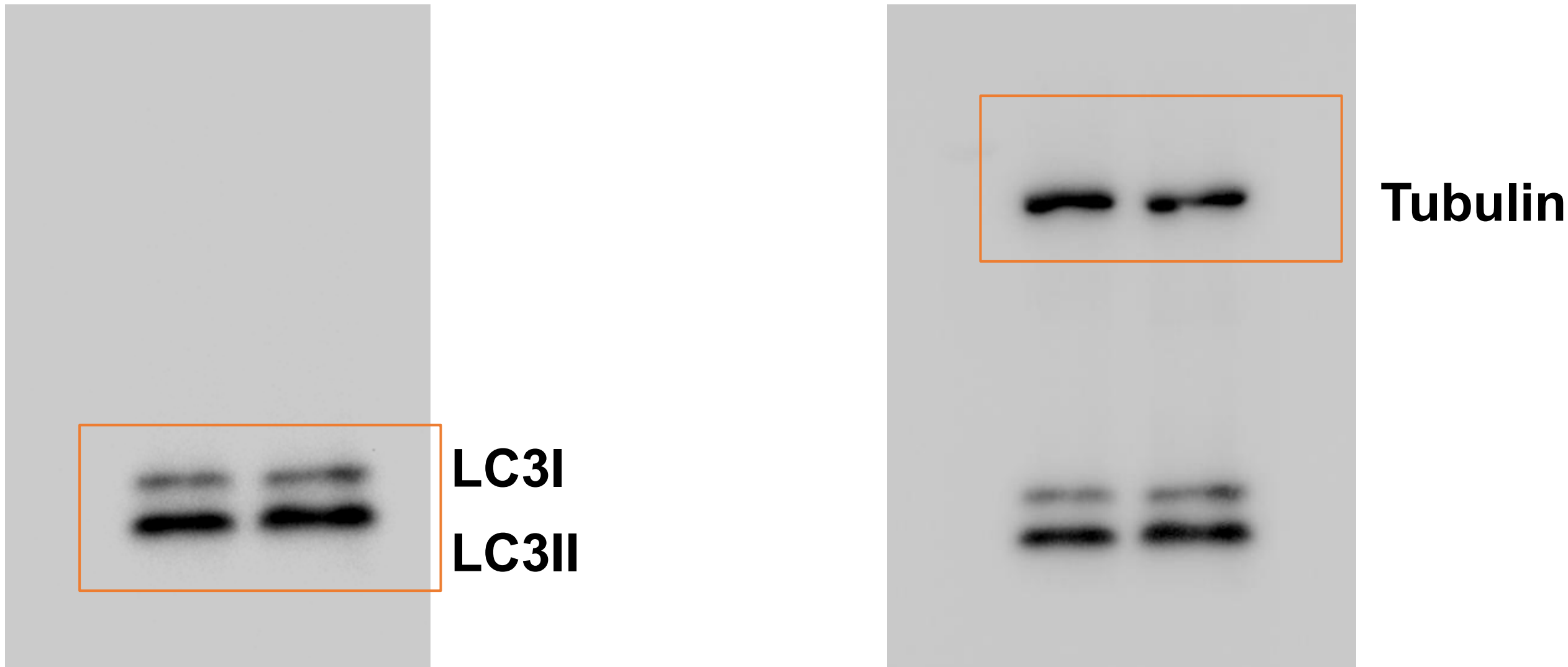

S4C Fig

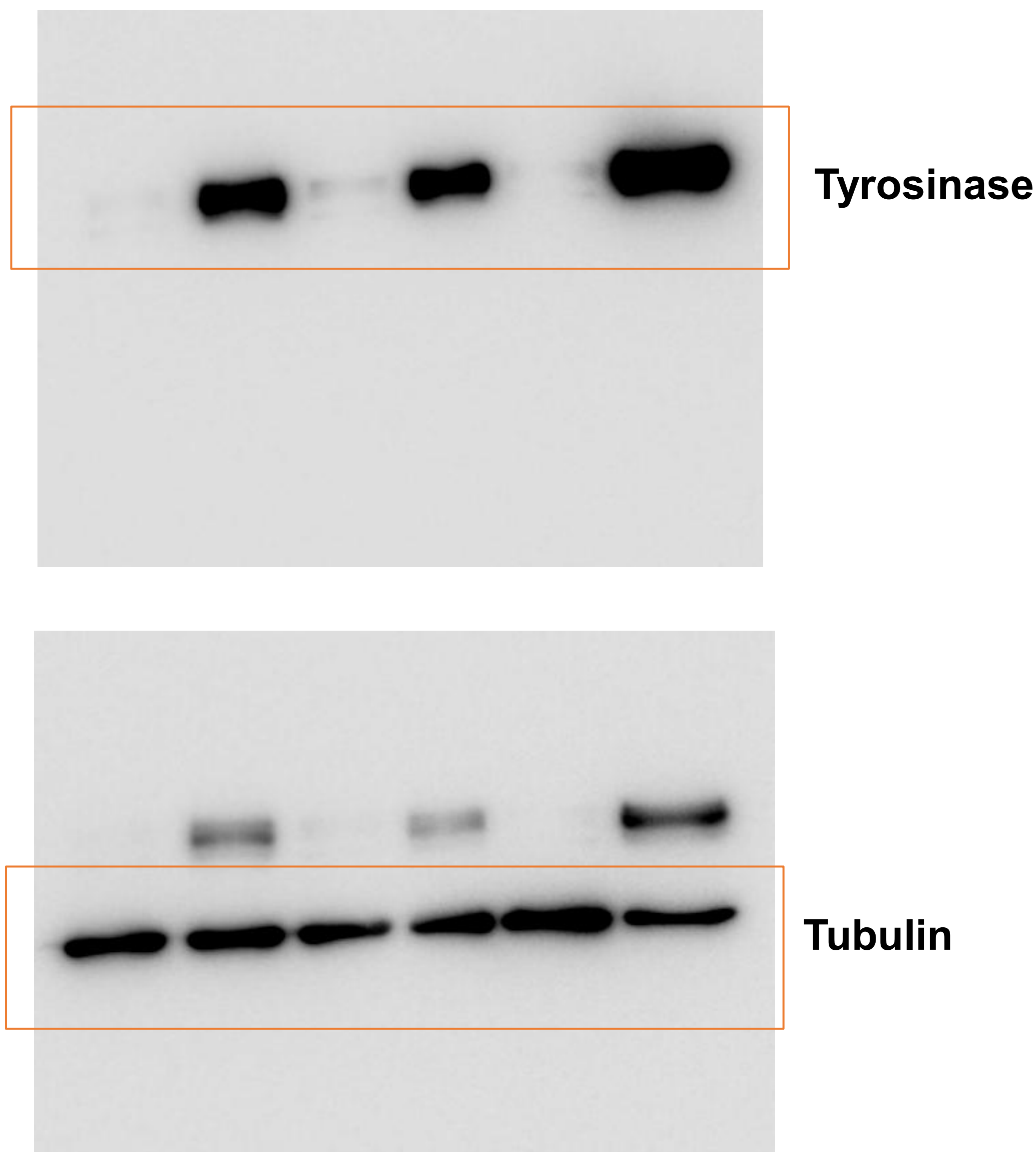

S7A Fig

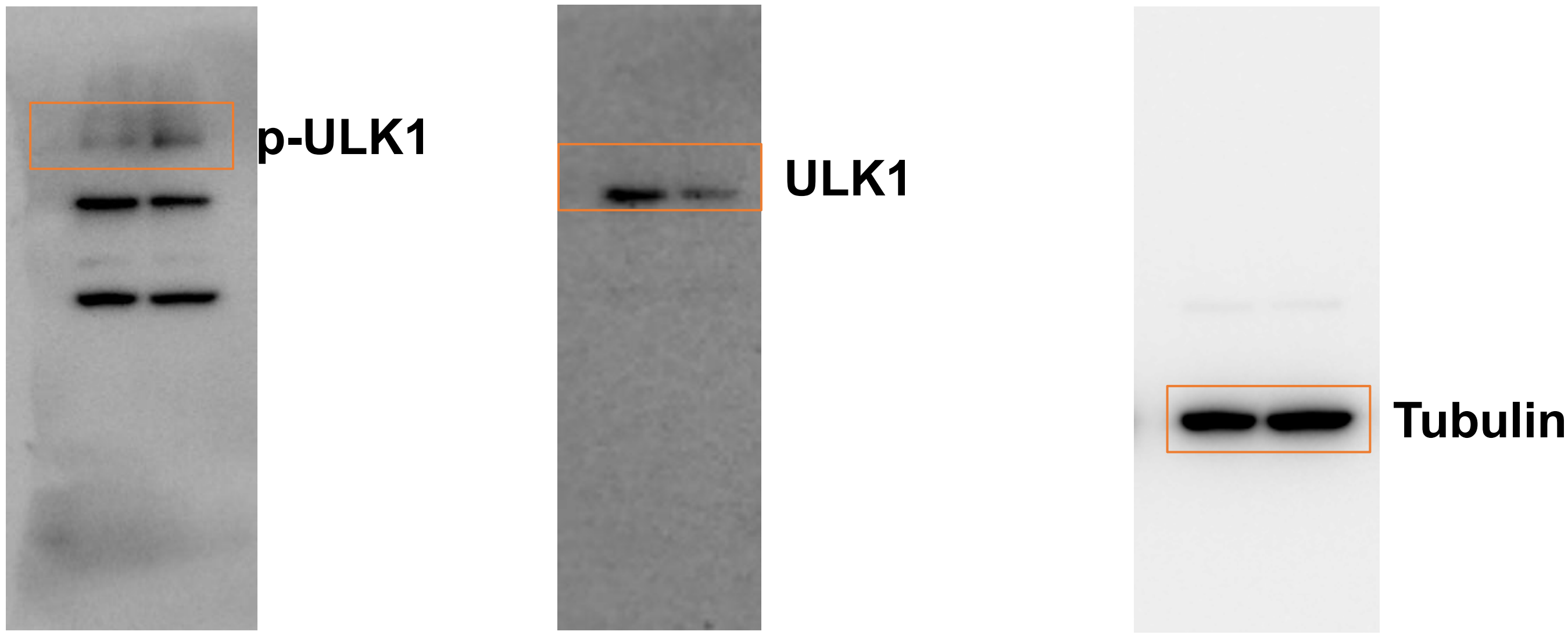

S7C Fig

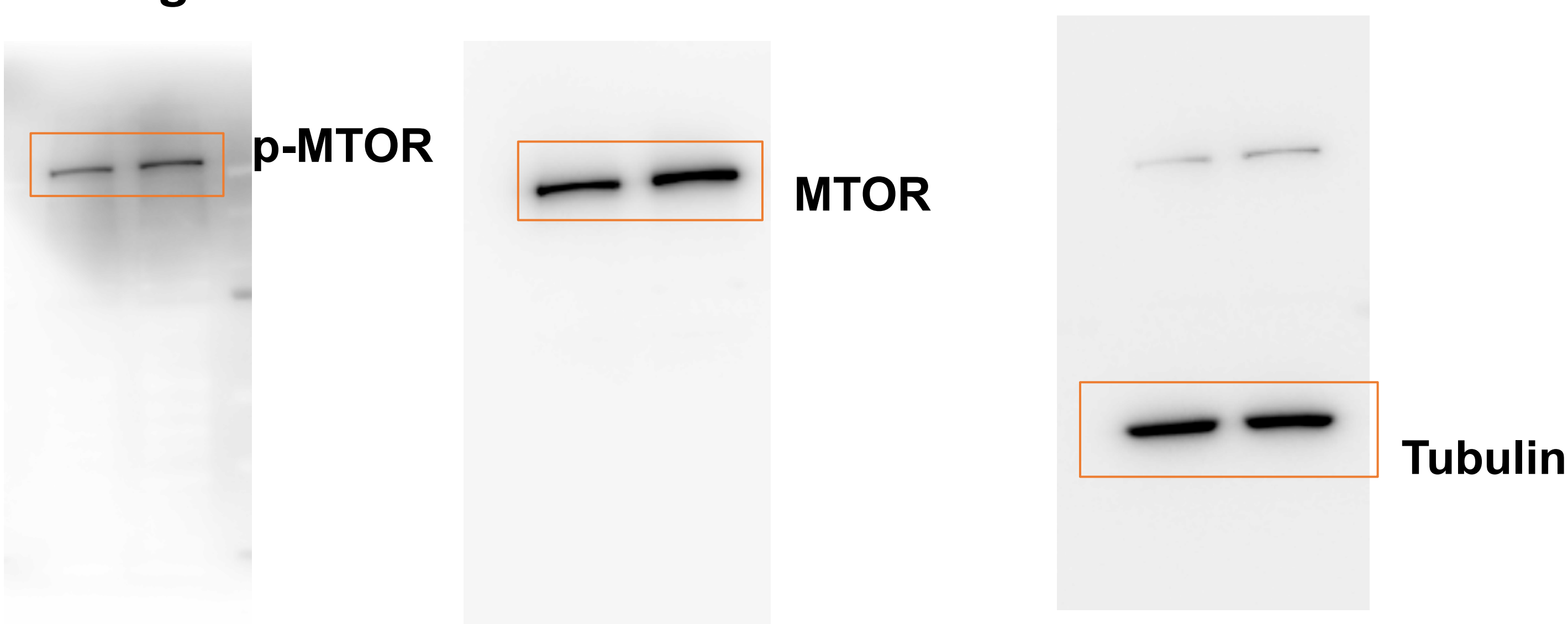

S7E Fig

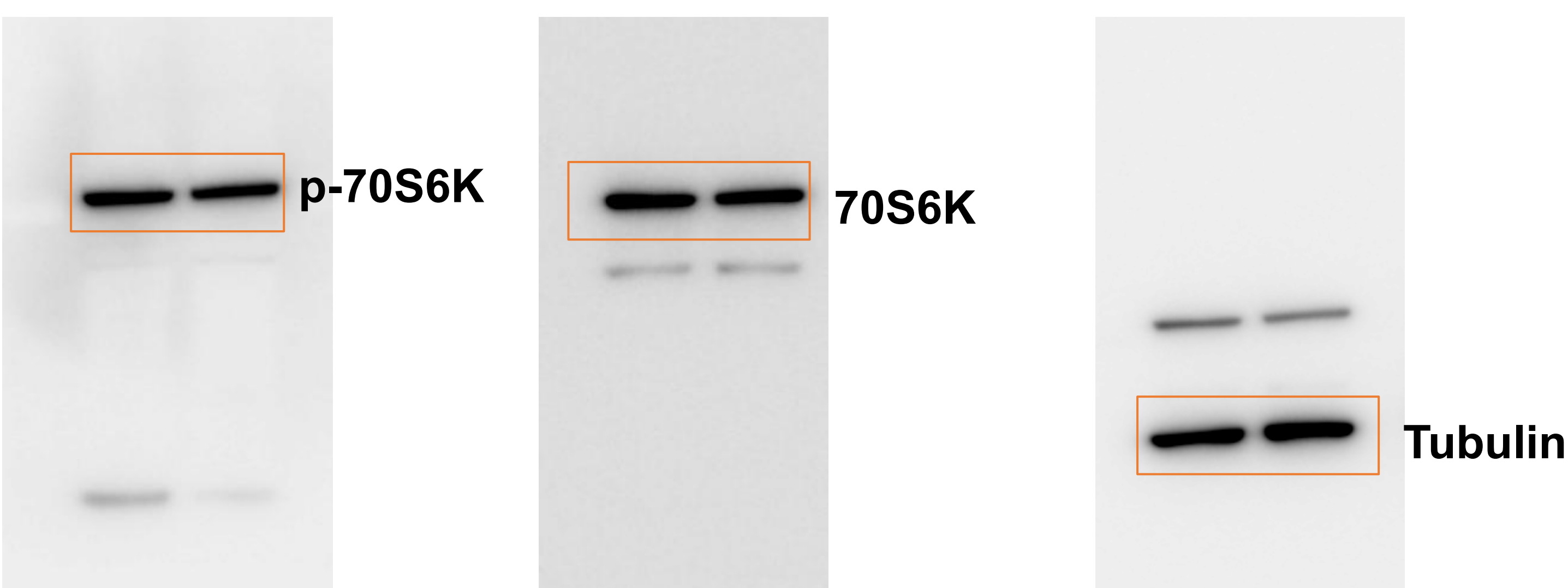

S7G Fig

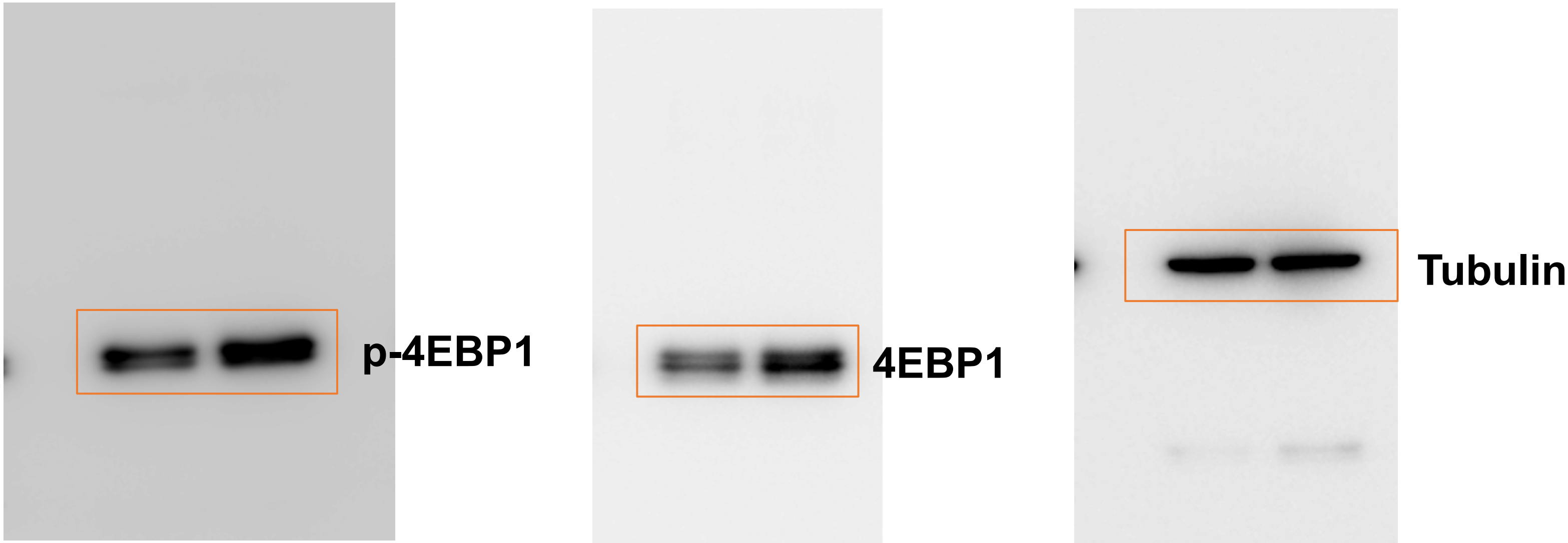

Supplement: Supplementary file 1 [file pbio.3003971.s003.pdf]
