# Supplementary material for: IP3R2-mediated inter-organelle calcium signaling suppresses melanosome degradation
Source: PLoS Biol. 2026 Aug 25;24(8):e3003971. doi: 10.1371/journal.pbio.3003971 (PMC13533435; doi:10.1371/journal.pbio.3003971)
Supplement: Supplementary file 3 [file pbio.3003971.s016.docx]

**Manuscript number:** [RC-2025-03086](https://reviewcommons.msubmit.net/cgi-bin/main.plex?form_type=view_ms&j_id=790&ms_id=159099&ms_rev_no=0&ms_id_key=ftdIq3GEJGalVZVrWUo0BO6kg)

**Corresponding author(s):** Dr. Rajender K Motiani

*[Please use this template only if the submitted manuscript should be considered by the affiliate journal as a full revision in response to the points raised by the reviewers.*

## 1. General Statements [optional]

We thank all three Reviewers for appreciating our work and for sharing constructive feedback to further enhance the quality of our study. It is really gratifying to read that the Reviewers believe that this work is interesting, novel and of interest to broad audience. Therefore, we believe that it will be suitable for a high profile journal. Further, the experiments suggested by the reviewers have added value to the work and have substantiated our findings. It is important to highlight that we have performed all the suggested experiments. Please find below the detailed point by point response to Reviewer’s Comments.

## 2. Point-by-point description of the revisions

**Reviewer #1** (Evidence, reproducibility and clarity (Required):

The manuscript entitled, "IP_3_R2 mediated inter-organelle Ca^2+^ signaling orchestrates melanophagy" is a rather diffuse study of the relationship between IP_3_R2 and melanin production. While this is an interesting and understudied area, the study lacks a clear focus. The model seems to be that IP_3_R2 is essential for mitochondrial calcium loading. And that its absence increases lysosomal calcium loading. There are also a number of incomplete and/or unconvincing links to autophagy/melanophagy, TMEM165, TRPML1 and even gene transcription. In this kind of diffuse study, each step needs to be convincing to get to the next one, which is not the case here. There are also references to altered proteasome function, despite the total absence of any direct data on the proteasome. Finally, I felt it was sometimes unclear whether the authors were referring to melanosomes or lysosomes at various points throughout the study.

While I suspect that, somewhere in here, there are some novel relationships worthy of further investigation, this is a case where the many parts make the overall product less convincing. What effects here are directly relevant to IP_3_R2? This study should stop there, leaving investigations of peripheral factors for future investigations, as the further you get from where you start, the less clear what you are studying becomes. And the less direct.

**Response:** We thank the Reviewer for finding our study interesting and recognizing that this is an understudied area. Further, we appreciate the constructive feedback given by the Reviewer. We have addressed all the Reviewer’s comments. Please find below point-wise responses to the comments.

**Specific Comments:**

**Comment 1.** The separation of Figures 1F and 1J makes it impossible to assess the effect of αMSH on IP_3_R2 expression. This presentation makes interpretation difficult; a simple 4 lane Western would be more informative.

**Response:** We apologize to the Reviewer for not being very clear. Actually, we have separated these data sets because these are two independent experimental conditions. The Figure 1F illustrates data from the LD-based pigmentation model, whereas Supplementary Figure 1K (Previously Fig 1J) depicts data from α-MSH–induced pigmentation model.

**Comment 2.** One of the most attractive points made by this study is that there is a specific link between IP_3_R2 and melanin production. In my opinion, the null hypothesis is that this is just about the amount of IP_3_Rs expressed per cell. To reject this concept, the authors should show data demonstrating the relative expression of all 3 IP_3_Rs. Without this information, the null hypothesis that IP_3_R2 is the most expressed IP_3_R isoform and that's why its knockdown has the most dramatic effect cannot be rejected It would also be helpful to show where the different IP_3_Rs are expressed within the cell.

**Response:** We thank the Reviewer for raising this interesting point and for the constructive comment. As suggested, we would like to clarify that the relative expression of all three IP₃R isoforms has already been analyzed in our study. Specifically, in **Figure 1B**, we demonstrate the expression pattern of IP₃R isoforms in our experimental system, where IP₃R2 shows the highest expression level, followed by IP₃R3 and IP₃R1 (IP₃R2 > IP₃R3 > IP₃R1). Further, in the revised manuscript, we additionally analyzed publicly available datasets for IP₃Rs expression. “The Human Protein Atlas” reports a higher expression of IP₃R2 in melanocytes compared to the other IP₃R isoforms (**Supplementary Fig 1A**). Therefore, we agree with the Reviewer’s proposed concept that the relatively higher expression of IP₃R2 can be one of the important factors that regulate pigmentation levels. Indeed, our analysis of microarray dataset from African vs Caucasian skin revealed a greater IP₃R2 expression in African skin compared to Caucasian skin (**Figure 1L).**

With respect to subcellular localization, all three IP₃R isoforms are predominantly localized to the endoplasmic reticulum, consistent with their established role as ER-resident Ca²⁺ release channels. However, their expression levels are known to be highly cell and tissue specific (Bartok *et al*., Nature Communications 2019), supporting the idea that higher IP₃R2 levels play a functionally specialized role in melanogenesis.

**Comment 3.** It would be helpful to label Figs 3F-I with the conditions used. The description in the text is of increased LC3II levels, however, the ratio of LC3I to LC3II might be more meaningful. Irrespective, although the graph shows an increase in LC3II, the Western really doesn't show much. As a standalone finding, I don't find this figure to be very convincing; there are better options to demonstrate this proposed relationship between IP_3_R2 and autophagy than what is shown.

**Response:** We sincerely thank the Reviewer for this thoughtful and critical evaluation, which has helped us improve the clarity and precision of this analysis. To address this concern, in the revised manuscript, we have now labeled ‘**LD**’ in the **Supplementary Fig 2A-B** (Previously, Fig 4F-I) with the corresponding experimental conditions for clarity. In addition, we reanalyzed the data by calculating the LC3II/LC3I ratio in all the figures of the revised manuscript that include LC3II expression, which provides a more meaningful and robust assessment of autophagic flux. This revised analysis yields a clearer representation of LC3 dynamics and strengthens the interpretation of the western blotting data in support of the relationship between IP₃R2 and autophagy. Further, we have shown by confocal imaging that IP_3_R2 silencing significantly reduced GFP/RFP ratio of the pMRX-IP-GFP-LC3-RFP reporter system in comparison to control condition in **Fig 4M-N** to demonstrate the relationship between IP_3_R2 and autophagy. Collectively, these autophagy flux assays and biochemical experiments clearly demonstrate a direct relationship between IP_3_R2 and autophagy.

**Comment 4.** The following statement at the beginning of page 22 "We observed an impaired proteasomal degradation of critical melanogenic proteins localized on melanosomes in the IP_3_R2 knockdown condition" is insufficiently supported by data to be made. Even if I was convinced that autophagy was enhanced, there is no data of any kind about the proteasome in this manuscript.

**Response:** We appreciate the Reviewer’s careful scrutiny of this statement and the opportunity to clarify and strengthen our interpretation. To directly address the concern regarding proteasomal involvement, in the revised manuscript, we performed additional experiments using MG132, a well-established inhibitor of proteasomal degradation. These experiments were designed to assess whether the altered stability of melanogenic proteins observed upon IP₃R2 knockdown could be attributed to changes in proteasome-mediated turnover.

In the revised manuscript, our new data show that treatment with MG132 leads to a marked reduction in the levels of melanosome-associated melanogenic proteins, including GP100 and DCT, compared to the DMSO control (**Fig. 4A–D**). This response contrasts with that of non-melanosomal proteins, such as IP₃R2 and Calnexin, which are localized to the endoplasmic reticulum and exhibits increased accumulation upon MG132 treatment (**Fig. 4E–H**), consistent with canonical proteasomal inhibition. These differential outcomes suggest that melanosome-resident proteins respond distinctly to proteasomal blockade, likely due to their compartmentalized localization on melanosomes.

Previous studies have shown that impairment of proteasomal function can activate autophagy as a compensatory, cytoprotective mechanism (Williams et al, 2013; Li et al, 2019; Su & Wang, 2020; Pan et al, 2020). Indeed, we observed a significant increase in LC3II/LC3I levels in IP_3_R2 knockdown plus MG132 treatment condition in comparison to IP_3_R2 knockdown plus the DMSO control (**Fig. 4I–J**).

To investigate whether impairment of proteasomal degradation upon IP_3_R2 silencing alone or together with MG132 selectively triggers melanophagy, we assessed melanophagy using melanophagy reporter, mCherry-Tyrosinase-eGFP following IP_3_R2 silencing along with MG132 treatment. Our observations revealed an increase in melanophagy flux with IP_3_R2 silencing and MG132 treatment compared to siNT with DMSO control (**Fig 5K-L**). This suggests that IP_3_R2 silencing induced inhibition of proteasomal degradation activates melanophagy. Taken together, these findings indicate that compromised proteasomal degradation engages the autophagy machinery, providing a mechanistic link between proteasome dysfunction, enhanced autophagy, and altered melanogenic protein turnover.

**Comment 5.** In figure 5, the authors create a new ratiometric dye to detect melanosome stability based on the principle that tyrosinase is exclusively found in melanosomes. Unfortunately, there is no validation that this new construct is found exclusively in melanosomes upon expression. In addition, there is discussion about the pH of lysosomes, but not of melanosomes. Ultimately, this data cannot be considered at face value without any type of validation; I also note that the pictures lack sufficient detail to support identification of these structures as melanosomes.
While I maintain the above concerns, I note that, the data in supplemental figure 3 is MUCH more convincing than what is in the figure. Both the writing and the figure design should be rethought.

**Response:** We appreciate the Reviewer’s thorough evaluation and constructive critique of **Figure 5**, which has helped us to better clarify and validate this aspect of the study. In the revised manuscript, we directly address the concern regarding the subcellular specificity of the ratiometric probes, we performed detailed colocalization analysis using established melanosome markers. Specifically, we assessed the localization of the melanophagy detection probes mCherry–Tyr–eGFP and tyrosinase–mKeimaN1 with the melanosome-resident protein GP100 detected by anti-HMB45 (**Supplementary Fig 2E-F and 2K-L**). These analyses revealed a very high degree of colocalization, reflected by strong Pearson’s correlation and overlap coefficients, thereby validating that the expressed probes are predominantly localized to melanosomes.

Regarding Lysosome/Melanosomal pH considerations, our melanophagy detection ratiometric probes: mCherry–Tyrosinase–eGFP (sensitive to acidic pH via eGFP) and tyrosinase mKeimaN1 (sensitive to acidic pH via Keima) are specifically designed to identify melanosome degradation, which happens upon melanosome fusion with lysosome. Consequently, the observed signal shifts indicate melanosome turnover rather than merely reflecting the lysosomal pH.

To further corroborate the microscopic observations, we performed biochemical assays to study melanophagy flux upon IP_3_R2 silencing. We employed Bafilomycin A1, an inhibitor of autophagosome-lysosome fusion, to examine melanosomal protein accumulation. Upon Bafilomycin A1 treatment, IP_3_R2 silenced cells showed enhanced accumulation of melanosomes, as indicated by elevated tyrosinase levels compared with siNT controls (**Supplementary Fig 3C-D**), indicating elevated melanophagy flux upon IP_3_R2 knockdown. In the revised manuscript, we employed additional melanophagy detection strategies to further strengthen our findings. Specifically, we used Retagliptin phosphate (RTG), a well-established selective inducer of melanophagy, and observed a marked increase in melanophagy using the mCherry–Tyrosinase–eGFP melanophagy probe (**Supplementary Fig 2G-H**). Additionally, we performed independent validation by assessing colocalization of the melanosome (recognized by anti-HMB45 ab that identifies melanosomal structural protein GP100) with LC3 (**Supplementary Fig 3A-B**). This analysis revealed a significant increase in melanosomes colocalization with LC3 upon IP₃R2 silencing compared to control conditions.

Collectively, these independent approaches clearly demonstrate that the melanophagy probes localize to melanosomes and detect melanophagy (by responding to melanosome fusion to lysosomes).

**Comment 6.** Given the increase in ER Ca^2+^ content after IP_3_R2 knockdown, ER calcium content should be emptied before attempting to estimate lysosomal Ca^2+^ content with GPN or Bafilomycin. Otherwise, the source of calcium is less than clear.

**Response:** We appreciate the Reviewer’s careful consideration of Ca²⁺ source, which is critical for accurate interpretation of these experiments. Therefore, as suggested, in the revised manuscript, we conducted experiments involving Thapsigargin (Tg) pre-treatment to deplete ER Ca²⁺ reserves before examining lysosomal Ca²⁺ release using GPN or Bafilomycin (**Supplementary Fig 6I-N**). Even under these conditions, we noted increased lysosomal Ca²⁺ release in IP₃R2 knockdown cells, thus confirming that the observed Ca²⁺ signals originate from lysosomes rather than any remaining ER Ca²⁺. Importantly, this approach allowed us to minimize ER-derived Ca²⁺ contributions to changes in the lysosomal Ca²⁺ release.

**Reviewer #1 (Significance (Required)):**

The manuscript entitled, "IP_3_R2 mediated inter-organelle Ca^2+^ signaling orchestrates melanophagy" is a rather diffuse study of the relationship between IP_3_R2 and melanin production. While this is an interesting and understudied area, the study lacks a clear focus. The model seems to be that IP_3_R2 is essential for mitochondrial calcium loading. And that its absence increases lysosomal calcium loading. There are also a number of incomplete and/or unconvincing links to autophagy/melanophagy, TMEM165, TRPML1 and even gene transcription. In this kind of diffuse study, each step needs to be convincing to get to the next one, which is not the case here. There are also references to altered proteasome function, despite the total absence of any direct data on the proteasome. Finally, I felt it was sometimes unclear whether the authors were referring to melanosomes or lysosomes at various points throughout the study.

**Response:** We thank the Reviewer for finding our work interesting and appreciating that this is an understudied field. Further, we thank him/her for the constructive feedback on our study. We have performed several additional experiments and significantly revised the manuscript to address all the comments of the Reviewer.

**Reviewer #2** (Evidence, reproducibility and clarity (Required)):

In the present manuscript, Saurav *et al*. identify IP_3_R2-mediated ER calcium release as a key suppressor of melanophagy, thereby sustaining pigmentation in melanocytes. Using in vitro (B16 murine melanoma cells, primary human melanocytes) and in vivo (zebrafish) models, the authors report that IP_3_R2 expression is positively correlated with pigmentation. They then investigate the impact of IP_3_R2 knockdown and find that IP_3_R2 silencing enhances the stability of melanogenic proteins, while also inducing autophagic degradation of melanosomes (i.e., melanophagy). Concomitantly, they find that IP_3_R2 silencing decreases mitochondrial calcium uptake, increases lysosomal calcium loading, and lowers lysosomal pH. They propose a pathway wherein in IP_3_R2 knockdown cells impaired mitochondrial calcium uptake induces the activation of AMPK-ULK1, and increased lysosomal calcium activates TRPML1 via TMEM165 and closer proximity interactions between ER and lysosomes, TFEB nuclear translocation, and upregulation of melanophagy-related genes, namely OPTN and RCHY1. The work is placed within the context of emerging roles of organelle calcium signaling in pigmentation biology, where extracellular calcium influx pathways are known regulators, but the contribution of ER-mitochondria-lysosome crosstalk to melanosome turnover remains largely unknown.

**Response:** We thank the Reviewer for appreciating our work and highlighting that the contribution of ER-mitochondria-lysosome crosstalk to melanosome turnover remains largely unappreciated.

**Major comments:**

**Comment 1-** The central finding is that IP_3_R2 knockdown induces melanophagy and reduces pigmentation. However, the manuscript does not identify any physiological or pathological context in which IP_3_R2 expression or activity is naturally downregulated in melanocytes. Without such context, the knockdown may represent an artificial perturbation that broadly alters ER calcium handling and triggers melanophagy as part of a general stress-induced autophagy response. This raises uncertainty about whether the pathway operates in vivo under normal or disease conditions. It would strengthen the study to identify upstream cues that reduce IP_3_R2 function and to test whether these also trigger melanophagy through the proposed mechanism.

**Response:** We thank the Reviewer for asking such an important question. The Reviewer asked to identify any physiological or pathological context in which IP_3_R2 expression is naturally downregulated in melanocytes. To address this question, in the revised manuscript, we analyzed publicly available microarray datasets comparing skin samples from Caucasian and African populations (Yin *et al*., Experimental Dermatology 2014). This unbiased analysis revealed considerably lower IP₃R2 expression in the Caucasian skin as compared to African skin (**Fig. 1L**). This data support a physiological correlation between IP₃R2 expression and pigmentation level, reinforcing the physiological relevance of the proposed pathway.

**Comment 2-** While the data link IP_3_R2 knockdown to decreased pigmentation and increased melanophagy, the causality between altered organelle calcium dynamics and the melanophagy induction is inferred from correlation and partial rescue experiments. More direct interventions in the proposed downstream pathways (e.g., acute mitochondrial calcium uptake restoration, lysosomal calcium buffering) would strengthen mechanistic claims.

**Response:** We appreciate the Reviewer’s recommendation on strengthening the mechanistic causality between organelle Ca²⁺ dynamics and melanophagy. As suggested, in the revised manuscript, we restored acute mitochondrial Ca²⁺ uptake by MCU over-expression in the IP₃R2 knockdown background, which resulted in a marked reduction in melanophagy along with increased mitochondrial Ca²⁺ uptake in comparison to control (**Fig 6I-L**). This data clearly demonstrates that downstream of IP₃R2 silencing mitochondrial Ca²⁺ restoration rescues the melanophagy phenotype thereby revealing a mechanistic causality between mitochondrial Ca²⁺ dynamics and melanophagy.

Similarly, to assess the causality between lysosomal Ca²⁺ dynamics and melanophagy, we silenced TMEM165 in the IP₃R2 knockdown background. Excitingly, upon TMEM165 knockdown we observed reduction in melanophagy, concomitant with decrease in lysosomal Ca²⁺ levels under IP₃R2 silencing conditions (**Supplementary Fig 7I-L**). Together, these direct manipulations support a causal role for altered organelle Ca²⁺ dynamics in driving melanophagy.

We believe that these experiments would have addressed the concern of the Reviewer. However, if there are any other specific experiments that the Reviewer would like us to perform, we would be happy to carry out them as well.

**Comment 3-** Zebrafish assays convincingly show altered pigmentation with altered IP_3_R2 levels, but do not connect this to in vivo melanophagy measurements or TRPML1/TFEB activity, which would link the cell biology to organismal phenotype more directly.

**Response:** We thank the Reviewer for appreciating our *in vivo* zenrafish experiments. Futher, we acknowledge the Reviewer’s point of linking the cellular mechanisms to organismal phenotypes *in vivo*. Therefore, as suggested, we activated TRPML1 in the zebrafish model system. In the revised manuscript, we investigated role of the TRPML1–TFEB axis in pigmentation *in vivo* by pharmacological activation of TRPML channels with MLSA1. The MLSA1 treatment resulted in a marked reduction in zebrafish pigmentation compared to vehicle-treated controls (**Fig. 8M**). This phenotypic change was further substantiated by quantitative melanin content assays, which confirmed a significant decrease in melanin levels following MLSA1 treatment (**Fig. 8M–N**). These *in vivo* findings support the involvement of TRPML1-mediated lysosomal signaling in pigmentation regulation.

**Comment 4-** The work suggests therapeutic potential for pigmentary disorders, but no disease models are tested. It is unclear whether the observed mechanisms operate under physiological stressors.

**Response:** We appreciate the Reviewer’s comment regarding physiological relevance and disease context. As addressed in Comment 1, we examined publicly available human skin microarray datasets for IP₃R2 expression in Caucasian and African population. This analysis revealed a positive correlation between IP₃R2 expression and human skin pigmentation, supporting that modulation of IP₃R2 occurs under physiological conditions rather than representing an artificial perturbation.

While formal pigmentary disease models were not examined in this study, the observed correlation between IP₃R2 expression and physiological pigmentation differences along with our robust *in vivo* zebrafish data suggests that IP₃R2 plays an important role in physiological pigmentation. As highlighted by Reviewer 1 and Reviewer 3, the manuscript is already too long. Therefore, we plan to delineate the precise role of IP₃R2 in pigmentary disorders as an independent study.

**Comment 5-** The paradox between the observed enhanced stability of melanogenic proteins and increased melanophagy is insufficiently addressed. DCT, Tyrosinase and GP100 are all melanosome-associated and their stability or degradation is in prior literature often interpreted as reflecting melanosome biogenesis and turnover. This discrepancy needs to be resolved, as it complicates interpretation of melanophagy assays.

**Response:** We appreciate the Reviewer’s careful consideration of this apparent paradox. This point was also raised by Reviewer 1. We have addressed the query in detail in response to Comment 4 of Reviewer 1. Briefly, the enhanced stability of melanosome-associated proteins reflects impaired proteasomal degradation and prolonged protein half-life, while the concurrent increase in melanophagy represents a compensatory turnover mechanism for degrading such dysfunctional melanosomes.

Thus, increased melanophagy and apparent stabilization of melanogenic proteins are not contradictory but instead represent parallel outcomes of disrupted proteostasis. This interpretation is supported by our proteasomal inhibition experiments (**Fig 4A-H**) and autophagy analyses (**Fig 4I-P**), which collectively reconcile the observed protein stability with enhanced melanosome turnover.

**Comment 6-** The authors propose that mitophagy and ER-phagy are reduced in IP_3_R2 knockdown cells, suggesting specific induction of melanophagy, but the rationale for why increased autophagic flux only targets melanosomes is insufficiently addressed. Also, these conclusions are solely based on Keima assays, and positive controls for mitophagy and ER-phagy are lacking.

**Response:** We appreciate the Reviewer’s critical assessment of the specificity of autophagic targeting in the IP₃R2 knockdown condition and the need for appropriate validation controls. In the revised manuscript, we have repeated both the mitophagy and ER-phagy assays with well-established positive controls. Carbonyl cyanide-p-trifluoromethoxyphenylhydrazone (FCCP) was employed as a positive control to robustly induce mitophagy (**Supplementary Fig 4E-F**), while 4-phenylbutyric acid (4PBA) was used as a positive control for ER-phagy/reticulophagy (**Supplementary Fig 4G-H**). Secondly, we have validated the microscopy data with biochemical assays by examining levels of ER (**Fig 4E-H**) and mitochondria resident protein MCU (**shown below**).

To provide a mechanistic rationale for the specific induction of melanophagy, we examined recently identified regulators of melanophagy, RCHY1 and OPTN (Lee *et al*., PNAS 2024). Bioinformatic analysis identified multiple TFEB binding sites on the promoters of both genes, which was supported by increased RCHY1 and OPTN expression following IP₃R2 knockdown. Further, in the revised manuscript, we performed additional loss-of-function experiments to demonstrate that co-silencing IP_3_R2 along with RCHY1 or OPTN significantly reduced melanophagy flux compared to IP₃R2 knockdown alone (**Fig. 9H–K**). Taken together, these data explain why enhanced autophagic flux downstream of IP₃R2 silencing is preferentially directed toward melanosomes.

**Comment 7**- The melanophagy probes are novel and validated with rapamycin/bafilomycin, but quantitative calibration of GFP/mCherry or Keima signal to actual lysosomal delivery rates is missing; photobleaching, pH heterogeneity (incl., observed decrease in lysosomal pH), and melanin autofluorescence (see below) could confound ratios. Also, side-by-side comparison with other melanophagy detection approaches (e.g., colocalization of melanosomes with LC3) is lacking.

**Response:** We appreciate the Reviewer’s careful evaluation of the melanophagy probes and the potential technical confounders. In the revised manuscript, we have performed a variety of experiments to further characterize and validate the probes. First of all, the melanophagy detection ratiometric probes (mCherry–Tyrosinase–eGFP and tyrosinase mKeimaN1) are built on well-established and extensively validated backbones. Further, we used appropriate controls (empty vectors/non-targeting siRNAs/vehicle controls) in all experiments to analyze the relative fluorescence changes in the test condition v/s control. The confounding factors, if any, should be present for both test and control. Therefore, we initially did not perform side-by-side comparison with other melanophagy detection approaches.

In the revised manuscript, as suggested by the reviewer, we employed additional melanophagy detection strategies to further strengthen our findings. Specifically, we used Retagliptin phosphate (RTG), a well-established selective inducer of melanophagy, and observed a marked increase in melanophagy using the mCherry–Tyrosinase–eGFP melanophagy probe (**Supplementary Fig 2G-H**). Additionally, we performed independent validation by assessing colocalization of the melanosome (recognized by anti-HMB45 ab that identifies melanosomal structural protein GP100) with LC3 (**Supplementary Fig 3A-B**). This analysis revealed a significant increase in melanosomes colocalization with LC3 upon IP₃R2 silencing compared to control conditions. Further, to minimize the contribution of melanin autofluorescence, non-transfected cells were imaged under identical settings, and background signals obtained from these cells were subtracted during fluorescence quantitation from all acquired images. Potential effects of photobleaching and pH heterogeneity were minimized by uniform acquisition parameters and ratiometric analysis. Taken together, we believe these complementary approaches address the Reviewer’s concerns and reinforce the robustness of our melanophagy measurements.

**Comment 8-** Melanosomes exhibit broad autofluorescence, particularly upon excitation at 405-488 nm and extending into the red channel. This signal can overlap with the detection ranges for GFP, mCherry, and mKeima reporters, potentially confounding quantitative readouts unless appropriate controls (e.g., untransfected cells, spectral unmixing) are used. Throughout this manuscript, it is not addressed how melanosome autofluorescence was controlled for or excluded in the reported fluorescence measurements.

**Response:** We apologize to the Reviewer for not clearly stating that melanosome autofluorescence was controlled by imaging non-transfected cells under identical settings, and these background signals were subtracted during quantitation from the acquired images. Specifically, to rigorously control this issue, autofluorescence was systematically evaluated using non-transfected control cells imaged under identical excitation and emission settings used for GFP, mCherry, and mKeima reporters. These controls allowed us to define the baseline autofluorescence profile arising from melanosomes across the relevant spectral ranges. These details are included in the methods section.

**Comment 9-** While OPTN and RCHY1 expression is elevated upon IP_3_R2 knockdown, functional engagement (e.g., OPTN localization to melanosomes, melanosome ubiquitination by RCHY1), or necessity (e.g., siRNA knockdown of these in the IP_3_R2-deficient background), are not tested.

**Response:** We appreciate the Reviewer’s point on establishing necessity of OPTN and RCHY1 in IP₃R2 knockdown–induced melanophagy. In the revised manuscript, we performed targeted loss of function analyses for both OPTN and RCHY1 in the IP₃R2-deficient background. We assessed melanophagy using the mCherry–Tyrosinase–eGFP melanophagy probe following co-silencing of IP₃R2 with either OPTN or RCHY1. Quantitative analysis revealed a significant reduction in melanophagy flux upon co-silencing of either gene compared to IP₃R2 silencing alone (**Fig. 9H–K**). These findings establish the functional requirement of OPTN and RCHY1 downstream of IP₃R2 loss to drive melanophagy. Since functional engagement of OPTN and RCHY1 on melanosomes is already well-established (Lee *et al.* PNAS 2024 and Park *et al.* Autophagy 2024), we have not repeated these experiments. Taken together, our data demonstrates that OPTN and RCHY1 are not only overexpressed but also act as critical mediators of melanophagy downstream of IP₃R2 silencing.

**Comment 10-** While siRNA/shRNA efficacy is shown, functional rescue with pore-dead mutants sometimes fails to return to control values. The possibility of partial off-target or compensatory effects is not fully excluded.

**Response:** We thank the Reviewer for raising for this point. In this study, we employed pore-dead mutants of IP₃R2 (IP₃R2-M) and TRPML1 (TRPML1-M), both of them are well characterized, widely validated and extensively used by a number of leading groups in the field. Upon meticulous literature analysis, we came across multiple studies wherein partial rescue effect was reported with these pore-dead mutants. Therefore, we believe it is not surprising that we are also observing partial rescue in some of our assays.

Actually, it is important to note that we observe rescue of the function and phenotype in every single experiment carried out with the mutants. We agree with the Reviewer that the extent of rescue is not up to control levels in few experiments. This can be attributed to the differences in the extend of expression of mutants across different experiments. However, we have validated the results with multiple independent approaches. Collectively, the use of multiple independent approaches along with genetic silencing, pharmacological inhibition/activation supports the specificity of the observed phenotypes.

**Comment 11-** The mitochondrial and lysosomal calcium measurements are largely endpoint peak quantifications; kinetic analyses and buffering capacity measurements would provide more mechanistic depth, especially for the TMEM165 contribution. Also, TMEM165 necessity for melanophagy induction upon IP_3_R2 knockdown has not been directly addressed.

**Response:** We appreciate the Reviewer’s request for greater mechanistic depth regarding organelle Ca²⁺ dynamics and the specific contribution of TMEM165. Consistent with this, we had previously demonstrated that TMEM165 silencing decreases lysosomal Ca²⁺ levels using Oregon BAPTA–dextran–based measurements (**Supplementary Fig 7C-D**), establishing its role in regulating lysosomal Ca²⁺ buffering. Building on this, in the revised manuscript, we performed kinetic analyses of lysosomal Ca²⁺ levels following IP₃R2 and TMEM165 silencing. These kinetic analyses validated our end point measurements that IP₃R2 knockdown leads to increase in lysosomal Ca²⁺ levels, whereas TMEM165 silencing results in decrease in lysosomal Ca²⁺ content in comparison to control. Therefore, highlighting distinct and opposing effects of IP₃R2 and TMEM165 on lysosomal Ca²⁺ kinetics. This additional data is provided below for the ease of reference:

Further, we directly evaluated the necessity of TMEM165 for melanophagy induction in the IP₃R2-deficient background. TMEM165 knockdown alone resulted in a significant reduction in melanophagy (**Supplementary Fig 7G-H**). Further, co-silencing of TMEM165 with IP₃R2 also attenuated melanophagy compared to IP₃R2 knockdown alone (**Supplementary Fig 7K-L**). Collectively, these kinetic Ca²⁺ assays and genetic loss-of-function analyses provide mechanistic depth to the organelle Ca²⁺ measurements and establish TMEM165 as a critical regulator of melanophagy downstream of IP₃R2 silencing.

**Comment 12-** The proximity ligation assay between VAP-A and LAMP1 is interpreted as showing increased ER-lysosome contacts in IP_3_R2 knockdown cells. However, additional controls are needed and quantitative TEM should be included to substantiate changes in organelle contact frequency and distance.

**Response:** We thank the Reviewer’s for his/her emphasis on strengthening the validation of the proximity ligation assay (PLA) findings and on providing ultrastructural evidence to support altered organelle interactions. The PLA data revealed a significant increase in VAP-A–LAMP1 interaction signals in IP₃R2-silenced cells compared to control conditions (**Fig. 7L–M**). In the revised manuscript, this increase was not observed upon treatment with bafilomycin A1, a specific inhibitor of lysosomal acidification, or when one of the primary antibodies was omitted, confirming the specificity of the PLA signal (**Fig. 7L–M**). These controls support the interpretation that IP₃R2 downregulation enhances ER–lysosome interactions.

To further substantiate the changes in organelle contact frequency and distance, we performed ultrastructural analyses using transmission electron microscopy (TEM). The quantitative TEM measurements revealed no significant change in the frequency of ER–mitochondria or ER–lysosome contacts upon IP₃R2 silencing (**Fig. 7N–P**). Similarly, ER–mitochondria distances remained unchanged. However, we observed a significant reduction in the distance between the ER and lysosomes in IP₃R2 knockdown cells compared to control (**Fig. 7N, 7Q–R**). Together, these complementary approaches demonstrate that IP₃R2 silencing specifically increases ER–lysosome proximity without altering overall contact frequency, thereby strengthening the conclusion that IP₃R2 regulates ER–lysosome coupling.

**Comment 13-** Some assays report small biological n (e.g., three independent experiments with relatively small per-condition cell counts).

**Response:** We appreciate the Reviewer’s comment regarding sample size. All experiments were performed with a minimum of three independent biological replicates, which is consistent with standard practice in the field. For imaging-based assays, multiple fields of view and cells were analyzed per condition in each independent experiment, and quantitative analyses were performed on pooled data across replicates. As suggested by the Reviewer, we have increased the cell numbers in some experiments. The detailed information on biological replicates and cell numbers analyzed is provided in the respective figure legends.

**Minor comments:**

**Comment 1-** The title "IP_3_R2-mediated inter-organelle Ca^2+^ signaling orchestrates melanophagy" could be misread as indicating IP_3_R2 'promotes' melanophagy; consider rewording to make clear that IP_3_R2 suppresses melanophagy to maintain pigmentation. Similarly, the running title "IP_3_R2 negatively regulates melanophagy" would be clearer as "IP_3_R2 suppresses melanophagy".

**Response:** As suggested by the Reviewer, we have modified the title and running title in the revised manuscript.

**Comment 2-** Unify the framing of "positively regulates pigmentation" vs. "negatively regulates melanophagy" in the Introduction/Discussion.

Response: As recommended, we have unified the framing in the suggested sections.

**Comment 3-** Adding schematic flow diagrams summarizing each pathway at the end of relevant results (figure) sections could help accessibility.

**Response:** We appreciate the Reviewer’s suggestion to improve accessibility of the presented pathways. Accordingly, we have included schematic diagrams at the end of the relevant figures. These schematics summarize: (i) ER–mitochondria interactions in the context of melanophagy (**Fig. 6P**); (ii) differences in Ca²⁺ and pH regulation between wild-type and IP₃R2-silenced cells (**Fig. 7S**); and (iii) TRPML1-mediated Ca²⁺ release driving melanophagy via TFEB translocation (**Fig. 9L**). Together, these diagrams provide a concise visual overview of the key mechanistic pathways described in the study.

**Comment 4-** While the introduction summarizes extracellular calcium signaling in pigmentation, there is less coverage of recent work on selective autophagy of other lysosome-related organelles (e.g., platelet dense granules, lytic granules), which could provide broader mechanistic context.

**Response:** As suggested by the Reviewer, we have discussed selective autophagy of other lysosome-related organelles in the introduction.

**Reviewer #2** (Significance (Required)):

This study addresses an important gap in pigmentation biology by identifying IP_3_R2-mediated ER calcium release as a suppressor of melanophagy and a positive regulator of pigmentation. The strongest aspects are the integration of in vitro and in vivo models, the multi-faceted mechanistic exploration linking altered organelle calcium dynamics to selective melanosome turnover, and the development of novel ratiometric fluorescent probes for live-cell melanophagy measurement. Conceptually, the work extends prior literature that has focused on extracellular calcium influx and melanosome biogenesis, revealing a new inter-organelle calcium signaling module that controls melanosome degradation via AMPK-ULK1 and TMEM165-TRPML1-TFEB pathways.

However, several limitations reduce the strength of the mechanistic claims. Some key pathway steps are inferred from correlation and partial rescue rather than direct necessity/sufficiency tests (e.g., mitochondrial calcium uptake restoration, lysosomal calcium buffering). The paradoxical observation that IP_3_R2 knockdown both increases melanophagy and stabilizes melanosome-resident protein (DCT, Tyrosinase, GP100) is not resolved, complicating interpretation of the melanophagy assays. The specificity for melanophagy over other selective autophagy pathways is asserted but not fully explained mechanistically, and positive controls for mitophagy/ER-phagy are missing. Potential technical confounds, such as melanin autofluorescence in the detection ranges of GFP, mCherry, and mKeima, are not explicitly addressed and alternative assays for these key data were insufficiently employed. In vivo results do not yet connect altered pigmentation to melanophagy readouts or downstream TRPML1/TFEB activation. Importantly, the study does not identify any physiological or pathological scenario in which IP_3_R2 expression or activity is naturally reduced in melanocytes. In the absence of such upstream cues, IP_3_R2 knockdown may represent an artificial perturbation that triggers melanophagy as part of a broader stress-induced autophagy response, raising questions about the in vivo relevance of the proposed pathway.

The work's primary audience is specialized, cell biologists, autophagy researchers, and pigmentation/skin biology specialists, but the mechanistic framework on organelle crosstalk and selective autophagy will interest a broader basic research readership, including those studying lysosome-related organelles in other systems. The ratiometric probes could be adapted for future melanophagy research, and the pathway insights may guide translational studies in pigmentary disorders or melanoma. My expertise is in mitochondrial and lysosomal calcium signaling, autophagy, and microscopy-based functional assays; I do not have detailed expertise in zebrafish developmental genetics, though the phenotypic analysis appears sound.

**Response:** We thank the Reviewer for appreciating our work and stating that our study “addresses an important gap in pigmentation biology”. Further, we thank him/her for believing that this work will be of interest to a broad basic research readership. Moreover, we thank him/her for valuing the importance and potential significance of the ratio-metric melanophagy probes generated in this study. Finally, we acknowledge the Reviewer’s constructive feedback on our study, which has helped us in enhancing the quality of our manuscript. We have performed variety of additional *in vitro* experiments, *in vivo zebrafish studies* and have significantly revised the manuscript to address all the comments of the Reviewer.

**Reviewer #3** (Evidence, reproducibility and clarity (Required)):

This is a robust and extensive study showing that IP_3_R2 selectively initiates a calcium signalling pathway leading to melanophagy, that is the degradation of melanosomes. This reduces pigmentation and UV light protection. A strength of the paper is that it combines detailed cellular studies with in viva studies in the zebrafish model. They show that knockdown of IP_3_R2 reverses this process perhaps leading to a strategy to enhance melanosome number and hence to afford protection from UV irradiation. The authors use a battery of fluorescent probes (mainly genetically encoded reporters) in investigate the signalling cascade leading to melanophagy or its reduction. This involves reports for a number of different organelles involved in this process. The experiments are generally well performed with clear controls for the probes in many cases. My main issue is the panels contain too much data which may obscure the message, and a good deal could be moved to supplementary data. The manuscript investigates many mechanisms in distinct organelles which is remarkable for a two author paper. Particularly interesting was the design of novel fluorescent protein reporters for melanophagy itself. One area not explored is ion fluxes across melanosomes themselves which are lysosome-related organelles and may exhibit similar properties and signalsomes of lysosomes.

Specifically, the authors show that a REDUCTION of IP_3_R2-mediated calcium release leads to a calcium flux from the ER by a different mechanism (possibly via TMBIM6). This increases calcium loading of the lysosome via TMEM165, at the expense of calcium transfer to mitochondria, and an acidification.

This leads to TRPML1 activation and the lysosomal calcium release activates TFEB translocation to the nucleus increases the transcription of autophagy/melanophagy genes and activation of the AMPK-ULK1 pathway (rather than mTOR). This is a complex pathway and evidence is presented for many of the steps involved.

This is a tour de force investigating organelle communication during the process of melanophagy, that is little understood. It highlights many important organelle ion transport events that are important findings in their own right. For example, the importance of TMEM165 in calcium filling of lysosomes.

**Response:** We thank the Reviewer for appreciating our study and thinking that it is a robust and extensive study in a highly understudied area. We appreciate the Reviewer’s acknowledgement that our manuscript combines detailed cellular studies with *in vivo* studies in the zebrafish model. Further, we thank the Reviewer for his/her constructive feedback on our work.

**Major points:**

**Comment 1-** The authors state that TPC activation does not activate TFEB translocation the nucleus. This is now not the case and should be at least looked at. What is the role of endolysosomal channels on the melanosomes themselves in melanophagy.

**Response:** We appreciate the Reviewer’s comment regarding the potential contribution of TPC channels to TFEB activation and melanophagy. In the revised manuscript, we assessed Ca²⁺ release from TPC2 under IP₃R2 knockdown conditions using the selective TPC2 agonist TPC2-A1-N (**Supplementary Fig 9G-H**). Additionally, we evaluated TFEB nuclear translocation following TPC2-mediated Ca²⁺ release using TPC2-A1-N (**Supplementary Fig 9I-J**). Our analyses revealed no significant differences in TPC2 activity or TFEB nuclear translocation upon IP₃R2 silencing compared to control conditions. These findings suggest that, in our system, TPC2-mediated Ca²⁺ signaling does not contribute significantly to TFEB activation or melanophagy downstream of IP₃R2 silencing, indicating a more prominent role for TRPML1-dependent Ca²⁺ signaling in this context.

**Comment 2-** How does reduction in IP_3_R2 mediated calcium fluxes enhance lysosomal acidity?

**Response:** We thank the Reviewer’s question regarding the mechanistic link between reduced IP₃R2-mediated Ca²⁺ flux and enhanced lysosomal acidity. In the revised manuscript, we show that IP₃R2 silencing results in a significant upregulation of the lysosomal proton pump H⁺-ATPase subunits: ATPV0D1 and ATP6V1H (**Supplementary Fig 6E-F**). Increased H⁺-ATPase expression is expected to promote proton influx into the lysosomal lumen, thereby enhancing lysosomal acidification. These findings provide a mechanistic basis for how IP₃R2 silencing can drive increased lysosomal acidity.

**Comment 3-**	What mediates the ER source for calcium filling of lysosomes?

**Response:** We appreciate the Reviewer’s interest in the mechanism underlying ER to lysosome Ca²⁺ transfer. Recently, an independent study also reported that IP₃R2 silencing enhances lysosomal Ca²⁺ levels and lysosomal Ca²⁺ release (Zheng *et al.* Cell 2022). Literature suggests that lysosomal Ca²⁺ refilling is depend on Ca²⁺ fluxes originating from the endoplasmic reticulum, particularly through ER Ca²⁺ leak pathways at ER–lysosome contact sites. In this context, ER-resident Ca²⁺ leak channels such as TMBIM6 (also known as Bax inhibitor-1) play an important role in maintaining basal cytosolic Ca²⁺ levels that can be subsequently taken up by lysosomes (Kim *et al.* Autophagy 2020). TMBIM6-mediated Ca²⁺ leak from the ER provides a continuous, low-level Ca²⁺ source that supports lysosomal Ca²⁺ loading, (Kim *et al.* Autophagy 2020). This mechanism allows lysosomes to replenish their Ca²⁺ stores via Ca²⁺ uptake systems operating at ER–lysosome contact sites. Thus, ER Ca²⁺ leak channels represent a key conduit linking ER Ca²⁺ homeostasis to lysosomal Ca²⁺ filling and function.

Recently, lysosome localized TMEM165 was identified to play an important role in Ca²⁺ filling of lysosomes (Zajac *et al*. Science Advances 2024). Here, in our study, we observe that TMEM165 drives lysosomal Ca²⁺ influx in melanocytes.

**Comment 4-** Oregon-green-dextran is not a great probe for lysosomal calcium. Its Kd is 170nM and even in the acidic environment this may be lowered to low micromolar which may not be great for measuring changes around luminal concentrations of around 500uM. Additionally, it is usual to correct for pH effects simultaneously since the dye is also a pH reporter and has been used as such. However, I take the point that they still see an increase in fluorescence whilst pH falls probably indicating an increase in luminal lysosomal calcium confirmed by increased perilysosomal calcium.

**Response:** We thank the Reviewer for the careful and balanced assessment of the Oregon Green–dextran measurements. We appreciate the acknowledgment that, despite the known limitations of this probe and its pH sensitivity, the observed increase in fluorescence concurrent with reduced lysosomal pH is consistent with elevated luminal lysosomal Ca²⁺ levels. We are grateful for this positive interpretation, which strengthens our conclusions when considered alongside the large amount of supporting data.

**Comment 5-** The major point is to reduce the number of main data panels with consigment of some controls perhaps to supplementary. This would increase the comprehensibility of the paper.

**Response:** We thank the Reviewer for this constructive and positive suggestion. We appreciate the emphasis on reducing the data in the main figures. Therefore, as suggested, we have moved considerable data to the supplementary figures. However, due to the additional experiments performed to address the concerns of other Reviewers, the main data panels may still look little busy. We sincerely think that the Reviewer would understand our situation.

**Minor points**

**Comment 1-** Fig 10 needs a clear legend with symbols in the diagram explained. eg ER calcium release proteins.

**Response:** We thank the Reviewer for this helpful and constructive comment. Therefore, we have revised the **Figure 10** legend to clearly explain all symbols used in the schematic illustration.

**Reviewer #3** (Significance (Required)):

This is a tour de force investigating organelle communication during the process of melanophagy, that is little understood. It highlights many important organelle ion transport events that are important findings in their own right. For example, the importance of TMEM165 in calcium filling of lysosomes.

**Response:** We sincerely thank the Reviewer for considering our work as “a tour de force investigation” and appreciating that our study presents several important organelle ion transport events.
