# Supplementary material for: IP3R2-mediated inter-organelle calcium signaling suppresses melanosome degradation
Source: PLoS Biol. 2026 Aug 25;24(8):e3003971. doi: 10.1371/journal.pbio.3003971 (PMC13533435; doi:10.1371/journal.pbio.3003971)
Supplement: Supplementary file 5 [file pbio.3003971.s018.docx]

Thank you for your patience while we considered via Review Commons your revised manuscript entitled "IP_3_R2 mediated inter-organelle Ca^2+^ signaling suppresses melanophagy" for publication as a Research Article at PLOS Biology. Your revised study has been evaluated by the PLOS Biology editors, an Academic Editor and the three original reviewers.

The reviews are attached below. As you will see, all the reviewers appreciate the improvements made during the revision, however they still raise several remaining points that would need to be addressed before we can consider the manuscript for publication. Reviewer 1 asks for several clarifications and also thinks that the experiment in Fig. 8 showing that IP_3_R2 and TRPML1 knockdown decreases MLSA1 should be repeated using bafilomycin or GCN, given that MLSA1 is a TRPML agonist. Reviewer 2 thinks that the limitations of the study should be clearly discussed, and that the mechanistic shortcomings should be addressed by clearly indicating what findings are experimentally supported vs others that are correlative, prioritising the strongest and most rigorously supported conclusions and discussing more carefully the proposed proteasome-associated mechanism. In addition, this reviewer requests the rescue of mitochondrial Ca^2+^ uptake or direct manipulation of lysosomal Ca^2+^ buffering and additional streamlining of the text to improve the flow. Finally, Reviewer 3 asks for the clarification of one point.

In light of the reviews, we would like to invite you to revise the work to thoroughly address the reviewers' reports. We would also want to note that, after discussing the reviews with the Academic Editor, we think that Reviewer 2's request on the rescue of mitochondrial Ca^2+^ uptake or direct manipulation of lysosomal Ca^2+^ buffering is essential for publication.

***Response:*** *First of all, we would like to thank you, the Academic Editor and the Reviewers for appreciating the earlier revised manuscript. We also thank you and the Academic Editor for clearly outlining the experiments that are essential for considering the revised manuscript for publication. We are glad to update that we have performed all the suggested experiments. Briefly, as recommended by the Reviewer 1, we have performed lysosomal calcium experiments in TRPML1 silenced cells with GPN (instead of MLSA1). Further, as requested by the Reviewer 2, we have performed both the rescue of mitochondrial Ca^2+^ uptake and direct manipulation of lysosomal Ca^2+^ buffering by carrying out rescue experiments with MCU and by enhancing lysosomal Ca^2+^ buffering with TMEM165 overexpression respectively. These experiments have robustly strengthen our manuscript by clearly demonstrating a direct role of mitochondrial and lysosomal Ca2+ dynamics in melanophagy. Finally, we have clarified one point raised by the Reviewer 3.*

*Further, we thank all three Reviewers for appreciating our revised manuscript and for sharing constructive feedback to further enhance the quality of our work. It is important to highlight that we have performed all the suggested experiments and have modified the text as recommended by the Reviewers. Please find below the detailed point wise response to the Reviewers comments.*

**Reviewer 1:**

The revised manuscript has been much improved since the first submission. Nevertheless, some questions remain as follows:

**Response:** We thank the Reviewer for highlighting that the revised manuscript is much improved since the original submission. We also thank the Reviewer for the further constructive feedback. In the this revised version, we have addressed all the comments of the Reviewer by performing additional experiments and by modifying the text as suggested.

**Specific Comments:**

1. My comment about figure panels 1F and 1J should actually have been 1H and 1J. I find it difficult to assess the relative effect of αMSH on IP_3_R2 expression as presented.

**Response:** We sincerely thank the Reviewer for sharing the actual figure panel numbers, which has helped us improve the clarity. We would like to respectfully clarify that the experiments presented in Supplementary Figure1E and Supplementary Figure1G (previously Fig 1H and Fig 1J) were designed to demonstrate the knockdown efficiency of siRNA targeting IP_3_R2 in two distinct model systems (LD-based pigmentation model and α-MSH–induced pigmentation model respectively), and were not intended to illustrate the effect of αMSH on IP_3_R2 expression. Therefore, we have separated these data sets because these are two independent experimental conditions.

2. Unfortunately, my 2nd comment was also less than clear. While I do acknowledge that you measure IP_3_R mRNA, the relationship between mRNA and protein levels is rarely truly linear. Is IP_3_R2 the predominant isoform at the protein level? Closely related, rescue experiments in Fig 6 show that IP_3_R2 overexpression rescues a mitochondrial phenotype after its knockdown. Would IP_3_R1 or IP_3_R3 have been able to rescue also?

**Response:** We thank the Reviewer for the clarification. We fully agree that mRNA and protein levels do not always correlate linearly. To directly address this, we have interrogated the Human Protein Atlas database, which confirms that IP_3_R2 is indeed the predominant isoform at the protein level in melanocytes (**Supplementary Fig 1A**).

Regarding isoform specificity in the rescue experiments, we performed additional experiments overexpressing IP_3_R1 or IP_3_R3 in shIP_3_R2 stable knockdown cells and measured mitochondrial calcium uptake using the CEPIA2mt reporter. As shown in the revised manuscript (**Supplementary Fig 6C-D**), overexpression of IP_3_R2 robustly rescued mitochondrial calcium levels following shIP_3_R2 knockdown (p<0.0001 compared to shIP_3_R2+pcDNA), whereas neither IP_3_R1 nor IP_3_R3 overexpression was able to rescue mitochondrial calcium levels, remaining statistically indistinguishable from the knockdown condition alone (ns, p>0.05). As expected, the shIP_3_R2 condition showed a significant reduction in mitochondrial calcium compared to shNT control (p<0.01). The data is also provided below for the ease of reference. These findings collectively and unambiguously demonstrate that the role of IP_3_R2 in regulating mitochondrial calcium homeostasis is isoform-specific and cannot be compensated by IP_3_R1 or IP_3_R3. This data further support the conclusion that IP_3_R2 plays a unique and non-redundant role in mediating mitochondrial calcium uptake in melanocytes.

3. While I agree with the authors that all 3 IP_3_R isoforms are in the ER. The ER is not a homogeneous space and there are numerous examples of differences in subcellular localization. Are there differences in their association with melanosomes? Lysosomes? The PM?

**Response:** We sincerely thank the Reviewer for this highly insightful and important comment. We agree that the ER is a highly heterogeneous and compartmentalized organelle, and isoform-specific enrichment at distinct ER subdomains is a well-recognized phenomenon. While we acknowledge that a comprehensive co-localization analysis with melanosomes, lysosomes, and the plasma membrane would be highly informative, we would like to respectfully draw the Reviewer's attention to the existing literature and our own expression data that provide important contextual support for the specificity of IP_3_R2 in melanocytes.

Importantly, interrogation of the Human Protein Atlas database confirms that IP_3_R2 is by far the predominant isoform expressed at the protein level in melanocytes, with IP_3_R1 and IP_3_R3 expressed at comparatively negligible levels (**Supplementary Fig 1A**). Given this striking predominance of IP_3_R2 in this specific cell type, it is highly plausible that IP_3_R2 represents the principal isoform available to populate ER subdomains juxtaposed to melanosomes, lysosomes, and the plasma membrane. The functional non-redundancy demonstrated by our rescue experiments, where neither IP_3_R1 nor IP_3_R3 overexpression could compensate for IP_3_R2 loss (**Supplementary Fig 6C-D**), further supports that IP_3_R2 is the dominant and functionally relevant isoform at these critical membrane contact sites in melanocytes. Going forward, the direct isoform-resolved co-localization studies would further strengthen this conclusion. However, it is beyond the scope of this study and we acknowledge it as an important avenue for future investigation in the discussion section of the revised manuscript.

4. Starting on page 24, there are a series of references to GFP/RFP ratio in reference to a probe that contains mCherry and eGFP. The authors should recognize that RFP is a very different protein from mCherry; as described, this is inaccurate.

**Response:** We thank the Reviewer for this careful and precise observation. We sincerely apologize for this inaccuracy in the original manuscript. The Reviewer is absolutely correct that RFP and mCherry are distinct fluorescent proteins and should not be used interchangeably. In the revised manuscript, we have carefully corrected all instances of "RFP" to "mCherry" throughout the text, starting from page 24 and across all subsequent references to the EGFP/mCherry ratio in the context of the mCherry-Tyrosinase-EGFP reporter system.

5. Figure 6N reports an increase in the p-AMPK/AMPK ratio. While I agree that it shows that, why does the total AMPK level go down while the amount of phosphorylation stays the same?

**Response:** We thank the Reviewer for this careful observation. We sincerely apologize for the confusion caused by the representative blot image presented in the original manuscript. Upon careful re-examination, we would like to clarify that the apparent decrease in total AMPK levels was not a true biological effect but rather a consequence of imperfect selection of representative blot image. In the revised manuscript, we have now replaced the representative western blot image with a more accurate and representative blot, as shown in the revised **Figure 5L**, which clearly demonstrates consistent and comparable total AMPK levels across both siNT and silP_3_R2 conditions, with tubulin confirming equal loading across all lanes. The revised representative blot is provided below for the ease of quick reference.

6. Throughout the article, the term "leaky channels" is used, however, they are typically referred to as "leak channels"

**Response:** We thank the Reviewer for the terminology correction. We sincerely apologize for the inaccurate use of the term "leaky channels". In the revised manuscript, we have carefully replaced all instances of "leaky channels" with the correct and widely accepted term "leak channels".

7. There is a claim that IP_3_R2 channels downregulate TRPML1 activity. However, given that these channels are in different organelles, have the authors considered the possibility that this relates to calcium loading in lysosomes?

**Response:** We thank the Reviewer for this thought-provoking comment. Yes, we agree with the Reviewer that the change in TRPML1 activity could be in part due to differences in lysosomal calcium loading. In the revised manuscript, we clearly demonstrate that IP_3_R2 knockdown increases calcium loading within lysosomes through the TMEM165 channel (**Supplementary Fig 9I-J**). This implies that under basal conditions, IP_3_R2 decreases lysosomal calcium loading. Additionally, TRPML1 activity is regulated by lysosomal pH and its activity is optimal under acidic lysosomal pH (Xiong *et al.*, Sci China Life Sci 2016). Since our data demonstrates that IP_3_R2 modulates lysosomal pH, the regulation of TRPML1 activity could be in part due to the changes in lysosomal pH. Therefore, we think that downstream of IP_3_R2 both lysosomal calcium loading and lysosomal pH can contribute to regulation of TRPML1 activity.

8. In figure 8E, F the authors demonstrate that IP_3_R2 and TRPML1 knockdown decreases MLSA1 stimulated calcium release. Since MLSA1 is a TRPML agonist, this is not appropriate. This experiment should be done with bafilomycin or GPN.

**Response:** We thank the Reviewer for this critical observation. We agree that the use of MLSA1, a TRPML agonist, in the context of simultaneous TRPML1 knockdown is not a good experimental design. We sincerely apologize for this experimental oversight. As suggested, in the revised manuscript we have performed this experiment using GPN. As expected, the combined knockdown of IP_3_R2 and TRPML1 leads to a significant increase in GPN-induced lysosomal Ca²⁺ extrusion compared to the control condition (**Fig 7E-F** of the revised manuscript). This revised data is shown below for quick reference.

9. In reference to Fig 8G-H, the authors wrote "comparison to the control condition" however, that is quite vague. What was it compared with?

**Response:** We thank the Reviewer for this careful observation. We sincerely apologize for the incomplete description in the original manuscript. To clarify, in revised **Figure 7G-H** (previously Fig. 8G-H), the comparison was made between silP_3_R2+siTRPML1 and siNT+siTRPML1 conditions. This clarification has now been explicitly stated in the revised manuscript.

**Reviewer 2:**

The revised manuscript is substantially strengthened relative to the prior version. The authors have added several important experiments, including additional validation of the melanophagy probes, autofluorescence controls, OPTN/RCHY1 loss-of-function analyses, TMEM165 loss-of-function experiments, positive controls for mitophagy and ER-phagy reporters, and PLA/TEM analyses of ER-lysosome proximity. These additions improve aspects of the technical rigor and mechanistic support for the central model. However, despite these improvements, I do not think all major concerns have been fully resolved. The manuscript remains mechanistically overextended, with several conclusions still relying heavily on indirect readouts, correlative relationships, or single-assay support. In some cases, the new data add additional mechanistic complexity without fully resolving the original interpretational concerns. More broadly, the study attempts to establish a very large interconnected pathway spanning ER calcium signaling, mitochondrial calcium uptake, bioenergetics, proteostasis, lysosomal calcium signaling, organelle contact sites, transcriptional regulation, and selective autophagy. While ambitious, the breadth of the proposed model ultimately exceeds the rigor with which several individual mechanistic links are established. In particular, the physiological relevance of IP_3_R2 downregulation, the proposed proteasome-melanophagy link, and the specificity of melanophagy induction remain insufficiently established for the level of mechanistic certainty claimed throughout the manuscript.

**Response:** We thank the Reviewer for highlighting that the revised manuscript is substantially strengthened relative to original submission. We also thank the Reviewer for appreciating that we have performed several important experiments in the first round of revision. Further, we thank the Reviewer for the constructive feedback. In the this revised version, we have addressed all the comments of the Reviewer by performing additional experiments and by modifying the text as suggested.

**Major comments:**

1.Physiological relevance of IP_3_R2 downregulation remains insufficiently established. The central experimental paradigm throughout the manuscript is IP_3_R2 knockdown. While the authors now include analysis of public transcriptomic datasets showing lower IP_3_R2 expression in lighter skin, this remains correlative and does not establish a physiological or pathological context in which IP_3_R2 activity is naturally reduced in melanocytes to trigger melanophagy. Given that IP_3_R2 is a major ER Ca^2+^ release channel, its silencing likely induces broad perturbations in ER Ca^2+^ handling, organelle communication, bioenergetics, and cellular stress pathways. Thus, it remains unclear whether the observed phenotype reflects a bonafide endogenous melanophagy-regulatory mechanism versus a broader stress response secondary to disrupted Ca^2+^ homeostasis. This limitation should be discussed much more explicitly, and claims regarding physiological relevance and translational potential should be tempered accordingly.

**Response:** We sincerely thank the Reviewer for this important critique. We acknowledge this limitation and agree that this needs more discussion. However, we would like to highlight several lines of evidence supporting the physiological relevance of IP_3_R2 downregulation. First, αMSH (α-melanocyte stimulating hormone, a physiological melanogenic stimulus) generates IP_3_ (Motiani *et al.*, EMBO J 2018; Tanwar *et al.*, JBC 2022) and induces Ca^2+^ release through IP_3_Rs (Tanwar *et al.*, PLoS Biology, 2024), and αMSH-stimulated pigmentation is significantly decreased upon IP_3_R2 silencing (data presented in this manuscript). Second, IP_3_R2 expression is directly correlated with pigmentation levels across two independent microarray datasets. IP_3_R2 levels are enhanced with increase in pigmentation in mouse melanocytes **(Fig 1A-B)**. Similarly, in primary human melanocytes, IP_3_R2 levels are augmented upon tyrosine-mediated hyperpigmentation and reduced upon PTU-driven hypopigmentation **(Fig 1C-E)**. Third, publicly available transcriptomic dataset comparing African and Caucasian skin demonstrates significantly lower IP_3_R2 expression in lighter skin **(Fig 1L)**. Fourth, pore-dead IP_3_R2 mutant (IP_3_R2-M) experiments confirm that Ca^2+^ channel activity is specifically required for its pro-pigmentary function (revised **Fig 5**). Fifth, *in vivo* zebrafish studies robustly recapitulate the *in vitro* observations at the organism level (**Fig 2**).

We nonetheless acknowledge the Reviewer's concern regarding potential broader ER Ca^2+^ perturbations secondary to IP_3_R2 knockdown. Therefore, we have discussed this in the revised manuscript and have tempered claims regarding translational potential.

2. The manuscript remains mechanistically diffuse and overextended. The revised version further expands the proposed signaling network connecting IP_3_R2 loss to proteasomal dysfunction, melanophagy, mitochondrial Ca^2+^ depletion, AMPK/ULK1 activation, altered lysosomal pH, TMEM165 function, ER-lysosome proximity, TRPML1 activation, TFEB nuclear translocation, and OPTN/RCHY1 induction. While many individual observations are interesting, not all mechanistic links are supported with comparable rigor. Importantly, the manuscript increasingly relies on accumulation of partially supported pathway relationships rather than deeply establishing individual mechanistic steps. In several instances, one indirect readout is used to support another inferred mechanism, creating a chain of conclusions where the overall certainty exceeds that of the underlying experimental evidence. The manuscript would therefore benefit from a clearer distinction between experimentally demonstrated findings versus inferred pathway relationships, and from a more focused mechanistic framing prioritizing the strongest and most rigorously supported conclusions.

**Response:** We sincerely thank the Reviewer for his constructive feedback to increase the rigor. This has further enhanced the quality of our manuscript. In the revised manuscript, we have restructured the mechanistic framework to prioritize the two central axes that are supported by multiple independent lines of evidence. First, the IP_3_R2-mitochondrial Ca^2+^-AMPK-ULK1 axis, which is supported by CEPIA2mt-based mitochondrial Ca^2+^ measurements; experiments with full-length and pore-dead mutant IP_3_R2 revealing that IP_3_R2 mediated Ca^2+^ release decreases melanophagy; MCU overexpression based rescue experiments demonstrating that the recovery of mitochondrial Ca^2+^ reverses IP_3_R2 silencing induced melanophagy; and direct phosphorylation assays for AMPK and ULK1 (revised **Fig. 4** and **Fig. 5**). Second, the IP_3_R2-lysosomal Ca^2+^-TRPML1-TFEB axis, supported by quantitative TEM and PLA-based ER-lysosome proximity analyses; TMEM165 gain and loss of function experiments coupled with direct lysosomal Ca^2+^ measurements; TRPML1 pharmacological manipulation substantiated with its genetic silencing & further validated with gain of function studies with either full length or mutant TRPML1; TFEB nuclear translocation assays (both microscopy and biochemical studies) coupled with OPTN/RCHY1 co-silencing experiments (revised **Fig. 6-8** and revised **Supplementary Fig 9**). In both these axes, the data is robustly validated by a variety of independent experimental approaches.

We acknowledge that the connection between proteasomal dysfunction and melanophagy induction requires more validation and we have now discussed this point in the revised manuscript. Further, we have moved several supporting analyses to the supplementary section to reduce mechanistic density in the main text. We believe that these revisions have substantially improve the mechanistic clarity and overall rigor of the manuscript.

3. The proposed proteasome-melanophagy connection remains insufficiently supported. A central conceptual issue remains unresolved: IP_3_R2 knockdown stabilizes melanogenic proteins (DCT, Tyrosinase, GP100) while simultaneously increasing melanophagy readouts. Because these proteins are integral melanosomal components commonly used as indicators of melanosome abundance or turnover, this creates significant interpretational ambiguity. The authors propose impaired proteasomal degradation followed by compensatory melanophagy, but the supporting evidence remains limited. In particular, the MG132 experiments are difficult to interpret mechanistically. The observation that MG132 treatment decreases GP100/DCT levels is counterintuitive for a proteasome inhibitor and does not directly demonstrate impaired proteasomal degradation following IP_3_R2 knockdown. Alternative explanations (incl. indirect stress effects, altered melanosome homeostasis, compensatory lysosomal/autophagic degradation, toxicity, or altered protein extraction/detection) remain plausible.

Furthermore, how do the authors explain the apparent increase in endogenous MCU protein levels under IP_3_R2 silencing conditions, which appears further enhanced following MG132 treatment (see Response to Rev 2_Comment 6)? This observation is mechanistically important because mitochondrial Ca^2+^ uptake is nevertheless reduced under these conditions, implying that impaired uptake is not simply attributable to reduced MCU abundance. Instead, the data may indicate compensatory upregulation of MCU, altered ER-mitochondria Ca^2+^ microdomain signaling, mitochondrial dysfunction, or broader proteostasis remodeling. The relationship between MCU expression, mitochondrial Ca^2+^ uptake capacity, and the proposed proteasome-associated mechanism should therefore be discussed more carefully (see below comment also).

Taken together, the current data do not yet provide sufficiently direct evidence to support the proposed proteasome-centered model. Direct proteasome activity measurements, ubiquitination analyses, or compartment-specific turnover assays would be needed to substantiate these conclusions more convincingly. At minimum, the proteasome-related conclusions should be substantially tempered.

**Response:** We sincerely thank the Reviewer for this critical evaluation. As suggested by the Reviewer, we have tempered the proteasome-related conclusions. Further, we have clearly stated that “In future, direct proteasome activity measurements and ubiquitination analyses of specific melanosomal substrates will be needed to substantiate this mechanism.”

Regarding MCU protein levels, we would like to apprise that MCU levels do not change significantly upon IP_3_R2 knockdown while it increases upon MG132 treatment in IP_3_R2 silenced cells. Please find below the updated western data (better representative) with statistical analysis. Further, we agree with the Reviewer that the decrease in mitochondrial Ca^2+^ uptake is most likely due to altered ER-mitochondria Ca^2+^ microdomain because IP_3_R2 silencing results in reduced ER Ca^2+^ release at this microdomain.

In summary, we have incorporated a carefully expanded discussion on proteasome-related data in the revised manuscript acknowledging that direct proteasome activity measurements and ubiquitination analyses will be necessary in future studies to substantiate the proposed proteasome-centered model.

4. Mechanistic causality remains incomplete despite additional experiments. The newly added TMEM165 co-silencing experiments and lysosomal Ca^2+^ kinetic analyses strengthen parts of the proposed pathway. However, several key mechanistic relationships remain largely associative. For example, the manuscript supports a role for altered mitochondrial and lysosomal Ca^2+^ dynamics in melanophagy induction, but direct necessity/sufficiency experiments remain limited. Rescue of mitochondrial Ca^2+^ uptake or direct manipulation of lysosomal Ca^2+^ buffering would substantially strengthen causal interpretation. While MCU overexpression partially addresses this point, unchanged or elevated endogenous MCU protein levels under IP_3_R2 knockdown conditions further suggest that altered mitochondrial Ca^2+^ uptake likely reflects changes in local ER-mitochondria Ca^2+^ transfer, channel regulation, mitochondrial state, or bioenergetics rather than simple limitation of uptake machinery abundance. As written, some pathway steps are presented with greater certainty than the data currently support.

**Response:** We sincerely thank the Reviewer for this constructive critique. We also thank the Reviewer for acknowledging that the newly added data strengthen parts of the proposed pathway. Further, we would like to respectfully highlight that our study now provides necessity/sufficiency evidence for the key mechanistic relationships proposed.

Regarding lysosomal Ca^2+^ dynamics, we have now established comprehensive causal evidence through multiple complementary approaches. For necessity, TMEM165 silencing alone significantly reduces lysosomal Ca^2+^ levels and consequently decreases melanophagy (revised **Supplementary Fig 9C-H)**. While TMEM165 co-silencing in IP_3_R2 knockdown conditions significantly rescues the IP_3_R2 knockdown-driven rise in lysosomal Ca^2+^ and correspondingly reduces melanophagy (revised **Supplementary Fig 9I-L)**. For sufficiency, we have now performed TMEM165 overexpression experiments to demonstrate that TMEM165 overexpression alone is sufficient to significantly increase intra-lysosomal Ca^2+^ levels (revised **Supplementary Fig 9M-N; Data is shown below as panel A and B)**. This data is in line with original discovery of TMEM165 as a lysosomal Ca^2+^ influx channel (Zajac *et al.* Science Advances, 2024). Importantly, TMEM165 overexpression is also sufficient to significantly increase the melanophagy (revised **Supplementary Fig 9O-P; Data is shown below as panel C and D)**. These experiments clearly demonstrate that TMEM165-mediated lysosomal Ca^2+^ loading is sufficient to drive melanophagy. Taken together, the comprehensive gain and loss of function experiments collectively establish robust causal necessity and sufficiency for the lysosomal Ca^2+^ dynamics in melanophagy induction (revised **Supplementary Fig 9**).

Regarding mitochondrial Ca^2+^ dynamics, we acknowledge the Reviewer's insightful observation that unchanged endogenous MCU protein levels under IP_3_R2 knockdown conditions suggest that reduced mitochondrial Ca^2+^ uptake could be due to altered ER-mitochondria Ca^2+^ microdomain. Nonetheless, MCU overexpression in IP_3_R2 silenced cells rescues both mitochondrial Ca^2+^ uptake and melanophagy (revised **Fig 5G-J**). This clearly demonstrate that mitochondrial Ca^2+^ uptake plays a critical role in melanophagy regulation. However, as suggested by the Reviewer, we agree that the altered mitochondrial Ca^2+^ uptake upon IP_3_R2 silencing could be an outcome of a combinatorial changes.

**B.**

**A.**

**C.**

**D.**

5. Specificity for melanophagy over broader stress-associated autophagy remains insufficiently explained. The addition of positive controls for mitophagy and ER-phagy reporters improves the rigor of these assays. However, the manuscript still does not adequately explain why broad perturbation of ER Ca^2+^ signaling and cellular bioenergetics would selectively induce melanosome degradation while suppressing or sparing other selective autophagy pathways. The current data support altered melanophagy-associated phenotypes, but the claim of mechanistic specificity for melanophagy remains stronger than the available evidence.

**Response:** We sincerely thank the Reviewer for this insightful comment. Further, we thank the Reviewer for acknowledging that our additional control experiments have enhanced the rigor of these assays. We understand the Reviewer’s concern regarding why melanophagy is specifically affected in our system. The most likely explanation could be that this is a cell-type specific phenomenon. Since melanocytes use a substantial amount of energy in melanosome biogenesis and melanogenesis (their prime physiological function), they may prefer to recycle melanosomes via melanophagy for new melanosome biogenesis. Similar equivalents are extensively reported in context of mitophagy wherein cells specifically recycle mitochondria via mitophagy for new mitochondria biogenesis to conserve the cellular resources. We have discussed this explanation in the revised manuscript.

Additionally, we would like to highlight independent lines of evidence supporting the relative selectivity of IP_3_R2 knockdown-induced autophagy towards melanosomes. First, our live-cell ratiometric melanophagy assays using two *de novo* generated probes, alongside well-established mitophagy and ER-phagy reporters, demonstrate that IP_3_R2 silencing preferentially induces melanophagy while mitophagy and ER-phagy are comparatively spared (revised **Supplementary Fig 5**). Second, transcriptional induction of OPTN and RCHY1, the selective melanophagy receptor and E3-ligase respectively, provides a specific molecular mechanism driving melanosome degradation. We nonetheless acknowledge that other alternative signaling modules may also contribute to the melanophagy selectivity.

6. Interpretation of the melanophagy probes still requires substantial caution. The authors now state that melanosome autofluorescence was controlled by imaging non-transfected cells under identical acquisition settings and subtracting background signals during quantitation. While this clarification is helpful, the issue remains incompletely resolved experimentally. Melanosome autofluorescence is highly heterogeneous, punctate, and biologically variable, particularly across conditions that themselves alter pigmentation, melanosome abundance, and lysosomal pH. Under such conditions, simple background subtraction may not be sufficient to exclude spectral contribution to GFP/mCherry/mKeima-based measurements. Importantly, representative autofluorescence control images, quantitative baseline fluorescence measurements, or spectral unmixing analyses are not provided.

More broadly, these probes remain pH-sensitive fluorescence reporters used in highly pigmented cells, while the proposed mechanism itself alters lysosomal pH. Thus, interpretation remains potentially influenced by lysosomal acidification state, melanosome autofluorescence, probe localization, and expression levels. The reporters are useful tools, but the manuscript should avoid presenting them as fully quantitative measures of melanophagy flux without additional calibration to actual lysosomal delivery or degradation rates.

**Response:** We thank the Reviewer for this comment. As suggested, we have included representative autofluorescence control images from non-transfected cells acquired under identical imaging settings across all experimental conditions, demonstrating negligible background signal in both EGFP and mCherry channels (**data is shown below in the top panel**) in the absence of probe expression. Similarly, imaging of non-transfected cells in the 488 nm and 543 nm channels (**data is shown below in the bottom panel**) confirms negligible autofluorescence under identical experimental conditions. These images are provided below for the ease of reference.

While we acknowledge the concerns with the fluorescence imaging using probes and concur that caution in interpretation is required, we do not agree with the Reviewer completely. A large volume of high quality literature suggests that using appropriate controls, a variety of fluorescent reporter systems can be reliably used in melanocytic cells (Gangi Setty et al. Nature 2008; Crawford et al. Science 2017; Bellono et al. eLife 2014; Ambrosio et al. PNAS 2016; Ramkumar *et al.* Autophagy, 2017; Ozdeslik et al. PNAS 2019; Magalhães Rebelo *et al.* Nature Comm 2026; Shiiba *et al.* Nature Comm 2026). Notably, few of these probes were used for novel discoveries including melanosome ion channels that regulate pH. In our study, we have used a variety of controls while characterizing and then employing these probes for measuring melanophagy levels. Secondly, we have complemented the key probe based datasets with biochemical studies and ultrastructural analysis.

To confirm probe localization, we performed co-localization analysis with the melanosomal marker HMB45. This analysis showed a strong overlap for both the mCherry-Tyrosinase-eGFP probe (PCC ~0.80, OC ~0.85) and the Tyrosinase-mKeimaN1 probe (PCC ~0.80, OC ~0.80), confirming probe targeting to melanosomes (revised **Supplementary Fig 3**). Next, we used autophagy inducer and inhibitor (positive and negative controls) to demonstrate that these probes can detect changes in the melanophagy levels (revised **Supplementary Fig 3**). Importantly, we used an established melanophagy inducer (RTG) as a positive control to demonstrate that these probes can efficiently detect change in the melanophagy levels (revised **Supplementary Fig 3**). Finally, regarding expression levels, as both probes are single-construct ratiometric reporters with fixed fluorophore stoichiometry, cell-to-cell differences in absolute expression are intrinsically normalized within the ratio itself. These clarifications have been incorporated into the revised manuscript. Additionally, we have discussed that these probes serve as semi-quantitative tools for assessing relative melanophagy rather than fully quantitative measures of melanophagy flux.

Importantly, our probe-based observations are independently corroborated by orthogonal biochemical assays and ultrastructural studies, which collectively strengthen the overall interpretation of melanophagy induction.

7. Evidence for endogenous melanophagy pathway engagement remains limited. The authors now include HMB45/LC3 colocalization analyses as supplementary evidence supporting melanophagy induction (Suppl. Fig. 3). While this addition is helpful, the image quality and analytical approach limit interpretability. In particular, insufficient resolution and reliance on global PCC measurements does not convincingly establish discrete autophagosomal association or engulfment of endogenous melanosomes.

The newly added OPTN/RCHY1 co-silencing experiments strengthen the argument that these factors contribute functionally downstream of IP_3_R2 loss. However, these analyses rely primarily on the same fluorescence reporter systems whose quantitative interpretation remains a central issue in the manuscript. In addition, direct evidence that endogenous melanophagy machinery is engaged under these conditions remains limited. The manuscript still lacks direct demonstration of OPTN/RCHY1 recruitment to melanosomes. While prior studies have implicated OPTN and RCHY1 in melanophagy, pathway engagement downstream of IP_3_R2 silencing in the present system remains inferred rather than directly demonstrated.

**Response:** We thank the Reviewer for acknowledging that our additional data is helpful in providing the evidence supporting melanophagy induction. Although we agree that a variety of other assays can be performed, we here have provided four independent line of evidences to conclude that endogenous melanophagy pathway is engaged downstream of IP_3_R2 silencing. We performed HMB45/LC3 co-localization analysis (revised **Supplementary Fig 4A-B**), which were substantiated by performing transmission electron microscopy (revised **Supplementary Fig. 5A-D**). This provides direct morphological evidence of melanosome degradation, which independently corroborates the co-localization data without solely relying on fluorescence-based measurements. Additionally, we performed classical biochemical assay (routinely used in the autophagy and organelle-phagy field) to demonstrate that IP_3_R2 induces melanophagy (revised **Supplementary Fig 4C-D**). Further, we demonstrate the engagement of established endogenous melanophagy pathway i.e. OPTN/RCHY1 axis upon IP_3_R2 silencing (revised **Fig. 8**). *Indeed, we thank the Reviewer for agreeing that “newly added OPTN/RCHY1 co-silencing experiments strengthen the argument that these factors contribute functionally downstream of IP_3_R2 loss”.* We think that collectively this data reveals the engagement of melanophagy pathway downstream of IP_3_R2 knockdown.

Regarding the global Pearson's Correlation Coefficient measurements. We have acknowledged this as a technical limitation in the revised text and have discussed the ultrastructural, biochemical, and molecular evidence that validates the confocal microscopy observations.

We also thank the Reviewer for acknowledging that the newly added OPTN/RCHY1 co-silencing experiments have strengthen our study. However, the direct demonstration of OPTN/RCHY1 recruitment to melanosomes is beyond the scope of this study. Already a prior study, in a highly reputed journal, has comprehensively established OPTN and RCHY1 as the selective melanophagy receptor and E3-ligase respectively (Reference: Lee *et al.* PNAS, 2024). Further, our functional co-silencing experiments demonstrate their critical role downstream of IP_3_R2. In the context of this study, our aim was to demonstrate the engagement of OPTN/RCHY1 axis upon IP_3_R2 silencing. We strongly believe that repeating the suggested experiments that were already performed by other group would lead to unnecessary delay in publication of our work as well as wastage of resources (human time and consumables + instrumentation access charges). We are confident that the Reviewer would appreciate our point.

**Minor comments:**

1. The newly added PLA controls and quantitative TEM analyses substantially strengthen the ER-lysosome proximity data. However, the observation that ER-lysosome distance decreases without a corresponding increase in contact frequency is conceptually interesting and deserves more detailed discussion.

**Response:** We sincerely thank the Reviewer for appreciating our TEM and PLA analyses. Our quantitative TEM analysis in the revised **Fig 6** demonstrates that ER-lysosome distance is significantly reduced upon IP_3_R2 knockdown, while the frequency of discrete ER-lysosome contact events remains statistically unchanged between conditions, indicating that proximity and contact number reflect distinct, dissociable aspects of organelle architecture in our system. As suggested by the Reviewer, we have discussed this point in the revised manuscript.

2. Some claims regarding therapeutic relevance for pigmentary disorders and melanoma remain speculative, as no disease model or physiological stress paradigm is examined.

**Response:** We acknowledge that therapeutic relevance claims remain speculative in the absence of direct disease model examination, and we have now tempered these claims in the revised manuscript.

3. The manuscript remains exceptionally dense, with many mechanistic branches introduced in rapid succession. Additional streamlining and prioritization of the central mechanistic findings would improve clarity and accessibility.

**Response:** We thank the Reviewer for this constructive suggestion. As suggested, in the revised manuscript, we have restructured the manuscript to prioritize the two core mechanistic axes: the IP_3_R2-mitochondrial Ca^2+^-AMPK-ULK1 axis and the IP_3_R2-lysosomal Ca^2+^-TRPML1-TFEB axis, as the central drivers of melanophagy induction. We believe that these revisions have substantially improved the clarity and accessibility of the manuscript while preserving its comprehensive mechanistic framework.

**Reviewer 3:**

Antony Galione – note that this reviewer has signed the review.
This manuscript had previously been refereed as a preprint before submission to PLOS Biology, and the authors have been joined by an additional author for this submission. The authors have outlined their responses to the pre-review and added new data to address the points made.

The principal finding is that silencing of the type 2 IP_3_ receptor evokes a pathway leading to melanophagy, where by melanosomes are degraded. Thus it is proposed that IP_3_R2-mediated calcium fluxes maintain the presence of melanosomes and hence pigmentation. The authors present a model by which inter-organelle calcium transfer between the ER, mitochondria and lysosomes play an important role in this process.

They have measured calcium in organelle compartments such as mitochondria and lysosomes and assessed Ca^2+^ loading and release in IP_3_R2 silenced cells and the effects of IP_3_ linked agonists or membrane permeant IP_3_. These experiments are generally convincing, but complex since the role of MCU, TMEM165, TRPML1 are all assessed either genetically or pharmacologically. These however, often rely on one KD experiment or one pharmacological agent.

In general I am satisfied with the responses to the referees for the preprint reviews (I was one of them) which were attached. It goes some way to strengthen what is a complex paper. The link with the zebrafish model in vivo is commended, and I note there is now data showing the effect of activating TRPML1 pharmacologically.

**Response:** We thank the Reviewer for appreciating our study and for approving that the revised manuscript has addressed the initial Reviewer’s comments. Further, we are glad to know that the Reviewer commended our manuscript for linking detailed cellular studies with *in vivo* studies in the zebrafish model.

I have one major question:

1. B16 were activated by histamine to produce a calcium response via IP_3_ presumably via H1 receptors. Does αMSH do the same?

**Response:** We sincerely thank the Reviewer for this important and insightful question. Yes, αMSH activates IP_3_R-mediated Ca²⁺ signaling in melanocytes just like histamine. Indeed, we and others have earlier demonstrated that αMSH generates IP_3_ (Maresca *et al.,* PCMR 2013; Motiani *et al.*, EMBO J 2018;) and triggers Ca²⁺ release through IP_3_ receptors in melanocytes (Motiani *et al.*, EMBO J 2018; Tanwar *et al.*, JBC 2022). Further, we recently revealed that αMSH stimulated IP_3_ generation subsequently drives mitochondrial Ca²⁺ uptake (Tanwar *et al.*, PLoS Biology, 2024).
