# Supplementary material for: IP3R2-mediated inter-organelle calcium signaling suppresses melanosome degradation
Source: PLoS Biol. 2026 Aug 25;24(8):e3003971. doi: 10.1371/journal.pbio.3003971 (PMC13533435; doi:10.1371/journal.pbio.3003971)
Supplement: Peer review history

# Author comment: Author response to Decision Letter 0

**DOI:** 10.1371/journal.pbio.3003971.r001

25 Feb 2026

Attachment Submitted filename: 159099_1_rebuttal_3650251_t9xxzf.docx

# Editor report: Decision Letter 0

**DOI:** 10.1371/journal.pbio.3003971.r002

**Authors:** Ines Alvarez-Garcia

20 Mar 2026

Dear Dr Motiani,

Thank you for submitting your manuscript entitled "IP3R2 mediated inter-organelle Ca2+ signaling suppresses melanophagy" for consideration as a Research Article by PLOS Biology.

Your manuscript has now been evaluated by the PLOS Biology editorial staff as well as by an academic editor with relevant expertise and I am writing to let you know that we would like to send your submission out for external peer review.

However, before we can send your manuscript to reviewers, we need you to complete your submission by providing the metadata that is required for full assessment. To this end, please login to Editorial Manager where you will find the paper in the 'Submissions Needing Revisions' folder on your homepage. Please click 'Revise Submission' from the Action Links and complete all additional questions in the submission questionnaire.

Once your full submission is complete, your paper will undergo a series of checks in preparation for peer review. After your manuscript has passed the checks it will be sent out for review. To provide the metadata for your submission, please Login to Editorial Manager (https://www.editorialmanager.com/pbiology) within two working days, i.e. by Mar 24 2026 11:59PM.

Feel free to email us at plosbiology@plos.org if you have any queries relating to your submission.

Kind regards,

Ines

--

Ines Alvarez-Garcia, PhD

Senior Editor

PLOS Biology

ialvarez-garcia@plos.org

# Author comment: Author response to Decision Letter 1

**DOI:** 10.1371/journal.pbio.3003971.r003

20 Mar 2026

Attachment Submitted filename: 159099_1_rebuttal_3650251_t9xxzf_auresp_1.docx

# Peer review: Decision Letter 1

**DOI:** 10.1371/journal.pbio.3003971.r004

**Authors:** Ines Alvarez-Garcia

8 May 2026

Dear Dr Motiani,

Thank you for your patience while we considered via Review Commons your revised manuscript entitled "IP3R2 mediated inter-organelle Ca2+ signaling suppresses melanophagy" for publication as a Research Article at PLOS Biology. Your revised study has been evaluated by the PLOS Biology editors, an Academic Editor and the three original reviewers.

The reviews are attached below. As you will see, all the reviewers appreciate the improvements made during the revision, however they still raise several remaining points that would need to be addressed before we can consider the manuscript for publication. Reviewer 1 asks for several clarifications and also thinks that the experiment in Fig. 8 showing that IP3R2 and TRPML1 knockdown decreases MLSA1 should be repeated using bafilomycin or GCN, given that MLSA1 is a TRPML agonist. Reviewer 2 thinks that the limitations of the study should be clearly discussed, and that the mechanistic shortcomings should be addressed by clearly indicating what findings are experimentally supported vs others that are correlative, prioritising the strongest and most rigorously supported conclusions and discussing more carefully the proposed proteasome-associated mechanism. In addition, this reviewer requests the rescue of mitochondrial Ca2+ uptake or direct manipulation of lysosomal Ca2+ buffering, and additional streamlining of the text to improve the flow. Finally, Reviewer 3 asks for the clarification of one point.

In light of the reviews, we would like to invite you to revise the work to thoroughly address the reviewers' reports. We would also want to note that, after discussing the reviews with the Academic Editor, we think that Reviewer 2's request on the rescue of mitochondrial Ca2+ uptake or direct manipulation of lysosomal Ca2+ buffering is essential for publication.

Given the extent of revision needed, we cannot make a decision about publication until we have seen the revised manuscript and your response to the reviewers' comments. Your revised manuscript is likely to be sent for further evaluation by all or a subset of the reviewers.

In addition to these revisions, you will need to complete some formatting changes, which you will receive in a follow up email. A member of our team will be in touch with a set of requests shortly.

We expect to receive your revised manuscript within 3 months. Please email us (plosbiology@plos.org) if you have any questions or concerns, or would like to request an extension.

At this stage, your manuscript remains formally under active consideration at our journal; please notify us by email if you do not intend to submit a revision so that we may withdraw it.

**IMPORTANT - SUBMITTING YOUR REVISION**

Your revisions should address the specific points made by each reviewer. Please submit the following files along with your revised manuscript:

1. A 'Response to Reviewers' file - this should detail your responses to the editorial requests, present a point-by-point response to all of the reviewers' comments, and indicate the changes made to the manuscript.

*NOTE: In your point-by-point response to the reviewers, please provide the full context of each review. Do not selectively quote paragraphs or sentences to reply to. The entire set of reviewer comments should be present in full and each specific point should be responded to individually, point by point.

You should also cite any additional relevant literature that has been published since the original submission and mention any additional citations in your response.

2. In addition to a clean copy of the manuscript, please also upload a 'track-changes' version of your manuscript that specifies the edits made. This should be uploaded as a "Revised Article with Changes Highlighted" file type.

3. Resubmission Checklist

When you are ready to resubmit your revised manuscript, please refer to this resubmission checklist: https://plos.io/Biology_Checklist

To submit a revised version of your manuscript, please go to https://www.editorialmanager.com/pbiology/ and log in as an Author. Click the link labelled 'Submissions Needing Revision' where you will find your submission record.

Please make sure to read the following important policies and guidelines while preparing your revision and fulfil the editorial requests:

a) *PLOS Data Policy*

Please note that as a condition of publication PLOS' data policy (http://journals.plos.org/plosbiology/s/data-availability) requires that you make available all data used to draw the conclusions arrived at in your manuscript. If you have not already done so, you must include any data used in your manuscript either in appropriate repositories, within the body of the manuscript, or as supporting information (N.B. this includes any numerical values that were used to generate graphs, histograms etc.). Please also indicate in each figure legend where the data can be found. For an example see here: http://www.plosbiology.org/article/info%3Adoi%2F10.1371%2Fjournal.pbio.1001908#s5

b) *Published Peer Review*

Please note while forming your response, if your article is accepted, you may have the opportunity to make the peer review history publicly available. The record will include editor decision letters (with reviews) and your responses to reviewer comments. If eligible, we will contact you to opt in or out. Please see here for more details:

https://blogs.plos.org/plos/2019/05/plos-journals-now-open-for-published-peer-review/

c) *Blot and Gel Data Policy*

Please provide the original, uncropped and minimally adjusted images supporting all blot and gel results reported in an article's figures or Supporting Information files. We will require these files before a manuscript can be accepted so please prepare them now, if you have not already uploaded them. Please carefully read our guidelines for how to prepare and upload this data: https://journals.plos.org/plosbiology/s/figures#loc-blot-and-gel-reporting-requirements

d) *Blurb*

Please also provide a blurb which (if accepted) will be included in our weekly and monthly Electronic Table of Contents, sent out to readers of PLOS Biology, and may be used to promote your article in social media. The blurb should be about 30-40 words long and is subject to editorial changes. It should, without exaggeration, entice people to read your manuscript. It should not be redundant with the title and should not contain acronyms or abbreviations. For examples, view our author guidelines: https://journals.plos.org/plosbiology/s/revising-your-manuscript#loc-blurb

e) *Protocols deposition*

To enhance the reproducibility of your results, we recommend that if applicable you deposit your laboratory protocols in protocols.io, where a protocol can be assigned its own identifier (DOI) such that it can be cited independently in the future. For instructions see: https://journals.plos.org/plosbiology/s/submission-guidelines#loc-materials-and-methods

Thank you again for your submission to our journal. We hope that our editorial process has been constructive thus far, and we welcome your feedback at any time. Please don't hesitate to contact us if you have any questions or comments.

Sincerely,

Ines

--

Ines Alvarez-Garcia, PhD

Senior Editor

PLOS Biology

ialvarez-garcia@plos.org

------------------------------------

Reviewers' comments

Rev. 1:

The revised manuscript has been much improved since the first submission. Nevertheless, some questions remain as follows:

Specific Comments:

1. My comment about figure panels 1F and 1J should actually have been 1H and 1J. I find it difficult to assess the relative effect of αMSH on IP3R2 expression as presented.

2. Unfortunately, my 2nd comment was also less than clear. While I do acknowledge that you measure IP3R mRNA, the relationship between mRNA and protein levels is rarely truly linear. Is IP3R2 the predominant isoform at the protein level? Closely related, rescue experiments in Fig 6 show that IP3R2 overexpression rescues a mitochondrial phenotype after its knockdown. Would IP3R1 or IP3R3 have been able to rescue also?

3. While I agree with the authors that all 3 IP3R isoforms are in the ER. The ER is not a homogeneous space and there are numerous examples of differences in subcellular localization. Are there differences in their association with melanosomes? Lysosomes? The PM?

4. Starting on page 24, there are a series of references to GFP/RFP ratio in reference to a probe that contains mCherry and eGFP. The authors should recognize that RFP is a very different protein from mCherry; as described, this is inaccurate.

5. Figure 6N reports an increase in the p-AMPK/AMPK ratio. While I agree that it shows that, why does the total AMPK level go down while the amount of phosphorylation stays the same?

6. Throughout the article, the term "leaky channels" is used, however, they are typically referred to as "leak channels"

7. There is a claim that IP3R2 channels downregulate TRPML1 activity. However, given that these channels are in different organelles, have the authors considered the possibility that this relates to calcium loading in lysosomes?

8. In figure 8E,F the authors demonstrate that IP3R2 and TRPML1 knockdown decreases MLSA1 stimulated calcium release. Since MLSA1 is a TRPML agonist, this is not appropriate. This experiment should be done with bafilomycin or GCN.

9. In reference to Fig 8G-H, the authors wrote "comparison to the control condition" however, that is quite vague. What was it compared with?

Rev. 2:

The revised manuscript is substantially strengthened relative to the prior version. The authors have added several important experiments, including additional validation of the melanophagy probes, autofluorescence controls, OPTN/RCHY1 loss-of-function analyses, TMEM165 loss-of-function experiments, positive controls for mitophagy and ER-phagy reporters, and PLA/TEM analyses of ER-lysosome proximity. These additions improve aspects of the technical rigor and mechanistic support for the central model. However, despite these improvements, I do not think all major concerns have been fully resolved. The manuscript remains mechanistically overextended, with several conclusions still relying heavily on indirect readouts, correlative relationships, or single-assay support. In some cases, the new data add additional mechanistic complexity without fully resolving the original interpretational concerns. More broadly, the study attempts to establish a very large interconnected pathway spanning ER calcium signaling, mitochondrial calcium uptake, bioenergetics, proteostasis, lysosomal calcium signaling, organelle contact sites, transcriptional regulation, and selective autophagy. While ambitious, the breadth of the proposed model ultimately exceeds the rigor with which several individual mechanistic links are established. In particular, the physiological relevance of IP3R2 downregulation, the proposed proteasome-melanophagy link, and the specificity of melanophagy induction remain insufficiently established for the level of mechanistic certainty claimed throughout the manuscript.

Major comments:

1. Physiological relevance of IP3R2 downregulation remains insufficiently established. The central experimental paradigm throughout the manuscript is IP3R2 knockdown. While the authors now include analysis of public transcriptomic datasets showing lower IP3R2 expression in lighter skin, this remains correlative and does not establish a physiological or pathological context in which IP3R2 activity is naturally reduced in melanocytes to trigger melanophagy. Given that IP3R2 is a major ER Ca2+ release channel, its silencing likely induces broad perturbations in ER Ca2+ handling, organelle communication, bioenergetics, and cellular stress pathways. Thus, it remains unclear whether the observed phenotype reflects a bona fide endogenous melanophagy-regulatory mechanism versus a broader stress response secondary to disrupted Ca2+ homeostasis. This limitation should be discussed much more explicitly, and claims regarding physiological relevance and translational potential should be tempered accordingly.

2. The manuscript remains mechanistically diffuse and overextended. The revised version further expands the proposed signaling network connecting IP3R2 loss to proteasomal dysfunction, melanophagy, mitochondrial Ca2+ depletion, AMPK/ULK1 activation, altered lysosomal pH, TMEM165 function, ER-lysosome proximity, TRPML1 activation, TFEB nuclear translocation, and OPTN/RCHY1 induction. While many individual observations are interesting, not all mechanistic links are supported with comparable rigor. Importantly, the manuscript increasingly relies on accumulation of partially supported pathway relationships rather than deeply establishing individual mechanistic steps. In several instances, one indirect readout is used to support another inferred mechanism, creating a chain of conclusions where the overall certainty exceeds that of the underlying experimental evidence. The manuscript would therefore benefit from a clearer distinction between experimentally demonstrated findings versus inferred pathway relationships, and from a more focused mechanistic framing prioritizing the strongest and most rigorously supported conclusions.

3. The proposed proteasome-melanophagy connection remains insufficiently supported. A central conceptual issue remains unresolved: IP3R2 knockdown stabilizes melanogenic proteins (DCT, Tyrosinase, GP100) while simultaneously increasing melanophagy readouts. Because these proteins are integral melanosomal components commonly used as indicators of melanosome abundance or turnover, this creates significant interpretational ambiguity. The authors propose impaired proteasomal degradation followed by compensatory melanophagy, but the supporting evidence remains limited. In particular, the MG132 experiments are difficult to interpret mechanistically. The observation that MG132 treatment decreases GP100/DCT levels is counterintuitive for a proteasome inhibitor and does not directly demonstrate impaired proteasomal degradation following IP3R2 knockdown. Alternative explanations (incl. indirect stress effects, altered melanosome homeostasis, compensatory lysosomal/autophagic degradation, toxicity, or altered protein extraction/detection) remain plausible.

Furthermore, how do the authors explain the apparent increase in endogenous MCU protein levels under IP3R2 silencing conditions, which appears further enhanced following MG132 treatment (see Response to Rev 2_Comment 6))? This observation is mechanistically important because mitochondrial Ca2+ uptake is nevertheless reduced under these conditions, implying that impaired uptake is not simply attributable to reduced MCU abundance. Instead, the data may indicate compensatory upregulation of MCU, altered ER-mitochondria Ca2+ microdomain signaling, mitochondrial dysfunction, or broader proteostasis remodeling. The relationship between MCU expression, mitochondrial Ca2+ uptake capacity, and the proposed proteasome-associated mechanism should therefore be discussed more carefully (see below comment also).

Taken together, the current data do not yet provide sufficiently direct evidence to support the proposed proteasome-centered model. Direct proteasome activity measurements, ubiquitination analyses, or compartment-specific turnover assays would be needed to substantiate these conclusions more convincingly. At minimum, the proteasome-related conclusions should be substantially tempered.

4. Mechanistic causality remains incomplete despite additional experiments. The newly added TMEM165 co-silencing experiments and lysosomal Ca2+ kinetic analyses strengthen parts of the proposed pathway. However, several key mechanistic relationships remain largely associative. For example, the manuscript supports a role for altered mitochondrial and lysosomal Ca2+ dynamics in melanophagy induction, but direct necessity/sufficiency experiments remain limited. Rescue of mitochondrial Ca2+ uptake or direct manipulation of lysosomal Ca2+ buffering would substantially strengthen causal interpretation. While MCU overexpression partially addresses this point, unchanged or elevated endogenous MCU protein levels under IP3R2 knockdown conditions further suggest that altered mitochondrial Ca2+ uptake likely reflects changes in local ER-mitochondria Ca2+ transfer, channel regulation, mitochondrial state, or bioenergetics rather than simple limitation of uptake machinery abundance. As written, some pathway steps are presented with greater certainty than the data currently support.

5. Specificity for melanophagy over broader stress-associated autophagy remains insufficiently explained. The addition of positive controls for mitophagy and ER-phagy reporters improves the rigor of these assays. However, the manuscript still does not adequately explain why broad perturbation of ER Ca2+ signaling and cellular bioenergetics would selectively induce melanosome degradation while suppressing or sparing other selective autophagy pathways. The current data support altered melanophagy-associated phenotypes, but the claim of mechanistic specificity for melanophagy remains stronger than the available evidence.

6. Interpretation of the melanophagy probes still requires substantial caution. The authors now state that melanosome autofluorescence was controlled by imaging non-transfected cells under identical acquisition settings and subtracting background signals during quantitation. While this clarification is helpful, the issue remains incompletely resolved experimentally. Melanosome autofluorescence is highly heterogeneous, punctate, and biologically variable, particularly across conditions that themselves alter pigmentation, melanosome abundance, and lysosomal pH. Under such conditions, simple background subtraction may not be sufficient to exclude spectral contribution to GFP/mCherry/mKeima-based measurements. Importantly, representative autofluorescence control images, quantitative baseline fluorescence measurements, or spectral unmixing analyses are not provided.

More broadly, these probes remain pH-sensitive fluorescence reporters used in highly pigmented cells, while the proposed mechanism itself alters lysosomal pH. Thus, interpretation remains potentially influenced by lysosomal acidification state, melanosome autofluorescence, probe localization, and expression levels. The reporters are useful tools, but the manuscript should avoid presenting them as fully quantitative measures of melanophagy flux without additional calibration to actual lysosomal delivery or degradation rates.

7. Evidence for endogenous melanophagy pathway engagement remains limited. The authors now include HMB45/LC3 colocalization analyses as supplementary evidence supporting melanophagy induction (Suppl. Fig. 3). While this addition is helpful, the image quality and analytical approach limit interpretability. In particular, insufficient resolution and reliance on global PCC measurements does not convincingly establish discrete autophagosomal association or engulfment of endogenous melanosomes.

The newly added OPTN/RCHY1 co-silencing experiments strengthen the argument that these factors contribute functionally downstream of IP3R2 loss. However, these analyses rely primarily on the same fluorescence reporter systems whose quantitative interpretation remains a central issue in the manuscript. In addition, direct evidence that endogenous melanophagy machinery is engaged under these conditions remains limited. The manuscript still lacks direct demonstration of OPTN/RCHY1 recruitment to melanosomes. While prior studies have implicated OPTN and RCHY1 in melanophagy, pathway engagement downstream of IP3R2 silencing in the present system remains inferred rather than directly demonstrated.

Minor comments:

- The newly added PLA controls and quantitative TEM analyses substantially strengthen the ER-lysosome proximity data. However, the observation that ER-lysosome distance decreases without a corresponding increase in contact frequency is conceptually interesting and deserves more detailed discussion.

- Some claims regarding therapeutic relevance for pigmentary disorders and melanoma remain speculative, as no disease model or physiological stress paradigm is examined.

- The manuscript remains exceptionally dense, with many mechanistic branches introduced in rapid succession. Additional streamlining and prioritization of the central mechanistic findings would improve clarity and accessibility.

Overall assessment:

This revised manuscript is improved relative to the prior version and presents a technically ambitious study linking IP3R2-dependent inter-organelle Ca2+ signaling to melanophagy-associated phenotypes and pigmentation regulation. Several newly added experiments strengthen aspects of the mechanistic model. However, in its current form, the manuscript still makes mechanistic claims that exceed the strength of the supporting evidence in several areas. The principal concerns relate to the unclear physiological relevance of IP3R2 downregulation, incomplete mechanistic causality, unresolved interpretation of the proteasome-melanophagy relationship, insufficiently supported claims regarding selective melanophagy specificity, and limited orthogonal evidence for endogenous melanophagy pathway engagement. Overall, the study illustrates the risk that broad pathway expansion can substitute for rigorous establishment of individual mechanistic steps. For a journal such as PLOS Biology, I would expect either substantially stronger causal evidence for the full pathway or a more focused and carefully tempered mechanistic framing that more clearly distinguishes direct findings from broader model speculation.

Rev. 3: Antony Galione – note that this reviewer has signed the review.

This manuscript had previously been refereed as a preprint before submission to PLOS Biology, and the authors have been joined by an additional author for this submission. The authors have outlined their responses to the pre-review and added new data to address the points made.

The principal finding is that silencing of the type 2 IP3 receptor evokes a pathway leading to melanophagy, where by melanosomes are degraded. Thus it is proposed that IP3R2-mediated calcium fluxes maintain the presence of melanosomes and hence pigmentation. The authors present a model by which inter-organelle calcium transfer between the ER, mitochondria and lysosomes play an important role in this process.

They have measured calcium in organelle compartments such as mitochondria and lysosomes and assessed Ca2+ loading and release in IP3R2 silenced cells and the effects of IP3 linked agonists or membrane permeant IP3. These experiments are generally convincing, but complex since the role of MCU, TMEM165, TRPML1 are all assessed either genetically or pharmacologically. These however, often rely on one KD experiment or one pharmacological agent.

In general I am satisfied with the responses to the referees for the preprint reviews (I was one of them) which were attached. It goes some way to strengthen what is a complex paper.

The link with the zebrafish model in vivo is commended, and I note there is now data showing the effect of activating TRPML1 pharmacologically.

I have one major question:

1. B16 were activated by histamine to produce a calcium response via IP3 presumably via H1 receptors. Does aMSH do the same?

# Author comment: Author response to Decision Letter 2

**DOI:** 10.1371/journal.pbio.3003971.r005

23 Jul 2026

Attachment Submitted filename: Reviewer_Comments_IP3R2_Draft5 210726.docx

# Editor report: Decision Letter 2

**DOI:** 10.1371/journal.pbio.3003971.r006

**Authors:** Ines Alvarez-Garcia

6 Aug 2026

Dear Dr Motiani,

Thank you for your patience while we considered your revised manuscript "IP3R2 mediated inter-organelle Ca2+ signaling suppresses melanophagy" for publication as a Research Article at PLOS Biology. This revised version of your manuscript has been evaluated by the PLOS Biology editors the Academic Editor.

Based on our Academic Editor's assessment of your revision, we are likely to accept this manuscript for publication. Please also make sure to address the following data and other policy-related requests.

IMPORTANT: Please ensure that your next revision addresses the following points:

-----------------------------

**Title:

We would like to tweak the title in accordance with our preferences for broad accessibility and style. Is the following alternative version acceptable to you?

"IP3R2-mediated inter-organelle calcium signaling suppresses melanosome degradation"

**Financial disclosure statement:

Please add links to the funding agencies in the Financial Disclosure statement in the manuscript details.

**Ethics:

Please move the Ethics Statement to the beginning of the Materials and Methods section. Please also include the specific national or international regulations/guidelines to which your animal care protocols adhered.

**Data:

Thank you for providing the underlying data supporting the figures. However, we did not see data relating to Fig. S9E,N,P within S2 Data. Please double check that these are included, and modify if necessary.

**Gels:

As you know, we require the original, uncropped and minimally adjusted images supporting all blot and gel results reported in the Figures. We see that you have included relevant images in the Raw Images file, although we also expected to see something for Fig. 8C.

As a reminder, here are our guidelines for gel and blot reporting: https://journals.plos.org/plosbiology/s/figures#loc-blot-and-gel-reporting-requirements

**Code availability:

Per journal policy, if you have generated any custom code or scripts during the course of this investigation, we require that you make it available without restrictions. Please ensure that the code is sufficiently well documented and reusable, and that your Data Statement in the Editorial Manager submission system accurately describes where your code can be found. Please also ensure that you choose a license for your code and include a Readme file.

Please note that we cannot accept sole deposition of code in GitHub, as this could be changed after publication. However, you can archive this version of your publicly available GitHub code to Zenodo. Once you do this, it will generate a DOI number, which you will need to provide in the Data Accessibility Statement (you are welcome to also provide the GitHub access information). See the process for doing this here: https://docs.github.com/en/repositories/archiving-a-github-repository/referencing-and-citing-content

-----------------------------

As you address these items, please take this last chance to review your reference list to ensure that it is complete and correct. If you have cited papers that have been retracted, please include the rationale for doing so in the manuscript text, or remove these references and replace them with relevant current references. Any changes to the reference list should be mentioned in the cover letter that accompanies your revised manuscript.

In addition to these revisions, you may need to complete some formatting changes, which you will receive in a follow up email. A member of our team will be in touch with a set of requests shortly. If you do not receive a separate email within a few days, please assume that checks have been completed, and no additional changes are required.

We expect to receive your revised manuscript within two weeks.

To submit your revision, please go to https://www.editorialmanager.com/pbiology/ and log in as an Author. Click the link labelled 'Submissions Needing Revision' to find your submission record. Your revised submission must include the following:

- a cover letter that should detail your responses to any editorial requests, if applicable, and whether changes have been made to the reference list

- a Response to Reviewers file that provides a detailed response to the reviewers' comments (if applicable, if not applicable please do not delete your existing 'Response to Reviewers' file.)

- a track-changes file indicating any changes that you have made to the manuscript.

NOTE: If Supporting Information files are included with your article, note that these are not copyedited and will be published as they are submitted. Please ensure that these files are legible and of high quality (at least 300 dpi) in an easily accessible file format. For this reason, please be aware that any references listed in an SI file will not be indexed. For more information, see our Supporting Information guidelines:

https://journals.plos.org/plosbiology/s/supporting-information

*Published Peer Review History*

Please note that you may have the opportunity to make the peer review history publicly available. The record will include editor decision letters (with reviews) and your responses to reviewer comments. If eligible, we will contact you to opt in or out. Please see here for more details:

https://plos.org/published-peer-review-history/

*Press*

Should you, your institution's press office or the journal office choose to press release your paper, please ensure you have opted out of Early Article Posting on the submission form. We ask that you notify us as soon as possible if you or your institution is planning to press release the article.

*Protocols deposition*

To enhance the reproducibility of your results, we recommend that if applicable you deposit your laboratory protocols in protocols.io, where a protocol can be assigned its own identifier (DOI) such that it can be cited independently in the future. Additionally, PLOS ONE offers an option for publishing peer-reviewed Lab Protocol articles, which describe protocols hosted on protocols.io. Read more information on sharing protocols at https://plos.org/protocols?utm_medium=editorial-email&utm_source=authorletters&utm_campaign=protocols

Please do not hesitate to contact me should you have any questions.

Sincerely,

Taylor Hart, PhD -- on behalf of my colleague Ines, who is out of the office.

Associate Editor, PLOS Biology

thart@plos.org

Ines

Ines Alvarez-Garcia, PhD

Senior Editor

ialvarez-garcia@plos.org

PLOS Biology

# Author comment: Author response to Decision Letter 3

**DOI:** 10.1371/journal.pbio.3003971.r007

11 Aug 2026

Attachment Submitted filename: Reviewer_Comments_IP3R2_Draft5_210726_auresp_3.docx

# Editor report: Decision Letter 3

**DOI:** 10.1371/journal.pbio.3003971.r008

**Authors:** Ines Alvarez-Garcia

12 Aug 2026

Dear Dr Motiani,

Thank you for the submission of your revised Research Article "IP3R2-mediated inter-organelle calcium signaling suppresses melanosome degradation" for publication in PLOS Biology. On behalf of my colleagues and the Academic Editor, Colin Jamora, I am pleased to say that we can in principle accept your manuscript for publication, provided you address any remaining formatting and reporting issues. These will be detailed in an email you should receive within 2-3 business days from our colleagues in the journal operations team; no action is required from you until then. Please note that we will not be able to formally accept your manuscript and schedule it for publication until you have completed any requested changes.

Please take a minute to log into Editorial Manager at http://www.editorialmanager.com/pbiology/, click the "Update My Information" link at the top of the page, and update your user information to ensure an efficient production process.

PRESS

We frequently collaborate with press offices. If your institution or institutions have a press office, please notify them about your upcoming paper at this point, to enable them to help maximise its impact. If the press office is planning to promote your findings, we would be grateful if they could coordinate with biologypress@plos.org. If you have previously opted in to the early version process, we ask that you notify us immediately of any press plans so that we may opt out on your behalf.

We also ask that you take this opportunity to read our Embargo Policy regarding the discussion, promotion and media coverage of work that is yet to be published by PLOS. As your manuscript is not yet published, it is bound by the conditions of our Embargo Policy. Please be aware that this policy is in place both to ensure that any press coverage of your article is fully substantiated and to provide a direct link between such coverage and the published work. For full details of our Embargo Policy, please visit http://www.plos.org/about/media-inquiries/embargo-policy/.

Thank you again for choosing PLOS Biology for publication and supporting Open Access publishing. We look forward to publishing your study.

Sincerely,

Taylor Hart, PhD -- on behalf of my colleague Ines, who is out of the office.

Associate Editor, PLOS Biology

thart@plos.org

---

Ines Alvarez-Garcia, PhD, PhD

Senior Editor

PLOS Biology

ialvarez-garcia@plos.org
